# Towards precision medicine for anxiety disorders: objective assessment, risk prediction, pharmacogenomics, and repurposed drugs

**DOI:** 10.1038/s41380-023-01998-0

**Published:** 2023-03-07

**Authors:** K. Roseberry, H. Le-Niculescu, D. F. Levey, R. Bhagar, K. Soe, J. Rogers, S. Palkowitz, N. Pina, W. A. Anastasiadis, S. S. Gill, S. M. Kurian, A. Shekhar, A. B. Niculescu

**Affiliations:** 1 Department of Psychiatry, Indiana University School of Medicine, Indianapolis, IN USA; 2 Stark Neuroscience Research Institute, Indiana University School of Medicine, Indianapolis, IN USA; 3 Indianapolis VA Medical Center, Indianapolis, IN USA; 4 Scripps Health and Department of Molecular Medicine, Scripps Research, La Jolla, CA USA; 5 Present Address: Yale School of Medicine, New Haven, CT USA; 6 Present Address: Cincinnati Children’s Hospital, University of Cincinnati College of Medicine, Cincinnati, OH USA; 7 Present Address: Office of the Dean, University of Pittsburgh School of Medicine, Pittsburgh, PA USA

**Keywords:** Genetics, Biomarkers

## Abstract

Anxiety disorders are increasingly prevalent, affect people’s ability to do things, and decrease quality of life. Due to lack of objective tests, they are underdiagnosed and sub-optimally treated, resulting in adverse life events and/or addictions. We endeavored to discover blood biomarkers for anxiety, using a four-step approach. First, we used a longitudinal within-subject design in individuals with psychiatric disorders to discover blood gene expression changes between self-reported low anxiety and high anxiety states. Second, we prioritized the list of candidate biomarkers with a Convergent Functional Genomics approach using other evidence in the field. Third, we validated our top biomarkers from discovery and prioritization in an independent cohort of psychiatric subjects with clinically severe anxiety. Fourth, we tested these candidate biomarkers for clinical utility, i.e. ability to predict anxiety severity state, and future clinical worsening (hospitalizations with anxiety as a contributory cause), in another independent cohort of psychiatric subjects. We showed increased accuracy of individual biomarkers with a personalized approach, by gender and diagnosis, particularly in women. The biomarkers with the best overall evidence were GAD1, NTRK3, ADRA2A, FZD10, GRK4, and SLC6A4. Finally, we identified which of our biomarkers are targets of existing drugs (such as a valproate, omega-3 fatty acids, fluoxetine, lithium, sertraline, benzodiazepines, and ketamine), and thus can be used to match patients to medications and measure response to treatment. We also used our biomarker gene expression signature to identify drugs that could be repurposed for treating anxiety, such as estradiol, pirenperone, loperamide, and disopyramide. Given the detrimental impact of untreated anxiety, the current lack of objective measures to guide treatment, and the addiction potential of existing benzodiazepines-based anxiety medications, there is a urgent need for more precise and personalized approaches like the one we developed.

## Introduction

“Man is not worried by real problems so much as by his imagined anxieties about real problems.”

— Epictetus

Anxiety is increased reactivity in anticipation of events that are perceived as potentially deleterious, overwhelming, or challenging. Psychiatric patients may have increased anxiety, as well as increased reasons for anxiety, due to their adverse life trajectory. As such, they may be a high-yield population in which to try to identify blood biomarkers for anxiety that are generalizable and trans-diagnostic. Such markers would eliminate subjectivity from assessments, provide some indication of risk, and help guide treatments [1]. First, we used a powerful longitudinal within-subject design in individuals with psychiatric disorders to discover blood gene expression changes between self-reported low anxiety and high anxiety states, as measured by a visual analog scale we developed, the Simplified Anxiety Scale (SAS-4), similar to our previously published Simplified Mood Scale (SMS-7) [2]. Second, we prioritized the list of candidate biomarkers with a Bayesian-like Convergent Functional Genomics approach, comprehensively integrating previous human and animal model evidence in the field, from us and others. Third, we validated our top biomarkers from discovery and prioritization in an independent cohort of psychiatric subjects with clinically severe anxiety. We prioritized a list of 95 candidate biomarkers that had the most evidence from the first three steps. Fourth, we tested if these candidate biomarkers are able to predict anxiety severity state, and future clinical worsening (hospitalizations with anxiety as the primary cause), in another independent cohort of psychiatric subjects. We tested the biomarkers in all subjects in the test cohort, as well as in a more personalized fashion by gender and psychiatric diagnosis, showing increased accuracy with the personalized approach, particularly in women. Fifth, we analyzed the biological pathways and networks the biomarkers are involved in, as well as which of our top biomarkers have evidence for involvement in other psychiatric and related disorders. Sixth, we identified which of our biomarkers are targets of existing drugs and thus can be used for pharmacogenomic matching of patient to treatment, and measuring of response to treatment. We also used the top biomarkers gene expression signature to identify existing medications used for other indications, as well as natural compounds, that could be repurposed for treating anxiety.

Current medication treatments for anxiety (e.g., SSRIs, SNRIs, benzodiazepines, antihistamines, etc.) do not work well in everybody (e.g., low response/remission rates, trial-and-error prescription, problematic side effects, etc.) [3]. Matching the right individuals to the right medications using their biomarker profile is a key actionable outcome of our work.

## Materials and methods

### Cohorts

**Fig. 1:**
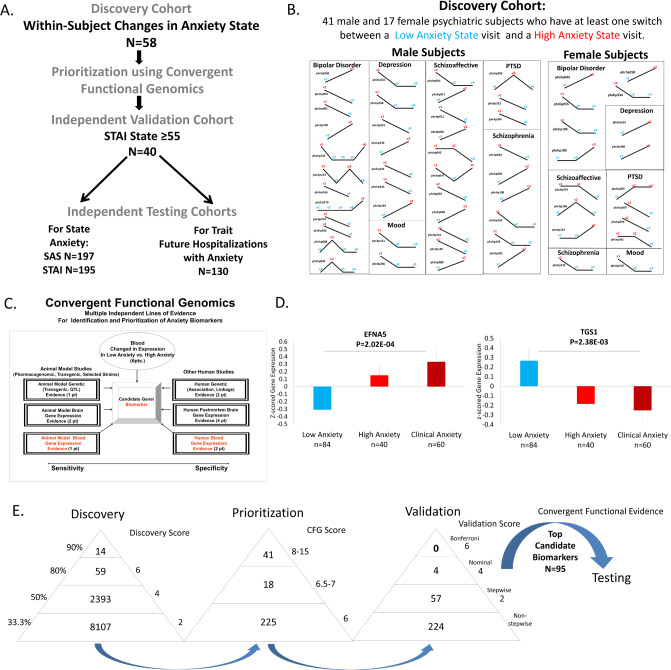
Steps 1–3: Discovery, prioritization, and validation of biomarkers for anxiety. **A** Cohorts used in study, depicting flow of discovery, prioritization, validation of biomarkers from each step and independent testing cohorts. **B** Discovery cohort longitudinal within-subject analysis. Phchp### is study ID for each subject. V# denotes visit number. Red are high anxiety visits and blue are low anxiety visits. **C** Convergent Functional Genomics evidence. **D** In the validation step biomarkers are assessed for stepwise change from the validation group with severe Anxiety, to the discovery groups of subjects with high Anxiety, low Anxiety, to the validation group with severe Anxiety, using ANOVA. N = number of testing visits. The histograms depict a top increased and a top decreased biomarker in validation. **E** Scoring at each of the steps. *Discovery* probesets are identified based on their score for tracking anxiety with a maximum of 6 points (33% (2 pt), 50% (4 pt) and 80% (6 pt)). *Prioritization* with CFG for prior evidence of involvement in anxiety disorders. In the prioritization step probesets are converted to their associated genes using Affymetrix annotation and GeneCards. Genes are prioritized and scored using CFG for anxiety evidence, with a maximum of 12 points. Genes scoring at least 6 points out of a maximum possible of 18 total internal and external scores points are carried to the validation step. *Validation* in an independent cohort of psychiatric patients with clinically severe anxiety (STAI State ≥ 55 and SAS-4 > =60). Four biomarkers were nominally significant, and 57 biomarkers were stepwise changed. We selected for further testing in independent cohorts the top candidate biomarkers, with a total score after the first 3 steps (CFE3) of 8 and above (*n* = 95 biomarkers).

Our study utilized 3 independent cohorts: discovery (major psychiatric disorders with changes in *state* anxiety), validation (major psychiatric disorders with clinically severe anxiety), and testing (an independent major psychiatric disorders cohort for predicting *state* anxiety, and for predicting *trait* anxiety (future hospitalization with anxiety as the primary reason) (Fig. 1A).

The psychiatric subjects are part of a larger longitudinal cohort of adults that we are continuously collecting [4–6]. Subjects were recruited from the patient population at the Indianapolis VA Medical Center. All subjects understood and signed informed consent forms detailing the research goals, procedure, caveats, and safeguards, per IRB-approved protocol. Subjects completed diagnostic assessments and extensive structured neuropsychological testing at each testing visit, 3–6 months apart or whenever a new psychiatric hospitalization occurred. At each testing visit, they received a series of rating scales, including a self-report visual analog scale (1–100) for quantitatively assessing *state* anxiety at that particular moment in time (Simplified Anxiety Scale- SAS-4). This 4-item scale looks at anxiety overall, as well as fear, anger, and uncertainty. Each of the items are a VAS of 0 to 100, related to that moment in time. As such, it generates temporal, quantitative, and targeted data.

At each testing visit we collected whole blood (10 ml) in two RNA-stabilizing PAXgene tubes, labeled with an anonymized ID number, and stored at −80 °C in a locked freezer until the time of future processing. Whole-blood RNA was extracted for microarray gene expression studies from the PAXgene tubes, as detailed below.

For this study, our within-subject discovery cohort consisted of 58 subjects (41 males, 17 females) with multiple testing visits, who each had at least one diametric change in anxiety *state* from low anxiety *state* (SAS-4 score of ≤40/100) to a high anxiety *state* (SAS-4 score of ≥60/100), or vice versa, from one testing visit to another (Figs. 1A, B and S1). There were 2 subjects with 5 visits each, 3 subjects with 4 visits each, 21 subjects with 3 visits each, and 32 subjects with 2 visits each resulting in a total of 149 blood samples for subsequent gene expression microarray studies (Fig. 1A, B, and Table S1).

Our independent validation cohort, in which the top candidate biomarker findings were validated for being even more changed in expression, consisted of 40 subjects (32 male and 8 female) with clinically severe anxiety (SAS-4 scores ≥60, and concordant high anxiety STAI State scores ≥ 55) (Table S1).

For testing the biomarkers, we used an independent test cohort.

For state predictions, we predicted high anxiety state (SAS-4 ≥ 60) (161 male and 36 female subjects) and clinically severe anxiety (STAI ≥ 55) (159 male and 36 female subjects) (Fig. 1 and Table S1).

For *trait* predictions of future hospitalizations with anxiety as a contributory reason (Fig. 1 and Table S1), we used a subset of the independent test cohort for which we had longitudinal follow-up with electronic medical records. The subjects’ subsequent number of hospitalizations with anxiety was tabulated from electronic medical records.

#### Medications

The subjects in our study were all diagnosed with various psychiatric disorders (Table S1) and had various medical co-morbidities. Their medications were listed in their electronic medical records and documented by us at the time of each testing visit. Medications can have a strong influence on gene expression. However, there was no consistent pattern of any particular type of medication. Our subjects were on a wide variety of different medications, psychiatric and non-psychiatric. Furthermore, the independent validation and testing cohort’s gene expression data was Z-scored by gender and by diagnosis before being combined, to normalize for any such effects. Some subjects may be non-compliant with their treatment and may have changes in medications or drugs of abuse not reflected in their medical records. Our goal is to find biomarkers that track anxiety, regardless if the reason for it is internal biology or it is driven by external medications or drugs. In fact, one would expect some of these biomarkers to be targets of medications, as we show in this paper. Furthermore, the prioritization step that occurs after the discovery step is based on a field-wide convergence with literature that includes genetic data and animal model data, that are unrelated to medication effects. Overall, the discovery, validation, and replication by testing in independent cohorts of the biomarkers, with our design, occurs despite the subjects having different genders, diagnoses, being on various different medications, and other variables.

### Blood gene expression experiments

#### RNA extraction

Whole blood (2.5 ml) was collected into each PaxGene tube by routine venipuncture. PaxGene tubes contain proprietary reagents for the stabilization of RNA. Total RNA was extracted and processed as previously described [4–6].

#### Microarrays

Microarray work was carried out using previously described methodology [4–7].

Of note, all genomic data was normalized (RMA for technical variability, then z-scoring for biological variability), by gender and psychiatric diagnosis, before being combined and analyzed.

See Supplementary Information for rest of “Materials and Methods”.

## Results

In Step 1 Discovery, we identified candidate blood gene expression biomarkers that: 1. change in expression in blood between self-reported low anxiety and high anxiety states, 2. track the anxiety state across visits in a subject, and 3. track the anxiety state in multiple subjects. We used a visual analog measure for anxiety state (SAS-4). At a phenotypic level, the SAS-4 quantitates anxiety state at a particular moment in time, and normalizes anxiety measurements in each subject, comparing them to the lowest and highest anxiety that the subject ever experienced (Fig. S1). It has a moderate to strong correlation (*R* = 0.67, *p* < 0.0001) with a current clinical scale for anxiety state (the STAI State, Fig. S2).

We used a powerful within-subject and then across-subject design in a longitudinally followed cohort of subjects (*n* = 58 subjects, with 149 visits) who displayed at least a 50% change in the anxiety measure (from below 40/100 to above 60/100) between at least two consecutive testing visits, to identify differentially expressed genes that track anxiety state. Using our 33% of maximum raw score threshold (internal score of 2 pt) [5, 6], we identified 10,573 unique probesets (corresponding to 7195 unique genes) from Affymetrix Absent/Present (AP) analyses and Differential Expression (DE) analyses (Fig. 1D). These were carried forward to the prioritization step. This represents approximately a fivefold enrichment of the 54,625 probesets on the Affymetrix array.

In Step 2 Prioritization, we used a Convergent Functional Genomics (CFG) approach to prioritize the candidate biomarkers identified in the discovery step (33% cutoff, internal score of ≥2 pt.) by using prior published literature evidence (genetic, gene expression, and proteomic), from human and animal model studies, for involvement in anxiety disorders (Fig. 1E and Table S2). There were 284 probesets (corresponding to 238 unique genes) that had a total score (combined discovery score and prioritization CFG score) of 6 and above. These were carried forward to the validation step. This represents approximately a tenfold enrichment of the probesets on the Affymetrix array.

In Step 3 Validation, we validated the prioritized candidate biomarkers for change in clinically severe anxiety, in a demographically matched cohort of (*n* = 40 clinically severe anxiety) by assessing which markers were stepwise changed in expression from low anxiety in discovery cohort, to high anxiety in discovery cohort, to clinically severe anxiety in validation cohort (Fig. 1). Of the 284 probesets after the prioritization step, 224 probesets were not stepwise changed, and 57 were stepwise changed. Of these, four probesets (corresponding to four unique genes) were nominally significant.

**Fig. 2:**
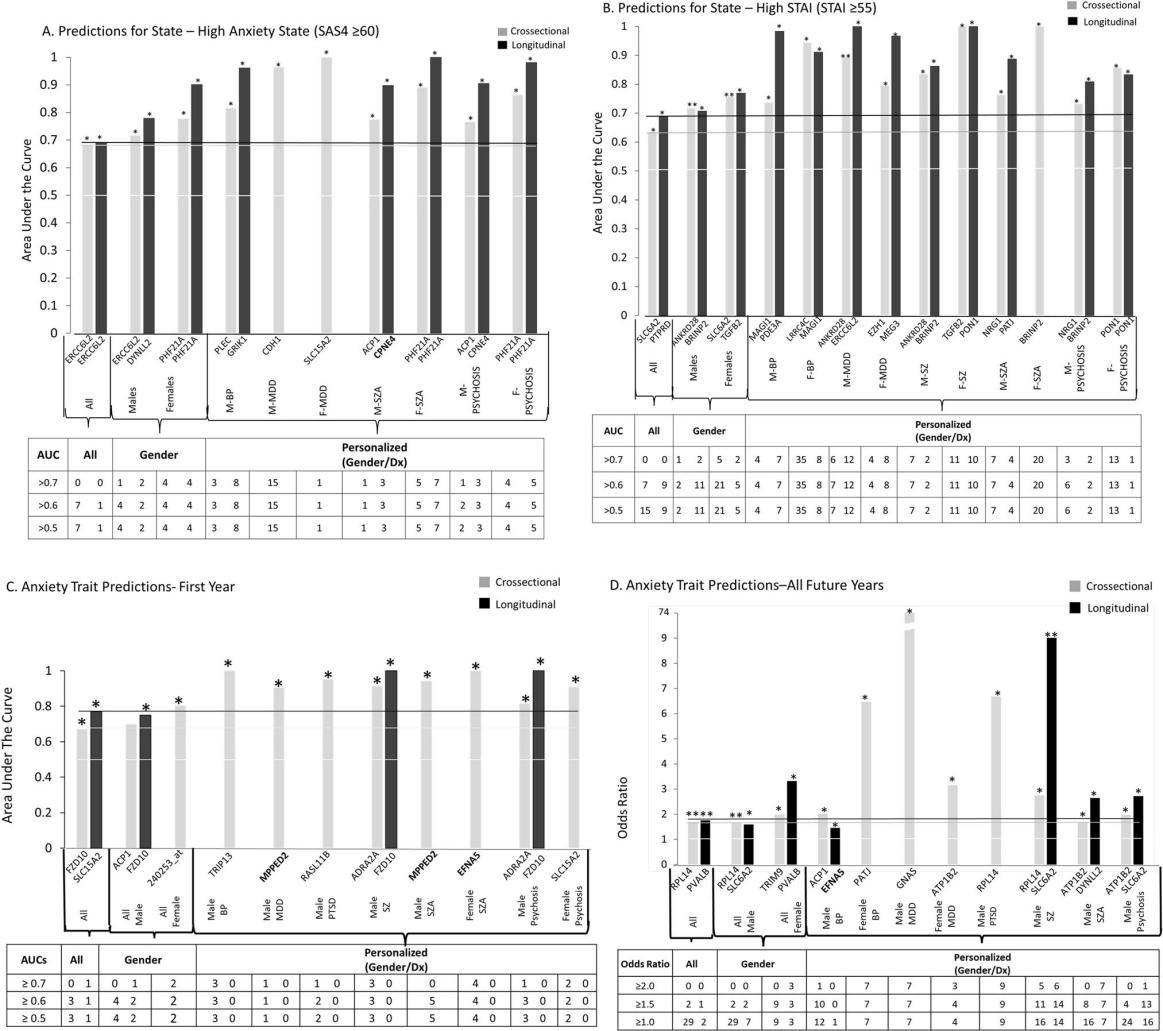
Best Single Biomarkers Predictors for Anxiety, State and Trait. From top candidate biomarkers after Steps 1–3 (Discovery, Prioritization, Validation-Bold) (*n* = 95). Bar graph shows best predictive biomarkers in each group. All markers with * are nominally significant *p* < 0.05. Table underneath the figures displays the actual number of biomarkers for each group whose ROC AUC *p* values (**A**–**C**) and Cox Odds Ratio *p* values (**D**) are at least nominally significant. Some gender and diagnosis groups are missing from the graph as they did not have any significant biomarkers or that the cohort was too small with limited data for the z-scoring by gender-dx. Cross-sectional is based on levels at one visit. Longitudinal is based on levels at multiple visits (integrates levels at most recent visit, maximum levels, slope into most recent visit, and maximum slope). Dividing lines represent the cutoffs for a test performing at chance levels (white), and at the same level as the best biomarkers for all subjects in cross-sectional (gray) and longitudinal (black) based predictions. Biomarkers perform better than chance. Biomarkers performed better when personalized by gender and diagnosis. * nominally significant. ** survived Bonferroni correction for the number of candidate biomarkers tested.

Adding the scores from the first three steps into an overall convergent functional evidence (CFE) score (Fig. 1), we ended up with a list of 95 top candidate biomarkers (n = 82 genes, 95 probesets), that had a CFE3 score ≥8, equal or better to 33% of the maximum possible score of 24 after the first three steps, which we decided to use as an empirical cutoff. This represents approximately an over 500-fold enrichment of the probesets on the Affymetrix array. These 95 top candidate biomarkers were carried forward into analyses for understanding biological underpinnings. They were also tested in Step 4 for clinical utility/predictive ability in additional independent cohorts (Fig. 2 and Table 1).

**Table 1:** Top Anxiety Biomarkers: Convergent Functional Evidence (CFE).

Symbol/Gene Name	Probesets	Step 1 Discovery (Direction of Change in High Anxiety) Method/ Score/%	Step 2 Prioritization Convergent Functional Genomics (CFG) Evidence For Involvement in Anxiety Score	Step 3 Validation ANOVA p-value/Score	Step 4 Significant Predictions of High Anxiety State ROC AUC/ p-value 3 pts ALL 2pts Gender 1pts Gender /Dx	Step 4 Significant Predictions of High STAI State ROC AUC/ p-value 3 pts ALL 2pts Gender 1pts Gender /Dx	Step 4 Significant Prediction of First Year Hosp. for Anxiety ROC AUC/p-value 3 pts ALL 2pts Gender 1pts Gender/Dx	Step 4 Significant Predictions of Future Hosp for Anxiety OR/p-value 3 pts ALL 2pts Gender 1pts Gender /Dx	Other Psychiatric and Related Disorders Evidence	Drugs that Modulate the Biomarker in Opposite Direction to High Anxiety	CFE Polyevidence Score for Involvement in Anxiety (Based on Steps 1–4)
**GAD1** Glutamate Decarboxylase 1	205278_at	(I) DE/4 62.3%	11	6.79E-01/0 Not Stepwise		**ALL** **C:** (42/486) 0.58/3.94E-02 **Gender** **Females** **C:** (17/88) 0.65/2.75E-02 **Gender-dx** **F-PSYCHOSIS** **C:** (5/28) 0.77/2.94E-02 **F-SZ** **C:** (4/8) 0.94/2.17E-02	**Gender-dx** **M-PSYCHOSIS** **C:** (7/74) 0.69/4.73E-02 **M-SZA** **C:** (3/32) 0.79/4.96E-02	**ALL** **C:** (70/318) 1.3/5.52E-03 **Gender** Males **C:** (59/273) 1.34/2.80E-03 **Gender-dx** **M-BP** **C:** (13/96) 1.69/2.12E-02 **M-PSYCHOSIS** **C:** (37/121) 1.33/9.36E-03 **M-SZA** **C:** (23/58) 1.58/9.14E-03	Alcohol Withdrawal Autism BP Depression Intellectual Disability MDD Mood Disorders NOS Phencyclidine PTSD Suicide SZ	Carbamazepine Lithium Omega-3 fatty acids Valproate	22
**NTRK3** Neurotrophic Receptor Tyrosine Kinase 3	215311_at	(I) DE/4 53.2%	4	3.26E-01/2 Stepwise	**Gender** **Males** **L:** (4/246) 0.77/2.98E-02 **Gender-dx** **M-PSYCHOSIS** **L:** (2/102) 0.9/2.83E-02 **M-SZA** **L:** (2/46) 0.85/4.75E-02	**ALL** **C:** (42/486) 0.59/3.04E-02 **L:** (21/291) 0.61/4.83E-02 **Gender** **Females** **C:** (17/88) 0.68/1.20E-02 **L:** (10/52) 0.7/2.85E-02 **Gender-dx** **F-BP** **C:** (4/31) 0.89/6.66E-03 **M-MDD** **L:** (4/31) 0.82/1.96E-02	**Gender-dx** F-PSYCHOSIS **C:** (2/13) 0.91/3.78E-02 **Gender-dx** **F-SZA** **C:** (2/6) 1/3.20E-02	**ALL** **C:** (70/318) 1.23/9.38E-03 Gender Females **C:** (11/45) 1.84/1.60E-02	Aging Alcohol Alzheimer’s Disease Bipolar II BP Longevity MDD Mood Disorders NOS Pain PTSD Risky Behavior SAD Social Isolation Stress Subsyndromal symptomatic depression Suicide SZ	Clozapine	19
**ADRA2A** Adrenoceptor Alpha 2A	209869_at	(I) AP/4 50%	4	9.07E-01/0 Not Stepwise	**Gender-dx** **M-MDD** **C:** (2/57) 0.88/3.43E-02	**Gender** **Females** **C:** (17/88) 0.63/4.91E-02 **F-SZ** **C:** (4/8) 0.88/4.07E-02 **L:** (3/5) 1/4.16E-02	**ALL** **C:** (18/236) 0.65/1.80E-02 **Gender** Males **C:** (15/199) 0.67/1.44E-02 **Gender-dx** **M-PSYCHOSIS** **C:** (7/74) 0.81/3.22E-03 **M-PTSD** **C:** (3/10) 0.86/4.37E-02 **M-SZ** **C:** (4/42) 0.91/3.47E-03	**ALL** **C:** (70/318) 1.41/4.65E-04 **Gender** Males **C:** (59/273) 1.44/5.74E-04 **Gender-dx** **M-PSYCHOSIS** **C:** (37/121) 1.52/3.60E-03 **M-PTSD** **C:** (5/12) 2.59/2.08E-02 **M-SZ** **C:** (14/63) 2.14/1.61E-04	Alcohol Bipolar COVID-19 Depression MDD Pain PTSD Suicide SZ	Carbamazepine Clozapine Norfluoxetine Valproate	17
**FZD10** Frizzled Class Receptor 10	219764_at	(I) DE/4 53.2%	2	3.57E-01/2 Stepwise		**ALL** **C:** (42/486) 0.6/1.39E-02 **L:** (21/291) 0.64/1.43E-02 **Gender** **Females** **C:** (17/88) 0.69/7.42E-03 **L:** (10/52) 0.68/3.88E-02 **F-BP** **C:** (4/31) 0.85/1.26E-02 **F-SZA** **C:** (1/20) 1/4.97E-02 **F-SZ** **L:** (3/5) 1/4.16E-02	**ALL** **C:** (18/236) 0.67/7.66E-03 **Gender** Males **C:** (15/199) 0.68/1.22E-02 **L:** (4/113) 0.75/4.52E-02 **Gender-dx** **M-PSYCHOSIS** **C:** (7/74) 0.75/1.64E-02 **L:** (1/39) 1/4.57E-02 **M-SZ** **C:** (4/42) 0.87/8.21E-03 **L:** (1/22) 1/4.90E-02	**ALL** **C:** (70/318) 1.34/3.63E-03 **Gender** Males **C:** (59/273) 1.34/6.61E-03 **Gender-dx** **M-PSYCHOSIS** **C:** (37/121) 1.55/2.70E-03 **M-SZ** **C:** (14/63) 1.92/5.16E-03	Alcohol Alzheimer’s BP Circadian abnormalities MDD PTSD	Aripiprazole Fluoxetine Gamma Frequency	17
**GRK4** G Protein-Coupled Receptor Kinase 4	210600_s_at	(I) DE/6 81.8%	0	3.40E-01/2 Stepwise	**Gender-dx** **M-MDD** **C:** (2/57) 0.87/3.77E-02	**Gender** **Females** **C:** (17/88) 0.64/3.26E-02 **Gender-dx** **F-BP** **C:** (4/31) 0.86/1.08E-02 **M-MDD** **L:** (4/31) 0.85/1.26E-02	**ALL** **C:** (18/236) 0.62/4.27E-02 **Gender** **Males** **C:** (15/199) 0.64/4.06E-02	**ALL** **C:** (70/318) 1.32/4.46E-03 **Gender** Males **C:** (59/273) 1.32/8.99E-03 **Gender-dx** F-MDD **C:** (2/15) 1.87/4.51E-02 **Gender-dx** **M-PSYCHOSIS** **C:** (37/121) 1.34/2.11E-02	Autism Bipolar Cannabis Depression PTSD Stress	Clozapine Gamma Frequency	17
**ATP1B2** ATPase Na+/K+ Transporting Subunit Beta 2	204311_at	(I) DE/2 39%	4	1.65E-01/2 Stepwise	**Gender-dx** **M-MDD** **C:** (2/57) 0.9/2.81E-02	**Gender** **Females** **C:** (17/88) 0.64/3.75E-02 **Gender-dx** **F-BP** **C:** (4/31) 0.84/1.46E-02 **F-PSYCHOSIS** **C:** (5/28) 0.77/3.37E-02	**Gender** Males **C:** (15/199) 0.63/4.18E-02 **Gender-dx** M-**PSYCHOSIS** **C:** (7/74) 0.79/5.55E-03 **M-PTSD** **C:** (3/10) 0.86/4.37E-02 **M-SZ** **C:** (4/42) 0.86/9.22E-03	**ALL** **C:** (70/318) 1.59/1.91E-05 **Gender** Females **C:** (11/45) 1.68/2.97E-02 Males **C:** (59/273) 1.56/1.33E-04 **Gender-dx** **F-MDD** **C:** (2/15) 3.16/3.10E-02 **M-BP** **C:** (13/96) 1.61/1.79E-02 **M-PSYCHOSIS** **C:** (37/121) 1.98/4.84E-05 **M-SZ** **C:** (14/63) 2.39/7.75E-04 **M-SZA** **C:** (23/58) 1.68/1.24E-02	Alcohol Alzheimer’s MDD PTSD Stress Substance Abuse Suicide SZ	Acetyldigitoxin Clozapine Deslanoside Digitoxin Digoxin Haloperidol Lithium Valproate	16
**CLIC6** Chloride Intracellular Channel 6	242913_at	(I) AP/6 84.2%	2	5.06E-01/0 Not Stepwise		**Gender** **Females** **C:** (17/88) 0.67/1.68E-02 **Gender-dx** **F-BP** **C:** (4/31) 0.8/2.97E-02 **F-PSYCHOSIS** **C:** (5/28) 0.85/8.20E-03 **F-SZ** **C:** (4/8) 0.97/1.47E-02 **F-SZA** **C:** (1/20) 1/4.97E-02	**ALL** **C:** (18/236) 0.62/4.11E-02 **Gender-dx** **M-PSYCHOSIS** **C:** (7/74) 0.71/3.17E-02 **Gender-dx** **M-SZ** **C:** (4/42) 0.8/2.44E-02	**ALL** **C:** (70/318) 1.32/6.89E-03 **Gender** Females **C:** (11/45) 1.59/4.32E-02 Males **C:** (59/273) 1.27/2.51E-02 **Gender-dx** M-SZ **C:** (14/63) 1.68/1.04E-02	Aging Alcohol Cocaine MDD Phencyclidine Restraint Stress Suicide	Clozapine Omega-3 fatty acids Gamma Frequency	16
**EFNA5** Ephrin A5	1559360_at	(I) DE/6 80.5% (I) AP/4 51.3%	2	2.02E-04/4 Nominal		**Gender** **Females** **C:** (17/88) 0.63/4.85E-02 **Gender-dx** **M-SZ** **C:** (6/90) 0.71/4.03E-02	**ALL** **L:** (5/133) 0.74/3.34E-02 **Gender** Males **L:** (4/113) 0.75/4.67E-02 **Gender-dx** **F-SZA** **C:** (2/6) 1/3.20E-02 **M-SZA** **C:** (3/32) 0.79/4.96E-02	**Gender-dx** **M-BP** **C:** (13/96) 1.41/2.92E-02	Alcohol Alzheimer’s BP Cannabis Chronic Fatigue Syndrome Depression Insomnia Intellect Longevity MDD Morphine Pain Stress Suicide SZ	Omega-3 fatty acids Gamma Frequency	18
**GPX7** Glutathione Peroxidase 7	213170_at	(D) DE/4 64.3%	2	4.64E-01/2 Stepwise	**ALL** **C:** (19/495) 0.63/2.63E-02	**Gender-dx** **F-BP** **C:** (4/31) 0.86/1.08E-02 **F-SZA** **C:** (1/20) 1/4.97E-02 **M-MDD** **L:** (4/31) 0.86/1.08E-02	**Gender-dx** **M-PSYCHOSIS** **C:** (7/74) 0.71/3.17E-02 **M-SZ** **C:** (4/42) 0.82/1.99E-02	**ALL** **C:** (70/318) 1.42/6.50E-03 **Gender** **Males** **C:** (59/273) 1.47/7.12E-03 **Gender-dx** **M-PSYCHOSIS** **C:** (37/121) 1.5/2.40E-02 **M-SZ** **C:** (14/63) 1.9/4.95E-02	Aging MDD Neuropathic pain SZA	Mianserin S-adenosyl methionine (SAM)	16
**NTRK3** Neurotrophic Receptor Tyrosine Kinase 3	215025_at	(I) DE/2 35.1%	4	6.58E-01/2 Stepwise	**Gender-dx** **M-BP** **L:** (2/93) 0.9/2.83E-02	**ALL** **C:** (42/486) 0.61/9.05E-03 **L:** (21/291) 0.64/1.43E-02 **Gender** **Females** **C:** (17/88) 0.72/2.51E-03 **Males** **L:** (11/239) 0.67/2.55E-02 **Gender-dx** **F-BP** **C:** (4/31) 0.86/1.08E-02 **F-PSYCHOSIS** **C:** (5/28) 0.77/2.93E-02 **F-SZ** **C:** (4/8) 0.94/1.92E-02 **M-BP** **L:** (2/92) 0.93/1.84E-02	**Gender-dx** **M-PSYCHOSIS** **C:** (7/74) 0.7/4.55E-02 **Gender-dx** **M-SZ** **C:** (4/42) 0.82/1.99E-02 **L:** (1/22) 1/4.90E-02	**ALL** **C:** (70/318) 1.36/5.70E-03 **Gender** Males **C:** (59/273) 1.4/4.76E-03 **Gender-dx** **M-BP** **C:** (13/96) 1.95/1.24E-02 **M-PSYCHOSIS** **C:** (37/121) 1.4/1.87E-02 **M-SZ** **C:** (14/63) 1.93/3.78E-03	Aging Alcohol Alzheimer’s Disease Bipolar I BP Depression Longevity MDD Mood Disorders NOS Pain PTSD Risky Behavior SAD Social Isolation Stress Subsyndromal symptomatic depression Suicide SZ	Clozapine	16
**SLC6A2** Solute Carrier Family 6 Member 2	217214_s_at	(I) DE/2 40.3%	4	3.40E-01/2 Stepwise	**Gender-dx** **F-PSYCHOSIS** **L:** (4/17) 0.81/3.50E-02 **F-SZA** **L:** (4/12) 0.81/4.47E-02	**ALL** **C:** (42/486) 0.63/2.37E-03 **L:** (21/291) 0.62/3.78E-02 **Gender** **Females** **C:** (17/88) 0.76/4.78E-04 **Gender-dx** **F-MDD** **C:** (7/21) 0.77/2.62E-02 **F-PSYCHOSIS** **C:** (5/28) 0.8/1.92E-02 **F-SZ** **C:** (4/8) 0.94/2.17E-02 **M-SZ** **C:** (6/90) 0.71/4.63E-02	**Gender-dx** M-PSYCHOSIS **C:** (7/74) 0.72/2.92E-02 M-SZA **C:** (3/32) 0.87/1.78E-02 M-PSYCHOSIS **L:** (1/39) 1/4.57E-02 M-SZ **L:** (1/22) 1/4.90E-02	**ALL** **C:** (70/318) 1.33/4.74E-03 **Gender** Males **C:** (59/273) 1.34/6.73E-03 **Gender-dx** M-BP **C:** (13/96) 1.61/4.50E-02 M-PSYCHOSIS **C:** (37/121) 1.31/2.54E-02	Alcohol Depression MDD Postpartum Depression PTSD Suicide	Fluoxetine Norfluoxetine	16
**SLC6A4** Solute Carrier Family 6 Member 4	242009_at	(I) DE/2 35.1%	10	8.51E-01/0 Not Stepwise			**Gender-dx** **F-PSYCHOSIS** **C:** (2/13) 0.91/3.76E-02 **F-SZA** **C:** (2/6) 1/3.20E-02 **M-PTSD** **C:** (3/10) 0.86/4.37E-02 **M-SZ** **C:** (4/42) 0.76/4.74E-02	**ALL** **C:** (70/318) 1.21/1.15E-02 **Gender** Males **C:** (59/273) 1.19/2.77E-02 **Gender-dx** **M-PTSD** **C:** (5/12) 2.54/3.24E-02	Aging Alcohol Anxiety BPD Depression Early Life Stress MDD MSK Neuropathic pain Pain Postpartum Depression PTSD Stress Suicide	Agomelatine Benzodiazepines Citalopram Clozapine Omega-3 fatty acids Oxycodone Sertraline Vortioxetine	16
**TMEM138** Transmembrane Protein 138	223113_at	(D) DE/4 63%	4	8.63E-01/0 Not Stepwise		**Gender** Males L: (11/239) 0.66/4.12E-02 **Gender-dx** F-BP C: (4/31) 0.86/1.08E-02	**ALL** **L:** (5/129) 0.72/4.61E-02 **Gender-dx** M-PTSD **C:** (3/10) 0.86/4.37E-02	**ALL** **C:** (70/318) 1.3/1.46E-02 **Gender** Males **C:** (59/273) 1.34/1.20E-02 **Gender-dx** M-PSYCHOSIS **C:** (37/121) 1.5/6.57E-03 M-PTSD **C:** (5/12) 3.71/4.13E-02 M-SZA **C:** (23/58) 1.51/2.83E-02	Alcohol Brain arousal Depression	Antidepressants	16
**ANKRD28** Ankyrin Repeat Domain 28	229307_at	(I) DE/6 80.5%	0	6.20E-01/2 Stepwise		**ALL** **C:** (42/486) 0.62/5.30E-03 **Gender** **Males** **C:** (25/398) 0.72/1.31E-04 **Gender-dx** **M-MDD** C: (7/57) 0.89/3.97E-04 **Gender-dx** **M-PSYCHOSIS** **C:** (12/165) 0.72/5.72E-03 **Gender-dx** **M-SZ** **C:** (6/90) 0.84/3.13E-03	**Gender-dx** **F-PSYCHOSIS** **C:** (2/13) 0.91/3.76E-02 **Gender-dx** **F-SZA** **C:** (2/6) 1/3.20E-02	**ALL** **C:** (70/318) 1.22/2.96E-02 **Gender-dx** F-MDD **C:** (2/15) 3.09/3.59E-02 **Gender** Males **C:** (59/273) 1.21/4.06E-02 **Gender-dx** **M-PSYCHOSIS** **C:** (37/121) 1.42/7.30E-03 **Gender-dx** **M-SZA** **C:** (23/58) 1.61/1.24E-03	Alcohol ASD BP Childhood Trauma Depression MDD Mood instability PTSD Stress Suicide	N/A	15
**CCKBR** Cholecystokinin B Receptor	210381_s_at	(I) DE/2 48%	4	4.37E-01/2 Stepwise		**Gender-dx** **F-BP** **C:** (4/31) 0.8/2.97E-02 **Gender-dx** **M-BP** **C:** (5/139) 0.74/3.70E-02 **Gender-dx** **F-BP** **L:** (3/18) 0.82/4.29E-02	**ALL** **C:** (18/236) 0.63/3.01E-02 **Gender-dx** **F-PSYCHOSIS** **C:** (2/13) 0.91/3.78E-02 **Gender-dx** **F-SZA** **C:** (2/6) 1/3.20E-02 **Gender** Males **C:** (15/199) 0.63/4.48E-02 **Gender-dx** M-PTSD **C:** (3/10) 0.86/4.37E-02 **Gender-dx** M-SZ **C:** (4/42) 0.76/4.74E-02	**ALL** **C:** (70/318) 1.33/4.28E-03 **Gender** Males **C:** (59/273) 1.32/9.50E-03 **Gender-dx** M-PSYCHOSIS **C:** (37/121) 1.3/3.14E-02	Alcohol BP Chronic Stress MDD Phencyclidine Suicide SZ	Clozapine	15
**DYNLL2** Dynein Light Chain LC8-Type 2	229106_at	(D) DE/2 39.3%	4	1.96E-01/2 Stepwise	**Gender** **Males** **L:** (4/246) 0.78/2.79E-02 **Gender-dx** **M-BP** **L: (**2/93) 0.85/4.50E-02	**Gender-dx** F-SZA **C:** (1/20) 1/4.97E-02	**ALL** **L:** (5/129) 0.74/3.36E-02	**Gender-dx** M-SZA **C:** (23/58) 1.49/4.09E-02	Aging Alcohol Alzheimer’s Disease Methamphetamine Stress SZ	Benzodiazepines Valproate	15
**Hs.550187**	240253_at	(I) DE/6 88.3%	0	2.74E-01/2 Stepwise		**Gender** **Females** **C:** (17/88) 0.72/2.59E-02 **Gender-dx** **F-BP** **C:** (4/31) .84/1.46E-02	**Gender** **Females** **C:** (3/37) 0.8/4.23E-02 **Gender-dx** **F-SZA** **C:** (2/6) 1/3.20E-02 **M-PSYCHOSIS** **C:** (7/74) 0.71/3.44E-02 **M-SZA** **C:** (3/32) 0.9/1.29E-02	**ALL** **C:** (70/318) 1.19/3.48E-02 **Gender-dx** **M-PSYCHOSIS** **C:** (37/121) 1.23/2.54E-02 **M-SZ** **C:** (14/63) 1.27/3.90E-02	Suicide	N/A	15
**NRG1** Neuregulin 1	208232_x_at	(I) DE/4 65.8%	4	6.66E-01/2 Stepwise	**Gender-dx** **M-MDD** **C:** (2/57) 0.93/2.08E-02 **M-BP** **L:** (2/93) 0.85/4.50E-02	**Gender-dx** **F-BP** **C:** (4/31) 0.81/2.59E-02 **Gender-dx** **M-PSYCHOSIS** **C:** (12/165) 0.73/3.69E-03 **M-SZ** **C:** (6/90) 0.7/4.79E-02 **M-SZA** **C:** (6/75) 0.76/1.66E-02		**ALL** **C:** (70/318) 1.2/3.99E-02 **Gender** Males **C:** (59/273) 1.27/1.37E-02 **Gender-dx** **M-BP** **C:** (13/96) 1.66/1.80E-02 **M-PSYCHOSIS** **C:** (37/121) 1.25/4.26E-02 **M-SZA** **C:** (23/58) 1.32/4.07E-02	Aging Alcohol Alzheimer’s BP Chronic Stress Cocaine COVID-19 Depression Longevity MDD Memory Methamphetamine Psychosis PTSD Suicide SZ	Ketamine Antipsychotics Valproate Lithium	15
**TFRC** Transferrin Receptor	207332_s_at	(D) DE/4 56%	2.00	7.11E-01/2 Stepwise	**Gender** **Females** **L:** (4/52) 0.82/1.66E-02 **Gender-dx** **F-PSYCHOSIS** **C:** (4/28) 0.77/4.39E-02 **L:** (4/17) 0.85/2.08E-02 **F-SZA** **L:** (4/12) 0.84/3.09E-02	**Gender-dx** **F-BP** **C:** (4/31) 0.88/7.85E-03	**Gender-dx** M-PTSD **C:** (3/10) 0.9/2.64E-02	**ALL** **C:** (70/318) 1.29/2.21E-02 **Gender** Males **C:** (59/273) 1.35/1.46E-02 **Gender-dx** M-SZA **C:** (23/58) 1.54/3.73E-02	Aging Alcohol Alzheimer’s BP Cocaine Depression Longevity MDD (Recurrent) Neuropathic Pain SZ	Valproate	15

After Step 4 Testing in independent cohorts for state and trait predictive ability. For Step 4 Predictions, **C:** -cross-sectional (using levels from one visit), **L:** -longitudinal (using levels and slopes from multiple visits). In ALL, by Gender, and personalized by Gender and Diagnosis (Gender/Dx). M-Males, F-Females. MDD-depression, BP-bipolar, SZ-schizophrenia, SZA-schizoaffective, PSYCHOSIS- schizophrenia and schizoaffective combined, PTSD-post-traumatic stress disorder.

### Biological understanding

#### Biological pathways

We carried out biological pathway analyses using the list of top candidate biomarkers for anxiety (*n* = 82 genes, 95 probesets), which suggests that Hippo signaling pathway and CREB signaling pathway are involved (Table 2). Depression, alcohol consumption, and attention deficit disorder/conduct disorder/oppositional defiant disorder were top diseases identified by the pathway analyses using DAVID, pointing out the issue of co-morbidity, and Ingenuity identified neurological disorders, organismal injury, and cancer as the top medical co-morbidities.

**Table 2:** Biology of Anxiety Biomarkers. Top CFE3 ≥8 (*n* = 95 probesets, 82 genes).

A.	KEGG Pathways				Ingenuity Pathways		
	Term	Count	%	*P* Value	Top Canonical Pathways	*P* Value	Overlap
**Top candidate biomarkers** (*n* = 95 probesets, 82 genes)	**Hippo Signaling Pathway**	6	7.4	1.60E-03	**CREB Signaling in Neurons**	4.56E-06	1.8 % 11/606
	Neuroactive Ligand-receptor Interaction	8	9.9	3.40E-03	Cardiac Hypertrophy Signaling (Enhanced)	1.12E-05	1.8 % 10/542
	Proteoglycans in Cancer	6	7.4	5.20E-03	Relaxin Signaling	1.23E-05	3.9 % 6/155
	Rap1 Signaling Pathway	6	7.4	5.70E-03	Molecular Mechanisms of Cancer	1.57E-05	2.0 % 9/446
	**cAMP Signaling Pathway**	6	7.4	7.10E-03	G-Protein Coupled Receptor Signaling	1.81E-05	1.6 % 11/702

B.	David					Ingenuity Pathways Disease		
**Top candidate biomarkers** (*n* = 95 probesets, 82 genes)	#	**Term**	**Count**	**%**	***P*** **Value**	**Diseases and Disorders**	***P*** **Value**	**# Molecules**
	1	Depression	11	13.6	5.90E-09	Neurological Disease	2.43E-04–7.96E-12	65
	2	Several Psychiatric disorders	14	17.3	6.10E-09	Organismal Injury and Abnormalities	2.46E-04–7.96E-12	77
	3	Alcohol consumption	10	12.3	3.10E-08	Cancer	2.45E-04–2.78E-10	77
	4	Attention deficit disorder Conduct disorder Oppositional defiant disorder	7	8.6	6.60E-07	Hematological Disease	2.24E-04–2.78E-10	45
	5	Tourette Syndrome	5	6.2	2.30E-06	Immunological Disease	1.64E-04–2.78E-10	49
	6	Panic Disorder	6	7.4	5.20E-06			
	7	Bulimia	9	11.1	6.20E-06			
	8	Schizophrenia	14	17.3	8.40E-06			
	9	Tobacco Use Disorder	34	42	1.20E-05			
	10	Bipolar Disorder	8	9.9	1.30E-05			

C.	
Co-morbidity	Percentile Match
Depression	83.33
Alcoholism	72.22
Stress	55.56
Schizophrenia	50.00
Bipolar	44.44
Aging	38.89
Dementia	38.89
PTSD	38.89
Suicide	38.89
Pain	27.78
Phencyclidine	27.78
Cocaine	16.67
Cannabis	11.11
Mood	11.11
ASD	5.56
Fear	5.56
Memory	5.56
Methamphetamine	5.56
Morphine	5.56
Neurological	5.56
Schizoaffective	5.56

A Pathway Analyses, B Diseases, C Psychiatric co-morbidities. **C** Genomic co-morbidity for Anxiety for Top Biomarkers from Table 1 (*n* = 19). See also table S3.

#### Networks and interactions

We carried out a STRING analysis (Fig. S4) of the top candidate biomarkers that revealed groups of interacting proteins. In particular, HTR2A is at the overlap of a network containing GAD1, GABBR1, and SLC6A4 (the serotonin transporter), and one centered on PIK3R1 that also contains CCKBR and IGFR1. A third network includes DLGAP1, DYNLL2, and PTPRD. These networks may have biological significance and could be targeted therapeutically. The first network may have to do with reactivity, and contains genes that are targeted by the current standard treatments for anxiety, namely serotonin-reuptake inhibitors and benzodiazepines. The second network may have to do with activity, and contains genes that are involved in neurotrophic functions. The third network may have to do with connectivity, and contains genes that are involved in synaptic structure and function.

### Testing for clinical utility

In Step 4 Testing, we examined in independent cohorts from the ones used for discovery or validation whether the 95 top candidate biomarkers can assess high anxiety state (*n* = 197 subjects with 495 visits), clinical anxiety state (*n* = 195 subjects with 486 visits), as well as predict of future psychiatric hospitalizations due to anxiety (*n* = 130 subjects with 318 visits) (Fig. 2, and Table 1), using electronic medical records follow-up data of our study subjects (up to 14.74 years from initial visit at the time of the analyses) (Fig. 1, Table S1). The gene expression data in the test cohorts was normalized (Z-scored) across genders and various psychiatric diagnoses, before those different demographic groups were combined. We used as predictors biomarker levels information cross-sectionally, as well as expanded longitudinal information about biomarker levels at multiple visits. We tested the biomarkers in all subjects in the independent test cohort, as well as in a more personalized fashion by gender and psychiatric diagnosis.

For high anxiety state assessment across all subjects in the independent test cohort, the best biomarker was ERCC6L2, decreased in expression in high anxiety, with an AUC of 68 % (*p* = 0.004) cross-sectionally, and an AUC of 69% (*p* = 0.03) longitudinally (Fig. 2A). It also has an AUC of 72% (*p* = 0.003) cross-sectionally in males, and an AUC of 76% (*p* = 0.02) cross-sectionally in male bipolars. ERCC6L2 also has an AUC of 100% (*p* = 0.0007) longitudinally for clinical anxiety in males with depression. ERCC6L2 (ERCC Excision Repair 6 Like 2) is a novel gene for anxiety disorders, with no prior evidence of involvement in the literature. ERCC6L2 is a member of the Snf2 family of helicase-like proteins. The encoded protein may play a role in mitochondrial function and DNA repair. Reactivity and repair may be key functions of anxiety [8].

For assessment of clinical anxiety state in the independent test cohort, SLC6A2, increased in expression in high anxiety in our work, had an AUC of 63% (*p* = 0.02) across all subjects, and 76% (*p* = 0.0005) in females, surviving Bonferroni correction for all 95 biomarkers tested. It also had a Cox regression Odds Ratio (OR) of 9.02 (*p* = 0.0004) for predicting all future hospitalizations for anxiety in males with schizophrenia, being Bonferroni significant. SLC6A2 (Solute Carrier Family 6 Member 2) is the norepinephrine transporter. Medications that block this transporter by itself, or in conjunction with blocking SLC6A4 (Solute Carrier Family 6 Member 4, the serotonin transporter), another one of our findings, also increased in expression in high anxiety, have been shown to be useful clinically in anxiety disorders [9].

SLC6A4 is an example of a previously well-known gene reproduced in this study, albeit with weaker evidence. For all future hospitalizations with anxiety in the independent test cohort SLC6A4, increased in expression in high anxiety, had an OR of 1.21 (*p* = 0.01) across all subjects. It had an OR of 2.54 (*p* = 0.03) in PTSD. The product of this gene is the serotonin transporter, which is the target of serotonin reuptake inhibitors used to treat stress disorders, anxiety, as well as depression, conditions that are highly related and co-morbid.

We also tested a panel of the 95 candidate biomarkers, which showed synergistic benefits (better than any individual biomarker) for predicting trait. The panel (BioM-95) was the best predictor of future hospitalizations with anxiety in all patients (Cox regression OR of 2.42, *p* = 0.02), and an even better predictor in males (OR 2.69, *p* = 0.015), and in males with psychosis (OR of 3.36, *p* = 0.016). Interestingly, this was also better than a standard clinical measure, STAI Trait, performed (OR 1.4, *p* = 0.043).

### Convergent Functional Evidence (CFE)

For the top candidate biomarkers (*n* = 95), we computed into a CFE score all the evidence from discovery (up to 6 points), CFG prioritization (up to 12 points), validation (up to 6 points), and testing (state high anxiety, state clinical anxiety, trait first-year hospitalization with anxiety, trait all future hospitalizations with anxiety- up to 3 points each if it significantly predicts in all subjects, 2 points if in gender, 1 points if in gender/diagnosis). The total score can be up to 36 points: 24 from our own new data, and 12 from literature data used for CFG. We weigh our new data more than the literature data, as it is functionally related to anxiety in three independent cohorts (discovery, validation, testing). The goal is to highlight, based on the totality of our data and of the evidence in the field to date, biomarkers that have all around evidence: track anxiety, have convergent evidence for involvement in anxiety disorders, and predict anxiety state and future clinical events (Table 1).

The top blood biomarkers (*n* = 19 probesets, in 18 genes) with the strongest overall CFE for tracking and predicting anxiety disorders, after all four steps (Table 1) were, in order of CFE4 score: GAD1 (Glutamate Decarboxylase 1), NTRK3 (Neurotrophic Receptor Tyrosine Kinase 3), ADRA2A (Adrenoceptor Alpha 2A), FZD10 (Frizzled Class Receptor 10), GRK4 (G Protein-Coupled Receptor Kinase 4), ATP1B2 (ATPase Na+/K + Transporting Subunit Beta 2), CLIC6 (Chloride Intracellular Channel 6), EFNA5 (Ephrin A5), GPX7 (Glutathione Peroxidase 7), again NTRK3 (Neurotrophic Receptor Tyrosine Kinase 3), SLC6A2 (Solute Carrier Family 6 Member 2), SLC6A4 (Solute Carrier Family 6 Member 4), TMEM138 (Transmembrane Protein 138), ANKRD28 (Ankyrin Repeat Domain 28), CCKBR (Cholecystokinin B Receptor), DYNLL2 (Dynein Light Chain LC8-Type 2), Hs.550187, NRG1 (Neuregulin 1), and TFRC (Transferrin Receptor).

GAD1 (Glutamate Decarboxylase 1), the overall top biomarker for anxiety in this study, synthesizes gamma-aminobutyric acid (GABA) from glutamate. Abnormalities in the GABA neurotransmitter system have been noted in subjects with mood and anxiety disorders. GAD1 has previous genetic evidence in anxiety and panic disorders [10]. It is increased in expression in blood in high anxiety in our work. The gene had been previously described to be hypomethylated in panic disorders patients, which is consistent with higher expression of the gene [11, 12]. GAD1 in our studies modestly predicts clinically severe anxiety state in all patients in the independent testing cohort (AUC 58%, *p* = 0.04), with results being somewhat better in women (AUC 65%, *p* = 0.03). It also predicts future hospitalizations with anxiety in all (OR 1.3, *p* = 0.005).

### Therapeutics

#### Pharmacogenomics

Only one of the top biomarkers, DYNLL2, has evidence for being modulated by benzodiazepines in the opposite direction to that in high anxiety; the others do not, which is interesting and clinically useful, as it brings to the fore other, non-addictive, choices.

Overall, based on number of biomarkers modulated in expression in opposite direction to anxiety, valproate (33%) had the best evidence for broad efficacy in anxiety disorders (Table 3A), followed by omega- 3 fatty acids (28%). Another alternative treatment that was a top match was EEG gamma band frequency (17%), which is increased by meditation and other mindfulness practices. Lithium (11%) and fluoxetine (11%) were next, and benzodiazepines (6%) were a lower match than that. Omega-3 fatty acids and meditation may be a widely deployable preventive treatment, with minimal side-effects, including in women who are or may become pregnant.

**Table 3:** Therapeutics.

A	
Treatment	Percentile Match
Valproate	33.33
Clozapine	33.33
Omega-3 fatty acids	27.78
Gamma frequency	16.67
Fluoxetine	11.11
Lithium	11.11
Carbamazepine	11.11
Agomelatine	5.56
Benzodiazepines	5.56
Haloperidol	5.56
Imipramine	5.56
Ketamine	5.56
Mianserin	5.56
S-adenosyl methionine (SAM)	5.56
Sertraline	5.56
Vortioxetine	5.56

B			
Connectivity Map (CMAP) analyses			
rank	cmap name	score	Roles
1	thalidomide	−1	Originally introduced as a non-barbiturate hypnotic but withdrawn from the market due to teratogenic effects. It has been reintroduced and used for a number of immunological and inflammatory disorders.
2	ethoxyquin	−0.984	Ethoxyquin is a genotoxic quinoline.
3	estradiol	−0.979	Female sex hormone
4	tetracaine	−0.96	Local anesthetic
5	pirenperone	−0.957	5-HT2A receptor antagonist described as an antipsychotic and tranquilizer which was never marketed.
6	atropine	−0.953	Muscarinic antagonist
7	15(S)-15-methylprostaglandin E2	−0.933	Labor induction
8	loperamide	−0.919	Peripheral opioid receptor agonist used to treat diarrhea
9	tropicamide	−0.918	Muscarinic antagonist
10	esculetin	−0.917	Plant toxin
11	isocorydine	−0.916	Alkaloid
12	disopyramide	−0.913	Antiarrhythmic

**A** Best existing treatments for Anxiety By matching to Top Biomarkers from Table 1 (*n* = 19). See also table S4. **B.** Drug repurposing for Anxiety using Connectivity Map [28] (CMAP) For Top Biomarkers from Table 1 (*n* = 19). Direction of expression in high anxiety. 2 out of 4 Decreased and 10 out of 15 Increased probesets were present in HG-U133A, the array used for CMAP (from genes ADRA2A, ATP1B2, CCKBR, FZD10, GAD1, GPX7, GRK4, NRG1, NTRK3, SLC6A2, TFRC). Drugs that have *opposite* gene expression effects to the gene expression signature.

A number of individual top biomarkers are known to be modulated by medications in current clinical use for treating affective disorders and suicidality, such as lithium (GAD1, ATP1B2, NRG1), the nutraceutical omega-3 fatty acids(GAD1, CLIC6, EFNA5, SLC6A4), and antidepressants (ADRA2A, FZD10, GPX7, SLC6A2, SLC6A4, TMEM138) (Tables 1 and S4). This is of potential utility in pharmacogenomics approaches matching anxious and suicidal patients to the right medications, and monitoring response to treatment.

#### New drug discovery/repurposing

Bioinformatic analyses using the gene expression signature of the panel of top biomarkers for high anxiety (Table 3B) identified new potential therapeutics for anxiety, such as the female sex hormone estradiol, the 5-HT2A receptor antagonist pirenperone, the peripheral opioid receptor agonist loperamide, and the antiarrhythmic disopyramide. Interestingly, ESR1 (estrogen receptor 1) was one of the top genetic findings in a recent independent GWAS study [13].

## Discussion

We describe a novel and comprehensive effort to discover and validate blood biomarkers of relevance to anxiety, including testing them in independent cohorts to evaluate predictive ability and clinical utility. These biomarkers also open a window into understanding the biology of anxiety disorders, as well as indicate new and more precise therapeutic approaches.

### Current clinical practice and the need for biomarkers

Assessing a person’s internal subjective feelings and thoughts, along with more objective external ratings of actions and behaviors, are used in clinical practice to assess anxiety and diagnose clinical anxiety disorders, such as panic attacks and generalized anxiety disorder. Such an approach is insufficient, and lagging those used in other medical specialties. Moreover, there is a delay with a range of 9 to 23 years between illness onset and diagnosis [14]. Blood biomarkers related to anxiety would provide a critical objective measurement to inform clinical assessments and treatment decisions.

### Advantages of biomarkers

Blood biomarkers offer real-world clinical practice advantages. As the brain cannot be readily biopsied in live individuals, and CSF is less easily accessible than blood, we have endeavored over the years to identify blood biomarkers for neuropsychiatric disorders. A whole-blood approach facilities field deployment of sample collection. The assessment of gene expression changes focuses on our approach on immune cells. The ability to identify peripheral gene expression changes that reflect brain activities is likely due to the fact that the brain and immune system have developmental commonalities, marked by shared reactivity and ensuing gene expression patterns. There is also a bi-directional interaction between the brain and immune system. Not all changes in expression in peripheral cells are reflective of or germane to brain activity. By carefully tracking a phenotype with our within-subject design in the discovery step, and then using convergent functional genomics prioritization, we are able to extract the peripheral changes that do track and are relevant to the brain activity studied, in this case anxiety state, and its disorders.

Subsequent validation and testing in independent cohorts narrow the list to the best markers. In the end, we do not expect to recapitulate in the blood all that happens in the brain. We just want to have good accessible peripheral biomarkers—“liquid biopsies”, as they are called in cancer.

### Comprehensiveness

In this current work, we carried out extensive blood gene expression studies in male and female subjects with major psychiatric disorders, an enriched population in terms of co-morbidity with anxiety disorders and variability of anxiety. In fact, besides their primary clinical diagnosis, overall, over 20% of the subjects in our study had a co-morbid clinical anxiety disorders diagnosis, the highest percentage (37.3%) being those with major depressive disorder (MDD) as their primary diagnosis (Table S1B). The potential molecular-level co-morbidity between other psychiatric disorders and anxiety disorders is underlined by the fact that medications for other disorders (antidepressants, mood stabilizers, even antipsychotics) are also used to treat PTSD and anxiety disorders. Our primary goal was to discover and validate biomarkers for anxiety, that are transdiagnostic. Secondarily, we aimed to understand their universality vs. their specificity by gender and psychiatric diagnosis.

Our studies were arranged in a step-wise fashion. First, we endeavored to discover blood gene expression biomarkers for anxiety using a longitudinal design, looking at differential expression of genes in the blood of male and female subjects with major psychiatric disorders (bipolar disorder, major depressive disorder, schizophrenia/schizoaffective, and post-traumatic stress disorder (PTSD)), high-risk populations prone to anxiety, which constitute an enriched pool in which to look for biomarkers. We compared low anxiety states to high anxiety states using a powerful within-subject design [4–6, 15], to generate a list of differentially expressed genes. Second, we used a comprehensive Convergent Functional Genomics (CFG) approach with the whole body of knowledge in the field to prioritize from the list of differentially expressed genes/biomarkers of relevance to anxiety. CFG integrates multiple independent lines of evidence- genetic, gene expression, and protein data, from brain and periphery, from human and animal model studies, as a Bayesian strategy for identifying and prioritizing findings, reducing the false-positives and false-negatives inherent in each individual approach. Third, we examined if the expression levels of the top biomarkers identified by us as tracking anxiety state are changed even more strongly in blood samples from an independent cohort of subjects with clinically severe anxiety, to validate these biomarkers. Fourth, the biomarkers thus discovered, prioritized, and validated were tested in corresponding independent cohorts of psychiatric subjects. Fifth, we used the biomarkers to match to existing psychiatric medications, as well as to identify and potentially repurpose new drugs for anxiety disorders treatment using bioinformatics analyses. The series of studies was a systematic and comprehensive approach to move the field forward towards precision medicine.

### Power

We used a systematic discovery, prioritization, validation, and testing approach, as we have done over the years for other disorders [2, 7, 16–18]. For discovery, we used a hard to accomplish but powerful within-subject design, with an *N* of 58 subjects with 149 visits. A within-subject design factors out genetic variability, as well as some medications, lifestyle, and demographic effects on gene expression, permitting identification of relevant signal with *N*s as small as 1^15^. Another benefit of a within-subject design may be accuracy/consistency of self-report of psychiatric symptoms (“phene expression”), as it is the same person reporting different states. This is similar in rationale to the signal detection benefits it provides in gene expression.

Based on our work of over two decades in genetics and gene expression, along with the results of others in the field, we estimate that using a quantitative phenotype is up to 1 order of magnitude more powerful than using a categorical diagnosis. The within-subject longitudinal design, by factoring out all genetic and some environmental variability, is up to 3 orders of magnitude more powerful than an inter-subject case-control cross-sectional design. Moreover, gene expression, by integrating the effects of many SNPs and environment, is up to 3 orders of magnitude more powerful than a genetic study. Combined, our approach may be up to 6 orders of magnitude more powerful than a GWAS study, even prior to the CFG literature-based prioritization step, which encompasses all the independent work in the field prior to our studies, which may add up to 1 order of magnitude as well. In addition, the Validation and the Testing steps add additional 1 order of magnitude power each. As such, our approach may be up to 10 orders of magnitude more powered to detect signal than most current genetic study designs as used in GWAS.

### Reproducibility

We reproduced and expanded our earlier findings in an animal model of GABBR1, CCKBR, and DYNLL2 [8] as top genes involved in anxiety.

Additionally, there is reproducibility with findings generated by other independent large-scale studies that came out after our analyses were completed, and were thus not included in our CFG approach. A number of their top findings were present in our candidate gene expression biomarkers for anxiety list that had survived our initial whole-genome, unbiased, within-subject Discovery step, before any CFG literature prioritization: two out of their ten top genes for lifetime anxiety disorder in a UK Biobank study [19], 6 out of 11 top genes for anxiety in a Million Veteran’s Program study [13] and 13 out of 42 top blood biomarkers in a study of intergenerational trauma [20] (see Supplementary Information- Reproducibility file). This independent reproducibility of findings between our studies and these other genetic and gene expression studies, which are done in independent cohorts from ours, with independent methodologies, is reassuring, and provides strong convergent evidence for the validity and relevance of our approach and of their approaches. Our work also provides functional evidence for some of their top genetic hits.

### Pathophysiology

A number of top candidate biomarkers identified by us have biological roles that are related to signaling, in particular decreased cAMP and CREB signaling (Table 2). This reproduces one of the main conclusions we had in animal model studies of anxiety over a decade ago [8]. Decreased cAMP signaling has also been reported in ADHD, autism spectrum disorders and fragile X syndrome. This provides a molecular underpinning for the epidemiological data between anxiety and disrupted attention and memory, and for the clinical co-morbidity between these disorders. It also suggests that drugs that increase cAMP activity, such as stimulants [21], as well as lithium, glutamate antagonists such as magnesium, and PDE-4 inhibitors such as rolipram [22], may be helpful in anxiety disorders. The results to date in clinical trials of these agents have been mixed, which points to the issue of heterogeneity in the population and the need to use a personalized, biomarker-driven approach. Another top pathway is the Hippo signaling pathway, which has been implicated in stress-related psychiatric disorders [23].

The majority of top blood biomarkers we have identified have prior evidence in human or animal model brain data from anxiety disorders studies, which indicates their relevance to the pathophysiology of anxiety disorders (Table S2). The co-directionality of blood changes in our work and brain changes reported in the literature needs to be interpreted with caution, as it may depend on brain region.

The top candidate biomarkers also had prior evidence of involvement in other psychiatric and related disorders (Table S3), providing a molecular basis for co-morbidity and the possible precursor effects of some these disorders on anxiety, and conversely, the precursor role of anxiety in some of them. In particular, a majority of them have an overlap with depression (83%), as well as alcoholism (72%), stress (56%), schizophrenia (50%) and bipolar disorders (44%), consistent with anxiety being a common and often under-treated and under-appreciated factor in most mental health disorders.

### Phenomenology

The anxiety SAS-4 consists of four items (Supplementary Fig. S1). It correlates well with a standard scale in the field, the STAI State, with a Pearson correlation coefficient *R* = 0.67 (Supplementary Fig. S2A). Our clustering analysis revealed the structure of anxiety symptoms (Supplementary Fig. S3). Fear and Uncertainty were the most closely related. Anger is more distant and less related to other items on the scale. Anxiety reflects and underlies, in essence, if an individual is perceiving that they may be facing an adverse event, and are unhappy with their current state. Germane to that, we show that SAS-4 shows an inverse correlation with items of a visual analog scale for Life Satisfaction (Overall Satisfaction *R* = −0.58, Happiness *R* = −0.59, Hope *R* = −0.54, Meaning *R* = −0.59) (Fig. S2B).

### Diagnostics

**Fig. 3:**
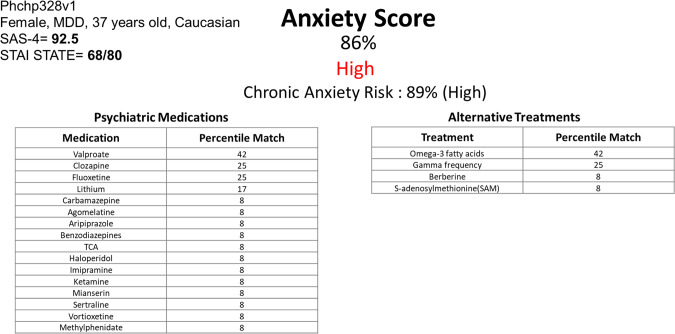
Example of a potential report for physicians. Using the panel of the top biomarkers for anxiety from Table 1 (*n* = 19). This subject (Phchp328) was previously described by us in a suicidality biomarker study, as high risk for suicide, and died by suicide a year after completing our study. No information was provided to the patient’s clinicians by us at that time due to anonymity and privacy rules in research studies. The raw expression values of the 19 biomarkers for the high and low anxiety groups were Z-scored by gender and diagnosis. We calculated as thresholds the average expression value for a biomarker in the high anxiety group SAS-4 ≥ 60, and in the low anxiety group SAS-4 ≤ 40. The first average should be higher than the second average in increased biomarkers, and the reverse is true for decreased biomarkers. 15 out of 19 biomarkers were thus concordant. We also calculated as thresholds the average expression value for a biomarker in the first-year hospitalizations group, and in the not hospitalized in first-year group. We did the same thing for all future hospitalizations, and no future hospitalizations. The first average should be higher than the second average in increased biomarkers, and the reverse is true for decreased biomarkers. 18 out of 19 biomarkers were thus concordant for first year, and for all future. The Z-scored expression value of each increased in expression biomarker was compared to the average value for the biomarker in the high anxiety group SAS-4 ≥ 60, and the average value of the low anxiety group SAS-4 ≤ 40, resulting in scores of 1 if above high anxiety, 0 if below low anxiety, and 0.5 if it was in between. The reverse was done for decreased in expression biomarkers. The digitized biomarkers were then added into a polygenic risk score and normalized for the number of biomarkers in the panel, resulting in a percentile score. We did the same thing for first-year hospitalizations, and all future hospitalizations, generating a combined score for chronic anxiety risk. The digitized biomarkers were also used for matching with existing psychiatric medications and alternative treatments (nutraceuticals and others). We used our large datasets and literature databases to match biomarkers to medications that had effects on gene expression opposite to their expression in high anxiety. Each medication matched to a biomarker got the biomarker score of 1, 0.5, or 0. The scores for the medications were added, normalized for the number of biomarkers that were 1 or 0.5 in that patient, resulting in a percentile match.

For the biomarkers identified by us, combining all the available evidence from this current work into a CFE score, brings to the fore biomarkers that have clinical utility for objective assessment and risk prediction for anxiety disorders (Table 1). These biomarkers should be tested individually as well as tested as polygenic panels of biomarkers in future clinical studies and practical clinical applications in the field. They may permit to distinguish, upon an initial clinical presentation of anxiety, whether the person is in fact severely anxious and at chronic risk (Fig. 3). The integration of phenomic data, such as repeated measures of SAS-4 (perhaps via a phone app in a daily fashion), can further substantiate and elucidate the diagnosis of anxiety disorders, distinguishing between an intermittent type such as panic attacks, and continuous type such as Generalized Anxiety Disorder.

In general, our predictive results with biomarkers were stronger in females than in males, by an order of 10–20% points on AUCs. While some of it may be biological, in terms of immune system reactivity and brain–blood interplay being perhaps higher in women, it is also possible that men are not as accurate as women in terms of self-reporting anxiety symptoms (affecting our results on state predictions), and do not seek help as much (affecting our results on future hospitalizations predictions). If so, this under-reporting makes the use of objective biomarker tests in men even more necessary.

In regards to how our biomarker discoveries might be applied in clinical laboratory settings, we suggest that panels of top biomarkers, such as BioM-19, be used (Fig. 3). In practice, every new patient tested would be normalized against the database of similar patients already tested, and compared to them for ranking and risk prediction purposes, regardless if a platform like microarrays, RNA sequencing, or a more targeted one like PCR is used in the end clinically. As databases get larger, normative population levels can and should be established, similar to any other laboratory measures. Moreover, longitudinal monitoring of changes in biomarkers within an individual, measuring most recent slope of change, maximum levels attained, and maximum slope of change attained in the past, may be even more informative than simple cross-sectional comparisons of levels within an individual with normative populational levels, as we have shown in our studies. For future point of care approaches, research and development should focus on top individual biomarkers, including at a protein level. One might look at the best universal biomarkers (that are predictive in all), for reliability, or at the best-personalized biomarkers (that are predictive by gender, and even diagnosis), for higher accuracy.

### Treatment

Biomarkers may also be useful for matching patients to medications and measuring response to treatment (pharmacogenomics) (Fig. 3, Tables 3 and S4), as well as new drug discovery and repurposing (Table 3). From the pharmacogenomics analyses, valproate was the top hit. Relatively recent randomized controlled clinical trial data is supportive of the use of valproate in anxiety disorders [24]. Other interesting novel candidates were omega-3 fatty acids, and lithium. From the drug repurposing analyses, estradiol was the top hit. Very recent randomized controlled clinical trial data is supportive of the use of estradiol in anxiety disorders [25]. Other interesting novel candidates were loperamide and disopyramide. All these drugs are relatively safe if used appropriately, and have been used in clinical practice for other indications for decades, which facilitates the direct translation to clinical practice of our findings.

## Conclusions

Overall, this work is a major step forward toward better understanding, diagnosing, and treating anxiety disorders. We hope that our trait biomarkers for future risk may be useful in preventive approaches, before the full-blown disorder manifests itself (or re-occurs). Prevention could be accomplished with social, psychological, or biological interventions (i.e., early targeted use of medications or nutraceuticals). Given the fact that 1 in 3 people will have a clinical anxiety disorder episode in their lifetime [26], that the prevalence seems to be increasing in younger people [27], that anxiety disorders can severely affect quality of life, sometimes leading to addictions such as alcoholism, and even suicides, and that not all patients respond to current treatments, the need for and importance of efforts such as ours cannot be overstated.

### Supplementary information

Supplementary Information - Figures S1-S4 and Tables S1- S4

Supplementary Information- Materials and Methods

Supplementary Information- Reproducibility
