# Supplementary material for: Towards precision medicine for anxiety disorders: objective assessment, risk prediction, pharmacogenomics, and repurposed drugs
Source: Mol Psychiatry. 2023 Mar 7;28(7):2894–912. doi: 10.1038/s41380-023-01998-0 (PMC10615756; doi:10.1038/s41380-023-01998-0)
Supplement: Supplementary file 1 — Supplementary Information - Figures S1-S4 and Tables S1- S4 [file 41380_2023_1998_MOESM1_ESM.docx]

**Supplementary Information**

**Figure S1: SAS-4 Scale for Measuring Anxiety State** (Niculescu et al. 2006, 2015). Score is average of 4 items. Ranges from 0-100.

**Figure S1.**

For each category, mark the scale with a short vertical line where you think you are at this moment in time, compared to lowest and highest you ever remember it being:

**1) Anxiety**

How anxious are you right now?

**Lowest [-----------------------------------------------------------------------] Highest**

**0 100**

**2) Fear**

How frightened about things do you feel right now?

**Lowest [-----------------------------------------------------------------------] Highest**

**0 100**

**3) Anger**

How angry about things do you feel right now?

**Lowest [-----------------------------------------------------------------------] Highest**

**0 100**

**4) Uncertainty**

How uncertain about things do you feel right now?

**Lowest [-----------------------------------------------------------------------] Highest**

**0 100**

**Figure S2. Correlation between SAS-4 and other measures of Anxiety and Life Satisfaction. A. Correlation with STAI State.** Our SAS4 scale shows a strong correlation with a well-validated clinical measure of anxiety, the STAI State, while being more temporally related to that particular moment in time, quantitative, and simple. **B. Correlation with Life Satisfaction.** SAS-4 is moderately negatively correlated with self-reported measures of life satisfaction.

**A. Correlation between SAS-4 and STAI State.**

| R- Pearson Correlation Coefficient | 0.667503766 |
| --- | --- |
| Sample Size | 784 |
| Test Statistic | 25.06861378 |
| p-value | 1.4989E-102 |

**B. Anxiety and Life Satisfaction**

| Pearson Correlation R  (n=191)  all p<0.001 | Overall Satisfaction | Happiness | Hope | Meaning |
| --- | --- | --- | --- | --- |
| SAS-4 | -0.58 | -0.59 | -0.54 | -0.59 |

**Figure S3. SAS-4 items clustering.** Two way unsupervised hierarchical clustering in the Discovery Cohort. The vertical clustering is of patient visits into high and low anxiety ones. The horizontal clustering is of SAS-4 items.


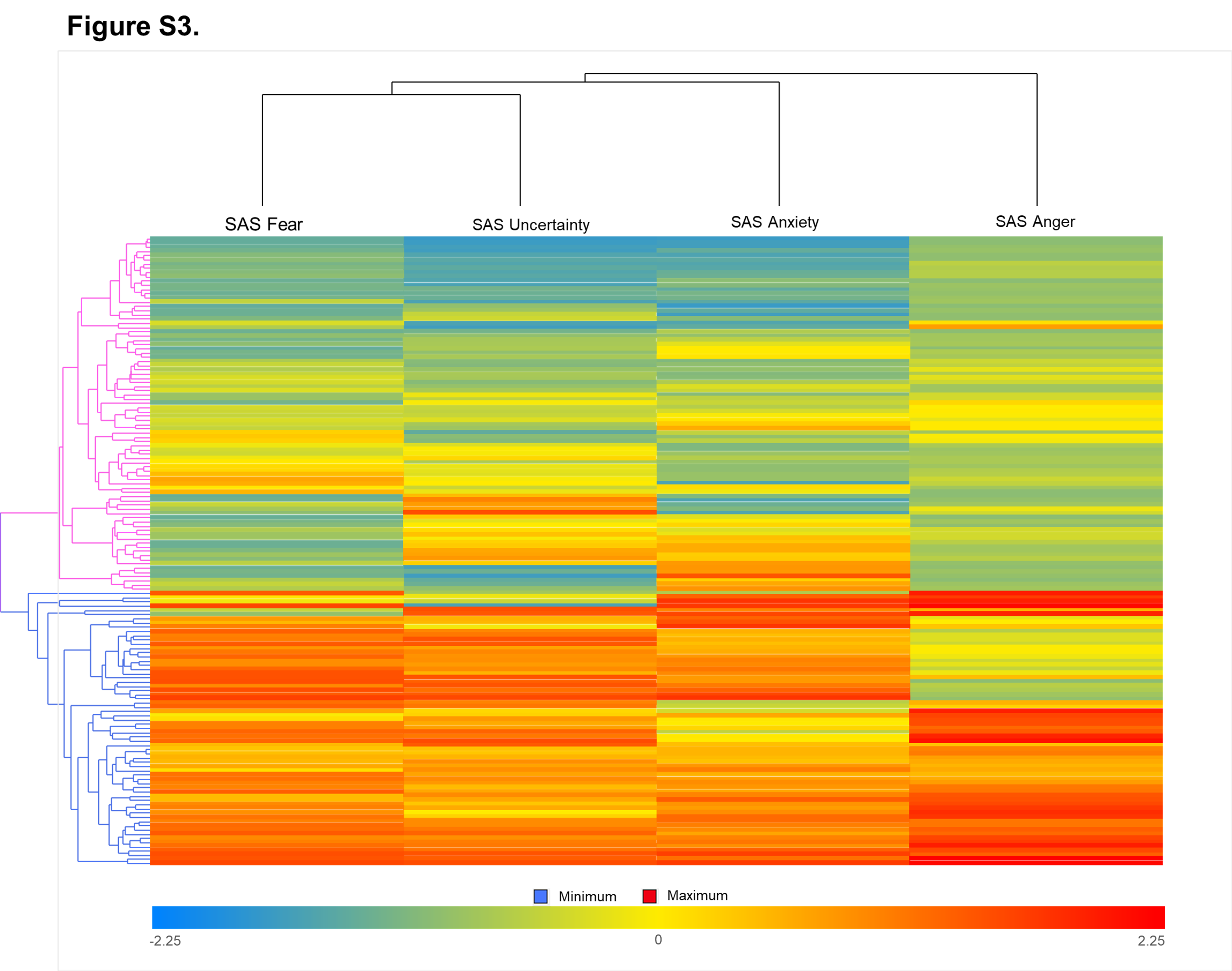


**Figure S4. Network Analysis of Top Candidate Biomarkers after Step 3 (n=95 probesets, in 82 genes).** 3 networks are delineated.

**
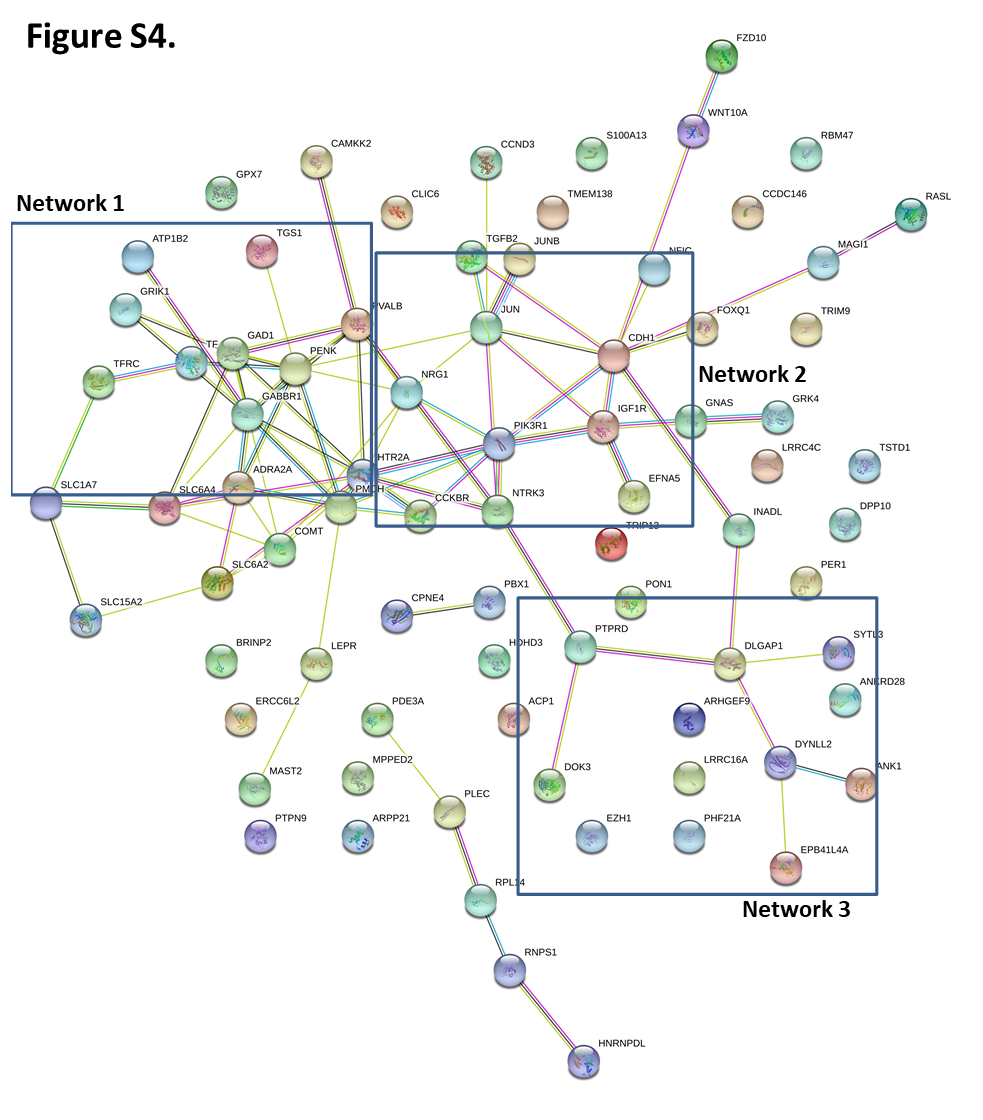
**

**Table S1. Demographics and Anixety Comorbidity A.** Aggregate Demographics BP-bipolar, MDD- depression, MOOD- mood nos, SZ- schizophrenia, SZA- schizoaffective, PSYCH- psychosis nos, PTSD-post-traumatic stress disorder. **B.** Percentile of each diagnosis group that have a co-morbid clinical anxiety disorder diagnosis (anxiety disorder NOS, panic disorder, GAD)

**A. Aggregate Demographics**

|  | **Number of Subjects** | **Gender** | **Diagnosis** | **Ethnicity** | **Age Mean**  **(SD)** | **T-test for age** |
| --- | --- | --- | --- | --- | --- | --- |
| **Discovery** | | | | | | |
| **Discovery Cohort**  **(Longitudinal Within-Subject Changes**  **Anxiety Levels**  **(SAS-4)** | 58  (149 visits) | Male = 41  Female =17 | BP= 18(52) MDD= 10(23) SZA= 14(34) SZ= 7(17) PTSD= 7(17)  Mood = 2(6) | EA= 41  AA= 14  Mixed = 1  Asian American = 1  Hispanic = 1 | All = 49.6  (8.63)  Low Anxiety = 49.7 (8.77)  High Anxiety = 49.4 (8.51) |  |
| **Validation** | | | | | | |
| **Independent Validation Cohort for Gene Expression**  **(Clinically Severe Anxiety)**  **SAS-4 (≥60)**  **STAI (≥55)** | 40  (60 visits) | Male = 32  Female = 8 | BP= 15(18) MDD= 6(9)  SZA= 6(10) SZ= 3(7) PTSD= 8(14) MOOD= 2(2) | EA= 29  AA= 11 | 47.9  (8.4) | Discovery vs. Validation =  **0.20636** |
| **Testing** | | | | | | |
| **Independent Testing Cohort For Predicting State**  **High Anxiety**  **SAS-4 (≥60)** | 197  (495 visits) | Male = 161 Female = 36 | BP=60 (171) MDD=34 (78)  SZA=42 (100)  SZ=40 (101)  PTSD=13 (26)  MOOD=3 (8)  PSYCH=5 (11) | EA= 132  AA= 62  Hispanic=2  Asian=1 | All = 50.07  (9.7)  High Anxiety = 48.6 (n=19)  Others = 50.1  (n=476) | T-test for age between High Anxiety (SAS-4)  vs. Others  **0.542** |
| **Independent Testing Cohort For Predicting State**  **Clinical Anxiety**  **STAI State (≥55)** | 195  (486 visits) | Male = 159 Female = 36 | BP= 60(170) MDD= 34(78)  SZA= 41(95)  SZ= 39(98)  PTSD= 13(26)  MOOD= 3(8)  PSYCH= 5(11) | EA= 131  AA= 61  Hispanic=2  Asian=1 | All = 50.04  (9.7)  High Anxiety (STAI) = 50.9 (n=42)  Others= 50  (n=444) | T-test for age between High Anxiety (STAI)  vs. Others  **0.9673** |
| Independent Testing Cohort  For Predicting Trait  (**Hospitalization with Anxiety in the First Year Following Assessment**) | 101  (236 Visits) | Male = 86  Female = 15 | BP = 34 (94) MDD= 19(40)  SZA= 17 (38)  SZ= 22 (49)  PTSD= 9 (15) | EA= 73  AA= 26  Hispanic =2 | All= 44.5  (8.11)  Anxiety Hosp= 43 (n=18)  Others = 44.5 (n=218) | T-test for age  Hosp with Anxiety vs. others  First year  **p= 0.59098** |
| Independent Testing Cohort  For Predicting Trait  (**All Future Hospitalizations with Anxiety**) | 130  (318 Visits) | Male= 118  Female= 18 | BP= 39 (107) MDD= 28 (59)  SZA= 28 (64)  SZ= 31 (70)  PTSD= 10 (18) | EA=90  AA= 44  Hispanic =2 | All= 47.4  (5.64)  Anxiety Future Hosp= 47.2 (n=70)  Others = 47.5 (n=248) | T-test for age  Hosp with no Anxiety vs. vs. Others  All Future Years  **0.842276** |

**B.** Co-morbid clinical anxiety disorder diagnosis

| **Primary Diagnosis** | **Percentage of patients with co-morbid clinical anxiety diagnosis** |
| --- | --- |
| MDD | 37.30% |
| MOOD D/O NOS | 28.60% |
| BP | 22.10% |
| SZA | 21% |
| PTSD | 20.70% |
| SZ | 10% |
| PSYCHOSIS NOS | 0% |
| All | 22% |

**Table S2. Step 2: CFG-Prior evidence for involvement in anxiety .** Top biomarkers from Table 2. Red- increased in expression (I) in Anxiety, Blue- decreased in expression (D). DE- differential expression, AP-Absent/Present.

| **Symbol/ Gene Name** | **Probesets** | **Step 1 Discovery in Blood  (Direction of Change in High Anxiety) Method/ Score/ %  6pts** | **Step 2 External Convergent Functional Genomics (CFG) Evidence For Involvement in Anxiety  Score  12pts** | **Step 2**  **Prior human genetic evidence for Anxiety**  **2 ptts** | **Step 2**  **Prior human Brain expression evidence for Anxiety 4 pts** | **Step 2**  **Prior human peripheral evidence for Anxiety 2pts** | **Step 2**  **Prior Non-human genetic evidence for Anxiety 1pt** | **Step 2**  **Prior Non-human Brain expression evidence for Anxiety 2pts** | **Step 2**  **Prior Non-human peripheral evidence for Anxiety 1pt.** | **Step 3 Validation in Blood  ANOVA p-value/ Score  6 pts** | **CFE Polyevidence Score for Involvement in Anxiety (Based on Steps 1-4)** |
| --- | --- | --- | --- | --- | --- | --- | --- | --- | --- | --- | --- |
| **GAD1** Glutamate Decarboxylase 1 | 205278_at | (I)  DE/4 62.3% | 11 | **Anxiety** [^1^](#_ENREF_1)  **Panic Disorder** [^1^](#_ENREF_1) | (I)  medial orbitofrontal cortex  **OCD**  [^2^](#_ENREF_2) | (I)  Blood  **Panic Disorder**  [^3^](#_ENREF_3) | **Anxiety**  [^4^](#_ENREF_4) | (D)  Dentate Gyrus  **Anxiety** [^4^](#_ENREF_4)  (D)  basolateral amygdala (BLA)  CpG hypermethylation on GAD67 promoters **Anxiety** [^5^](#_ENREF_5) |  | 6.79E-01/0 Not Stepwise | 22 |
| **NTRK3** Neurotrophic Receptor Tyrosine Kinase 3 | 215311_at | (I)  DE/4 53.2% | 4 | **OCD** [^6^](#_ENREF_6)  **OCD** [^7^](#_ENREF_7)  **Panic Disorder** [^7^](#_ENREF_7) |  |  | **Panic Disorder** [^8^](#_ENREF_8)  **Anxiety** [^9^](#_ENREF_9) | (I)  Cingulate Cortex **Antidepressants**  [^10^](#_ENREF_10) |  | 3.26E-01/2 Stepwise | 19 |
| **ADRA2A** Adrenoceptor Alpha 2A | 209869_at | (I) AP/4 50% | 4 | **Anxiety** [^11^](#_ENREF_11) |  |  | (I) cortex **Anxiety**[^12^](#_ENREF_12) |  |  | 9.07E-01/0 Not Stepwise | 17 |
| **FZD10** Frizzled Class Receptor 10 | 219764_at | (I)  DE/4 53.2% | 2 |  |  |  | (D) telencephalon **Anxiety**[^13^](#_ENREF_13) |  |  | 3.57E-01/2 Stepwise | 17 |
| **GRK4** G Protein-Coupled Receptor Kinase 4 | 210600_s_at | (I)  DE/6 81.8% | 0 |  |  |  |  |  |  | 3.40E-01/2 Stepwise | 17 |
| **ATP1B2** ATPase Na+/K+ Transporting Subunit Beta 2 | 204311_at | (I)  DE/2 39% | 4 |  |  | (D) Lymphocyte **Panic Disorder** [^14^](#_ENREF_14) | (D) Hippocampus **Anxiety** [^15^](#_ENREF_15) |  |  | 1.65E-01/2 Stepwise | 16 |
| **CLIC6** Chloride Intracellular Channel 6 | 242913_at | (I) AP/6 84.2% | 2 |  |  |  | (I) Hippocampus anf PFC **Anxiety**[^16^](#_ENREF_16)  (D) Hippocampus **Anxiety**[^15^](#_ENREF_15) |  |  | 5.06E-01/0 Not Stepwise | 16 |
| **EFNA5** Ephrin A5 | 1559360_at | (I)  DE/6 80.5% (I) AP/4 51.3% | 2 |  |  | (I)  Lymphocyte **Panic Disorder** [^14^](#_ENREF_14) |  |  |  | 2.02E-04/4 Nominal | 16 |
| **GPX7** Glutathione Peroxidase 7 | 213170_at | (D) DE/4 64.3% | 2 |  |  |  | (I) Medullae Oblongata **Panic Disorder** [^17^](#_ENREF_17) |  |  | 4.64E-01/2 Stepwise | 16 |
| **NTRK3** Neurotrophic Receptor Tyrosine Kinase 3 | 215025_at | (I)  DE/2 35.1% | 4 | **OCD** [^6^](#_ENREF_6)  **OCD** [^7^](#_ENREF_7)  **Panic Disorder** [^7^](#_ENREF_7) |  |  | **Panic Disorder** [^8^](#_ENREF_8)  **Anxiety** [^9^](#_ENREF_9) | (I)  Cingulate Cortex **Antidepressants**  [^10^](#_ENREF_10) |  | 6.58E-01/2 Stepwise | 16 |
| **SLC6A2** Solute Carrier Family 6 Member 2 | 217214_s_at | (I)  DE/2 40.3% | 4 | **Panic Disorder** [^18^](#_ENREF_18) |  | (D) Blood **Panic Disorder**  [^19^](#_ENREF_19) |  |  |  | 3.40E-01/2 Stepwise | 16 |
| **SLC6A4** Solute Carrier Family 6 Member 4 | 242009_at | (I)  DE/2 35.1% | 10 | **Anxiety** [^20^](#_ENREF_20)  **OCD** [^21^](#_ENREF_21) | (D)  raphe, temporal cortex, and thalamus  **Panic Disorder** [^22^](#_ENREF_22) | (I)  Blood **Anxiety** [^23^](#_ENREF_23)  (D)  Buccal **Anxiety** [^24^](#_ENREF_24) Buccal  (D)  **Anxiety** [^24^](#_ENREF_24) Blood  (D)  **OCD** [^25^](#_ENREF_25)  (I) lymphoblastoid  **OCD**  [^26^](#_ENREF_26) |  | (I)  Hippocampus  **Anxiety** [^27^](#_ENREF_27)  (D)  NO TISSUE behavioral testing **Anxiety** [^28^](#_ENREF_28)  (I)  hippocampus  **Anxiety**  [^29^](#_ENREF_29) |  | 8.51E-01/0 Not Stepwise | 16 |
| **TMEM138** Transmembrane Protein 138 | 223113_at | (D)  DE/4 63% | 4 |  |  | (I) Lymphocyte **Panic Disorder** [^14^](#_ENREF_14) |  | (I) Medullae Oblongata **Panic Disorder** [^17^](#_ENREF_17) |  | 8.63E-01/0 Not Stepwise | 16 |
| **240253_at** | 240253_at | (I)  DE/6 88.3% | 0 |  |  |  |  |  |  | 2.74E-01/2 Stepwise | 15 |
| **ANKRD28** Ankyrin Repeat Domain 28 | 229307_at | (I)  DE/6 80.5% | 0 |  |  |  |  |  |  | 6.20E-01/2 Stepwise | 15 |
| **CCKBR** Cholecystokinin B Receptor | 210381_s_at | (I)  DE/2 48% | 4 | **Panic Disorder** [^30^](#_ENREF_30), [^31^](#_ENREF_31), [^32^](#_ENREF_32), [^33^](#_ENREF_33), [^34^](#_ENREF_34), [^35^](#_ENREF_35) |  |  | (I) Forebrain **Anxiety** [^36^](#_ENREF_36)  (I) Cortex, Hippocampus , hypothalamus **Anxiety** [^37^](#_ENREF_37)  (I)  Hippocampus  **Yohimbine** [^38^](#_ENREF_38) |  |  | 4.37E-01/2 Stepwise | 15 |
| **DYNLL2** Dynein Light Chain LC8-Type 2 | 229106_at | (D)  DE/2 39.3% | 4 | **Anxiety** [^39^](#_ENREF_39) |  |  | (D) PERIAQUEDUCTAL **Anxiety** [^40^](#_ENREF_40)  (I) AMY  **Benzodiazepines** [^38^](#_ENREF_38) |  |  | 1.96E-01/2 Stepwise | 15 |
| **NRG1** Neuregulin 1 | 208232_x_at | (I)  DE/4 65.8% | 4 | **Anxiety** [^41^](#_ENREF_41) |  |  | (I) mPFC, PL Cortex **Fear** [^42^](#_ENREF_42) |  |  | 6.66E-01/2 Stepwise | 15 |
| **TFRC** Transferrin Receptor | 207332_s_at | (D) DE/4 56% | 2 |  |  |  | (I)  basal forebrain  **TCA**  [^43^](#_ENREF_43)  (D)  Hippocampus  **Benzodiazepines**  [^38^](#_ENREF_38) |  |  | 7.11E-01/2 Stepwise | 15 |

**Table S3. Evidence for involvement in other psychiatric and related disorders .** Top biomarkers from Table 2. In the same direction of expression as high anxiety**.** Red- increased in expression (I) in Anxiety, Blue- decreased in expression (D). DE- differential expression, AP-Absent/Present. MDD- Depression; BP- bipolar; SZ- schizophrenia.

| **Symbol/ Gene Name** | **Probesets** | **Step 1 Discovery in Blood  (Direction of Change in High Anxiety) Method/ Score/ %  6pts** | **Step 2 External Convergent Functional Genomics (CFG) Evidence For Involvement in Anxiety  Score  12pts** | **Step 3 Validation in Blood  ANOVA p-value/ Score  6 pts** | **CFE Polyevidence Score for Involvement in Anxiety (Based on Steps 1-4)** |  | **Prior human genetic evidence for other disorder** | **Prior human Brain expression evidence for other disorder** | **Prior human peripheral evidence for other disorder** | **Prior Non-human genetic evidence for other disorder** | **Prior Non-human Brain expression evidence for other disorder** | **Prior Non-human peripheral evidence for other disorder** |
| --- | --- | --- | --- | --- | --- | --- | --- | --- | --- | --- | --- | --- |
| **GAD1** Glutamate Decarboxylase 1 | 205278_at | (I)  DE/4 62.3% | 11 | 6.79E-01/0 Not Stepwise | 22 |  | **Alcohol**  [^44^](#_ENREF_44)  **SZ** [^45^](#_ENREF_45)  **BP** [^46^](#_ENREF_46)  **Depression**  [^47^](#_ENREF_47)  **MDD** [^1^](#_ENREF_1)  **Autism** [^48^](#_ENREF_48)  **Intellectual Disability** [^49^](#_ENREF_49)  **PTSD** [^50^](#_ENREF_50)  **Creativity**[^51^](#_ENREF_51)  **Depression Females** [^52^](#_ENREF_52) | (I)  BA 9  **SZ** [^53^](#_ENREF_53)  (I)  DLPFC  **BP, SZ** [^54^](#_ENREF_54)  (I) dorsolateral PFC , superior temporal cortex, hippocampus  **Mood Disorders NOS** [^55^](#_ENREF_55)  (I) orbitofrontal cortex **Mood Disorders NOS** [^55^](#_ENREF_55)  (I)  DLPFC **MDD** [^56^](#_ENREF_56)  (I)  DLPFC, ACC **Depression Suicide** [^56^](#_ENREF_56)  (I)  DLPFC  **SZ** [^57^](#_ENREF_57)  (I) Hippocampal formation **Suicide** [^58^](#_ENREF_58) | (I) neuroblastoma  **SZ**  [^59^](#_ENREF_59)  (I)  Blood  **SZ**  [^60^](#_ENREF_60)  (I)  L neurons **BP**  [^61^](#_ENREF_61)  (I)  Serum  **BP** [^62^](#_ENREF_62)  (I) Lymphocyte  **SZ** 34368786 [^63^](#_ENREF_63) | **Intellectual Disability**  [^49^](#_ENREF_49) | (I) Locus Coeruleus **MDD** [^64^](#_ENREF_64)  (I) AMY **Phencyclidine** [^65^](#_ENREF_65)  (I) nucleus accumbens (NAC), PFC **Alcohol**  [^66^](#_ENREF_66)  (I) Frontal Cortex **Alcohol** 24148570 [^67^](#_ENREF_67)  (I) Central Nucleus of the Amygdala **Alcohol Withdrawal, Alcohol** [^68^](#_ENREF_68) | (I) Prefrontal Cortex, Amygdala, Caudate-putamen, Hippocampus  **SZ**  [^69^](#_ENREF_69)  (I) PFC, Hippocampus  **SZ** [^70^](#_ENREF_70) |
| **NTRK3** Neurotrophic Receptor Tyrosine Kinase 3 | 215311_at | (I)  DE/4 53.2% | 4 | 3.26E-01/2 Stepwise | 19 |  | **SZ**  [^71^](#_ENREF_71)  **Bipolar II** [^72^](#_ENREF_72)  **BP** [^73^](#_ENREF_73)  **MDD**  [^74^](#_ENREF_74)  **Mood Disorders NOS** [^75^](#_ENREF_75)  **Suicide** [^76^](#_ENREF_76)  **SZ** [^77^](#_ENREF_77)  **Risky Behavior** [^78^](#_ENREF_78)  **SZ** [^77^](#_ENREF_77)  **MDD** [^79^](#_ENREF_79)  **Longevity**  [^80^](#_ENREF_80)  **Alcohol**  [^81^](#_ENREF_81)  **Depression Males and Females**  [^52^](#_ENREF_52) | (I)  CA1-Stratum oriens **BP** [^82^](#_ENREF_82)  (I) Hippocampus **Alzheimer's Disease** [^83^](#_ENREF_83)  (I)  NAC **Social Isolation** [^84^](#_ENREF_84) | (I) Plasma **Aging** [^85^](#_ENREF_85)  (I) Blood **PTSD** [^86^](#_ENREF_86)  (I) Blood **Subsyndromal symptomatic depression, MDD**  [^87^](#_ENREF_87) | **SZ** [^88^](#_ENREF_88) | (I) NAC **Alcohol** [^89^](#_ENREF_89)  (I) hippocampus **Stress** [^90^](#_ENREF_90)  (I) Cortical plate, posterior developing cerebral cortex, Hippocampus, cerebellum (granular cell layer), frontal cotex **SZ** [^88^](#_ENREF_88)  (I) Male Nac **Stress** [^91^](#_ENREF_91)  (I) Brain **SAD** [^92^](#_ENREF_92)  (I) NAC **Alcohol** [^66^](#_ENREF_66)  (I) Frontal Cortex **Alcohol** [^67^](#_ENREF_67)  (I) CeA **Alcohol** [^93^](#_ENREF_93) | (I) Spinal cord and DRG-RNA **Pain** [^94^](#_ENREF_94) |
| **ADRA2A** Adrenoceptor Alpha 2A | 209869_at | (I) AP/4 50% | 4 | 9.07E-01/0 Not Stepwise | 17 |  | **Alcohol** [^95^](#_ENREF_95)  **Depression** [^96^](#_ENREF_96)  **Pain** [^97^](#_ENREF_97)  **Suicide** [^98^](#_ENREF_98)  **SZ** [^77^](#_ENREF_77)  **Longevity**  [^80^](#_ENREF_80) | (I)  Male BA25 **MDD**  [^91^](#_ENREF_91)  (I) Frontal Cortex **Suicide** [^99^](#_ENREF_99)  (I)  PFC (BA 9) **Suicide** [^100^](#_ENREF_100)  (I)  PFC (BA 9, middle frontal gyrus) **Suicide** [^101^](#_ENREF_101) | (I) Blood **PTSD** [^102^](#_ENREF_102)  (I)  Lung and blood **Bipolar I, SZ and COVID-19** [^103^](#_ENREF_103) |  | (I) NAC **Phencyclidine** [^104^](#_ENREF_104)  (I) PFC **Alcohol** [^66^](#_ENREF_66)  (I) PFC **Alcohol**  [^93^](#_ENREF_93)  (I) Neural Stem Cells From Forebrain **Alcohol** [^105^](#_ENREF_105) | (I) Spinal cord and DRG-RNA **Pain** [^94^](#_ENREF_94) |
| **FZD10** Frizzled Class Receptor 10 | 219764_at | (I)  DE/4 53.2% | 2 | 3.57E-01/2 Stepwise | 17 |  | **Circadian abnormalities** [^106^](#_ENREF_106)  **Circadian abnormalities** [^107^](#_ENREF_107)  **Alcohol** [^81^](#_ENREF_81)  **BP**  [^81^](#_ENREF_81) | (I)  Male BA8/9 **MDD**  [^91^](#_ENREF_91)  (I) Hippocampus **ALZ** [^83^](#_ENREF_83) | (I) Blood **PTSD** [^86^](#_ENREF_86) |  | (I) AMY **MDD** [^108^](#_ENREF_108) |  |
| **GRK4** G Protein-Coupled Receptor Kinase 4 | 210600_s_at | (I)  DE/6 81.8% | 0 | 3.40E-01/2 Stepwise | 17 |  | **Autism** [^48^](#_ENREF_48)  **Income** [^109^](#_ENREF_109)  **Depression Females** [^52^](#_ENREF_52) | (I)  PFC **Bipolar**  [^110^](#_ENREF_110) | hypermethylation blood **PTSD**  [^111^](#_ENREF_111)  (I) Monocytes **Cannabis** [^112^](#_ENREF_112) |  | (I) Female PFC **Stress**[^91^](#_ENREF_91) |  |
| **ATP1B2** ATPase Na+/K+ Transporting Subunit Beta 2 | 204311_at | (I)  DE/2 39% | 4 | 1.65E-01/2 Stepwise | 16 |  |  | (I) ACC **MDD**  [^113^](#_ENREF_113)  (I) Hippocampus  **Suicide** [^114^](#_ENREF_114)  (I) cerebral cortex **SZ** [^48^](#_ENREF_48)  (I) Frontal Lobe **ALZ** [^115^](#_ENREF_115)  (I) DLPFC, HIP  **SZ** [^116^](#_ENREF_116)   (I) DLPFC **Substance Abuse** [^117^](#_ENREF_117)  (I) DLPFC **Bipolar** [^118^](#_ENREF_118)  (I)  Pre-frontal cortex, Orbitofrontal cortex **Bipolar**  [^118^](#_ENREF_118)  (I) Hippocampus **Male SZ** [^118^](#_ENREF_118) | (I) Blood **PTSD** [^102^](#_ENREF_102)  (I) PBMC **Relaxation Response** [^119^](#_ENREF_119)  (I) embryonic stem cells, Lumbar spinal cord **ALS** [^118^](#_ENREF_118)  (I)  SH-SY5Y cells **Alcohol** [^120^](#_ENREF_120) |  | (I)  HIP (Females) **Stress** [^121^](#_ENREF_121)  (I) AMY,CP, HIP  **Alcohol** [^65^](#_ENREF_65)  (I) basal nucleus of stria terminalis (BNST), HIP, NAC **Alcohol**  [^66^](#_ENREF_66) |  |
| **CLIC6** Chloride Intracellular Channel 6 | 242913_at | (I) AP/6 84.2% | 2 | 5.06E-01/0 Not Stepwise | 16 |  | **SZ** 28991256 | (I) cerebral cortex **MDD**  [^48^](#_ENREF_48)  (I) Hippocampus **Cocaine** [^122^](#_ENREF_122)  (I) Hippocampus **Suicide** [^123^](#_ENREF_123) | (I) PBMCs **Aging** [^124^](#_ENREF_124)  (I) Lymphoblastoid cell lines **Alcohol** [^125^](#_ENREF_125) |  | (I) Hippocampus **MDD** [^126^](#_ENREF_126) (I) Hippocampus  **Restraint Stress** [^127^](#_ENREF_127)   (I)  VT  **Phencyclidine** [^104^](#_ENREF_104)  (I) Hippocampus **Alcohol** [^66^](#_ENREF_66)  (I) frontal pole synaptoneurosomes **Alcohol**  [^128^](#_ENREF_128) |  |
| **EFNA5** Ephrin A5 | 1559360_at | (I)  DE/6 80.5% (I) AP/4 51.3% | 2 | 2.02E-04/4 Nominal | 16 |  | **Insomnia** [^129^](#_ENREF_129)  **Alcohol** [^130^](#_ENREF_130)  **Longevity** [^131^](#_ENREF_131)  **Chronic Fatigue Syndrome**  [^132^](#_ENREF_132)  **Suicide** [^76^](#_ENREF_76)  **Intellect** [^133^](#_ENREF_133)  **ALZ**  [^134^](#_ENREF_134)  **BP** [^135^](#_ENREF_135)  **First Sexual Intercourse**  [^136^](#_ENREF_136)  **Longevity**  [^80^](#_ENREF_80)  **Alcohol** [^81^](#_ENREF_81)  **Depression Females** [^52^](#_ENREF_52) | (I) Hippocampus  **BP**  [^137^](#_ENREF_137)  (I)  PFC, OFC, sgACC  **MDD**  [^138^](#_ENREF_138) | (I)  Blood **Pain**  [^139^](#_ENREF_139)  (I) Fibroblast  **SZ**  [^140^](#_ENREF_140)  (I)  Blood **Female Suicide**  [^141^](#_ENREF_141)  (I)  Blood **Stress**  [^139^](#_ENREF_139)  (I) Monocytes **Cannabis** [^112^](#_ENREF_112)  (I) Lymphoblastoid cell lines **Alcohol** [^125^](#_ENREF_125) |  | (I) Hippocampus  **ALZ** [^142^](#_ENREF_142)  (I) dmPFC (Males)  **Morphine** [^143^](#_ENREF_143)  (I) basal nucleus of stria terminalis (BNST), central nucleus of amygdala (CeA), NAC  **Alcohol**   [^66^](#_ENREF_66)  (I) Frontal Cortex **Alcohol** [^67^](#_ENREF_67)  (I) CeA **Alcohol** [^93^](#_ENREF_93) |  |
| **GPX7** Glutathione Peroxidase 7 | 213170_at | (D) DE/4 64.3% | 2 | 4.64E-01/2 Stepwise | 16 |  |  | (D) Anterior PFC **MDD SZA** [^144^](#_ENREF_144) | (D) **Aging**  [^145^](#_ENREF_145) |  | (D) PFC **Neuropathic Pain** [^146^](#_ENREF_146) |  |
| **NTRK3** Neurotrophic Receptor Tyrosine Kinase 3 | 215025_at | (I)  DE/2 35.1% | 4 | 6.58E-01/2 Stepwise | 16 |  | **SZ**  [^71^](#_ENREF_71)  **Bipolar II** [^72^](#_ENREF_72)  **BP** [^73^](#_ENREF_73)  **MDD**  [^74^](#_ENREF_74)  **Mood Disorders NOS** [^75^](#_ENREF_75)  **Suicide** [^76^](#_ENREF_76)  **SZ** [^77^](#_ENREF_77)  **Risky Behavior** [^78^](#_ENREF_78)  **SZ**  [^77^](#_ENREF_77)  **MDD** [^79^](#_ENREF_79)  **Longevity**  [^80^](#_ENREF_80)  **Alcohol**  [^81^](#_ENREF_81)  **Depression Males and Females** [^52^](#_ENREF_52) | (I) CA1-Stratum oriens **BP** [^82^](#_ENREF_82)  (I) Hippocampus **Alzheimer's Disease** [^83^](#_ENREF_83)  (I)  NAC **Social Isolation** [^84^](#_ENREF_84) | (I) Plasma **Aging** [^85^](#_ENREF_85)  (I) Blood **PTSD** [^86^](#_ENREF_86)  (I) Blood **Subsyndromal symptomatic depression** [^87^](#_ENREF_87)  (I) Blood **MDD** [^87^](#_ENREF_87) | **SZ** [^88^](#_ENREF_88) | (I) NAC **Alcohol** [^89^](#_ENREF_89)  (I) hippocampus **Stress** [^90^](#_ENREF_90)  (I) Cortical plate, posterior developing cerebral cortex, Hippocampus, cerebellum (granular cell layer), frontal cotex **SZ**  [^88^](#_ENREF_88)  (I) Male Nac **Stress** [^91^](#_ENREF_91)  (I) Brain **SAD** [^92^](#_ENREF_92)  (I) NAC **Alcohol** [^66^](#_ENREF_66)  (I) Frontal Cortex **Alcohol**  [^67^](#_ENREF_67)  (I) CeA **Alcohol**  [^93^](#_ENREF_93) | (I) Spinal cord and DRG-RNA **Pain** [^94^](#_ENREF_94) |
| **SLC6A2** Solute Carrier Family 6 Member 2 | 217214_s_at | (I)  DE/2 40.3% | 4 | 3.40E-01/2 Stepwise | 16 |  | **Alcohol** [^95^](#_ENREF_95)  **MDD**  [^147^](#_ENREF_147)  **MDD** [^148^](#_ENREF_148)  **Suicide** [^149^](#_ENREF_149)  **PTSD**  [^150^](#_ENREF_150)  **Female Depression**  [^52^](#_ENREF_52) | (I)  Brain **Female Depression** [^151^](#_ENREF_151) | (I)  SH-SY5Y HUMAN NEUROBLASTOMA **Alcohol**[^152^](#_ENREF_152)  (I) SH-SY5Y cells **Alcohol**[^120^](#_ENREF_120) |  | (I)  M nucleus accumbens **Alcohol** [^153^](#_ENREF_153)  (I) whole brain **Postpartum Depression** [^154^](#_ENREF_154) |  |
| **SLC6A4** Solute Carrier Family 6 Member 4 | 242009_at | (I)  DE/2 35.1% | 10 | 8.51E-01/0 Not Stepwise | 16 |  | **Alcohol** [^155^](#_ENREF_155)  **Aging**  [^156^](#_ENREF_156)  **Longevity**  [^157^](#_ENREF_157)  **ASD** [^158^](#_ENREF_158)  **SZ** [^159^](#_ENREF_159)  **BP** [^160^](#_ENREF_160)  **Depression**  [^161^](#_ENREF_161)  **MDD**  [^162^](#_ENREF_162)  **Affective Disorder** [^163^](#_ENREF_163)  **Mood Disorders NOS** [^164^](#_ENREF_164)  **Aggression**  [^165^](#_ENREF_165)  **Borderline Personality** **Disorder** [^166^](#_ENREF_166)  **Neuroticism**) [^167^](#_ENREF_167)  **MSK Pain** [^168^](#_ENREF_168)  **Neuropathic Pain**  [^169^](#_ENREF_169)  **Other disease/lesion Pain** [^170^](#_ENREF_170)  **Pain** [^171^](#_ENREF_171)  **PTSD** [^172^](#_ENREF_172)  **Suicide**  [^173^](#_ENREF_173)  **PTSD**  [^150^](#_ENREF_150)  **Autism** [^48^](#_ENREF_48)  **Intellect** [^133^](#_ENREF_133)  **MDD** [^174^](#_ENREF_174)  **BP** [^175^](#_ENREF_175)  **Depression Females** [^52^](#_ENREF_52)  **Suicide Attempt** [^176^](#_ENREF_176) | (I)  Female Subic  **MDD** [^91^](#_ENREF_91)  (I) Hippocampus  **Suicide** [^177^](#_ENREF_177) | (I) PBMCs  **Aging** [^124^](#_ENREF_124)  hypermethylated Blood **Early Life Stress** [^178^](#_ENREF_178)  hypermethylated Blood **Depression**  [^178^](#_ENREF_178)  (I) placenta  **MDD** [^179^](#_ENREF_179)  (I) peripheral blood mononuclear cells **MDD**  [^180^](#_ENREF_180)  (I)  Blood **MDD**  [^181^](#_ENREF_181)  (I) peripheral blood mononuclear cells (PBMCs)  **MDD**, [^180^](#_ENREF_180)  (I) Blood **Alcohol**  [^182^](#_ENREF_182)  (I) Blood **Stress** [^23^](#_ENREF_23)  (I) Lymphoblastoid cell lines **Alcohol** [^125^](#_ENREF_125) | **ASD** [^183^](#_ENREF_183)  **Alcohol** [^184^](#_ENREF_184) | (I) PFC **MDD**[^185^](#_ENREF_185)  (I) Embryonic hippocampal and prefrontal cortex neurons  **MDD** [^186^](#_ENREF_186)  (I) whole brain **Postpartum Depression** [^154^](#_ENREF_154)  (I) hippocampus  **Alcohol** [^29^](#_ENREF_29)  (I) Dorsal Raphe Nucleus **PTSD** [^187^](#_ENREF_187) | (I) Lymphocytes  **Phencyclidine**  [^104^](#_ENREF_104) |
| **TMEM138** Transmembrane Protein 138 | 223113_at | (D)  DE/4 63% | 4 | 8.63E-01/0 Not Stepwise | 16 |  | **Brain arousal** [^188^](#_ENREF_188)  **Female Depression** [^52^](#_ENREF_52) | (D) Hippocampus **Alcohol** [^122^](#_ENREF_122) |  |  | (D) CeA **Alcohol**  [^93^](#_ENREF_93)  (D) frontal pole synaptoneurosomes **Alcohol**  [^128^](#_ENREF_128) |  |
| **ANKRD28** Ankyrin Repeat Domain 28 | 229307_at | (I)  DE/6 80.5% | 0 | 6.20E-01/2 Stepwise | 15 |  | **Mood instability** [^189^](#_ENREF_189)  **BP**  [^190^](#_ENREF_190)  **Depression** Females [^52^](#_ENREF_52) | (I) DLPFC, Females **Suicide Completers** [^191^](#_ENREF_191)  (I) OFC,PFC,  **MDD, Male MDD** [^138^](#_ENREF_138)  (I) Brain, **Female Depression** [^151^](#_ENREF_151) | (I) Blood **PTSD Childhood Trauma** [^192^](#_ENREF_192)  (I) Blood **Early Life Stress** [^193^](#_ENREF_193)  (I) Blood **Alcohol** [^194^](#_ENREF_194)  (I) leukocytes **ASD** [^195^](#_ENREF_195) |  | (I) Microglia in PFC **Alcohol** [^196^](#_ENREF_196) |  |
| **CCKBR** Cholecystokinin B Receptor | 210381_s_at | (I)  DE/2 48% | 4 | 4.37E-01/2 Stepwise | 15 |  | **BP** [^197^](#_ENREF_197)  **BP** [^31^](#_ENREF_31)  **Suicide** [^198^](#_ENREF_198)  **MDD** 33558674 [^79^](#_ENREF_79) | (I) Cerebellum, PFC, cingulate gyrus **Suicide** [^199^](#_ENREF_199)  (I)  middle temporal gyrus corresponding to BA 21 (BA21) **MDD**  [^200^](#_ENREF_200)  (I)  Male BA25 **MDD** [^91^](#_ENREF_91)  (I) dorsolateral prefrontal cortex **SZ** [^201^](#_ENREF_201) |  |  | (I) AMY, Ventral Hippocampus **MDD** [^108^](#_ENREF_108)  (I) AMY **Alcohol** [^65^](#_ENREF_65)  (I) AMY (males)  **BP** [^202^](#_ENREF_202)  (I) NAC, Hippocampus **Phencyclidine**  [^104^](#_ENREF_104)  (I) PFC **Chronic Stress** [^203^](#_ENREF_203) |  |
| **DYNLL2** Dynein Light Chain LC8-Type 2 | 229106_at | (D)  DE/2 39.3% | 4 | 1.96E-01/2 Stepwise | 15 |  | **PTSD** 25754082 | (D) Forebrain neural progenitor cells  **SZ** [^204^](#_ENREF_204)  (D) Frontal Lobe **Alzheimer's Disease**[^115^](#_ENREF_115)  (D) Frontal cortex,Temporal lobe,Entorhinal cortex **Alzheimer's Disease** [^205^](#_ENREF_205)  (D) dorsolateral prefrontal cortex **SZ** [^201^](#_ENREF_201) |  |  | (D) Male NAC **Stress**[^91^](#_ENREF_91)  (D) AMY **Methamphetamine**[^206^](#_ENREF_206)  (D) Astrocytes in PFC **Alcohol** 29305589 [^207^](#_ENREF_207) | (D) skeletal muscle **Aging**  [^208^](#_ENREF_208) |
| **Hs.550187** | 240253_at | (I)  DE/6 88.3% | 0 | 2.74E-01/2 Stepwise | 15 |  |  |  |  |  |  |  |
| **NRG1** Neuregulin 1 | 208232_x_at | (I)  DE/4 65.8% | 4 | 6.66E-01/2 Stepwise | 15 |  | **SZ**  [^209^](#_ENREF_209)  **Methamphetamine** [^210^](#_ENREF_210)  **Longevity** [^131^](#_ENREF_131)  **Psychosis** [^211^](#_ENREF_211)  **SZ** [^212^](#_ENREF_212)  **BP** [^213^](#_ENREF_213)  **Suicide** [^76^](#_ENREF_76)  **MDD** [^214^](#_ENREF_214)  **Depression** [^215^](#_ENREF_215)  **BP time to recurrence**  [^216^](#_ENREF_216)  **SZA** [^217^](#_ENREF_217)  **Alcohol**  [^81^](#_ENREF_81)  **Depression Males and Females** [^52^](#_ENREF_52) | (I)  BA-9 PFC  **SZ** [^218^](#_ENREF_218),[^219^](#_ENREF_219)  (I)  CA3/2 Stratum oriens, **SZ** [^82^](#_ENREF_82)  (I) DLPFC **SZ** 14569272  (I) hippocampus  **SZ** [^220^](#_ENREF_220)  (I) PFC , **SZ** [^221^](#_ENREF_221)  [^222^](#_ENREF_222)  (I) postmortem brain tissue  **SZ** [^223^](#_ENREF_223)  (I)  **SZ** [^224^](#_ENREF_224) | (I) **Aging** [^145^](#_ENREF_145)  (I) PBMC **MDD** [^180^](#_ENREF_180)  (I) Leukocytes, **SZ** [^225^](#_ENREF_225)  (I) Blood **SZ**  [^226^](#_ENREF_226)  (I)  SH-SY5Y cells **Cocaine** [^227^](#_ENREF_227)  (I) Fibroblast  **SZ** [^228^](#_ENREF_228)  (I) Leucocytes **SZ** [^225^](#_ENREF_225)  (I) lymphocyte  **SZ** [^229^](#_ENREF_229)  (I) PBMC **BP** [^230^](#_ENREF_230)  (I) PBMC **MDD** [^231^](#_ENREF_231)  (I) PBMCs **Chronic Stress** [^232^](#_ENREF_232)  (I) blood  **MDD** [^233^](#_ENREF_233), [^234^](#_ENREF_234) [^235^](#_ENREF_235)  (I)  Lung and blood , **MDD ,SZ and COVID-19** [^103^](#_ENREF_103)  (I) Plasma **Alzheimer's Disease** [^236^](#_ENREF_236)  (I) PBMC **PTSD** [^237^](#_ENREF_237)  (I) Blood **MDD**  [^238^](#_ENREF_238) | **SZ** [^239^](#_ENREF_239), [^240^](#_ENREF_240), [^241^](#_ENREF_241), [^242^](#_ENREF_242), [^243^](#_ENREF_243) | (I) AMY **MDD** [^10^](#_ENREF_10)  (I) Hippocampus **PTSD** [^244^](#_ENREF_244)  (I) AMY **Chronic Stress** [^245^](#_ENREF_245)  (I) PFC **Memory, SZ** [^246^](#_ENREF_246)  (I) PFC **SZ** [^247^](#_ENREF_247) | (I) plasma **Aging** [^85^](#_ENREF_85) |
| **TFRC** Transferrin Receptor | 207332_s_at | (D) DE/4 56% | 2 | 7.11E-01/2 Stepwise | 15 |  | **Aging** [^248^](#_ENREF_248)  **BP**  [^190^](#_ENREF_190)  **SZ**  [^249^](#_ENREF_249) | (D) Frontal Pole (Ba 10)  **SZ** [^250^](#_ENREF_250)  (D) Frontal Cortex **ALZ** [^251^](#_ENREF_251)  (D) Hippocampus **ALZ** [^83^](#_ENREF_83)  (D) Hippocampus **Alcohol** [^122^](#_ENREF_122) | (D) SH-SY5Y cells **Cocaine** [^227^](#_ENREF_227)  (D) **Longevity** [^252^](#_ENREF_252)  (D) Peripheral whole blood **Recurrent MDD** [^253^](#_ENREF_253)  (D) plasma **Aging**  [^85^](#_ENREF_85) |  | (D) DRG **Neuropathic Pain**2[^254^](#_ENREF_254)  (D) CP **Alcohol** [^65^](#_ENREF_65)  (D) Brain**Depression SAD**[^92^](#_ENREF_92)  (D) CeA **Alcohol** [^93^](#_ENREF_93) |  |

**Table S4. Pharmacogenomics.** Top biomarkers (from Table 2) that are targets of existing drugs and are changed in expression in **opposite direction to high anxiety**. (I)- increased in expression, (D)- decreased in expression. DE- differential expression, AP-Absent/Present.

| **Gene Symbol/ Gene Name** | **Probeset** | **Step 1 Discovery (Change) Method/Score  6pts** | **Step 2 Prioritization Total CFG Score For Anxiety** | **Step 3**  **Validation Anova p-value  6 pts** | **CFE Polyevidence Score for Involvement in Anxiety (Based on Steps 1-4)** |  | **Omega-3 Fatty Acids** | **Antidepressants** | **Mood Stabilizers** | **Benzodiazapines** | **Other Treatments** |
| --- | --- | --- | --- | --- | --- | --- | --- | --- | --- | --- | --- |
| **GAD1** Glutamate Decarboxylase 1 | 205278_at | (I)  DE/4 62.3% | 11 | 6.79E-01/0 Not Stepwise | 22 |  | (D) Lymphocytes **Omega-3 fatty acids** [^255^](#_ENREF_255) |  | (D) neuroblastoma cells **Lithium,** **Valproate** [^256^](#_ENREF_256)  (D)  Embryonic Stem Cell **Carbamazepine**  [^257^](#_ENREF_257) |  |  |
| **NTRK3** Neurotrophic Receptor Tyrosine Kinase 3 | 215025_at | (I)  DE/2 35.1% | 4 | 6.58E-01/2 Stepwise | 19 |  |  |  |  |  | (D)  VT **Clozapine** [^104^](#_ENREF_104) |
| **ADRA2A** Adrenoceptor Alpha 2A | 209869_at | (I) AP/4 50% | 4 | 9.07E-01/0 Not Stepwise | 17 |  |  | (D)  larvae  **Norfluoxetine (SSRI)** [^258^](#_ENREF_258) | (D)  Embryonic Stem Cell  **Valproate**  [^257^](#_ENREF_257)  (D)  Embryonic Stem Cell  **Carbamazepine**  [^257^](#_ENREF_257) |  | (D)  VT **Clozapine** [^104^](#_ENREF_104) |
| **FZD10** Frizzled Class Receptor 10 | 219764_at | (I)  DE/4 53.2% | 2 | 3.57E-01/2 Stepwise | 17 |  |  | (D)  Cell Line **Fluoxetine** [^259^](#_ENREF_259) |  |  | (D)  NT2 human teratocarcinoma cells Cell Culture **Aripiprazole**  [^260^](#_ENREF_260)  (D) Hippocampus (CA1) **Gamma frequency** [^261^](#_ENREF_261) |
| **ATP1B2** ATPase Na+/K+ Transporting Subunit Beta 2 | 204311_at | (I)  DE/2 39% | 4 | 1.65E-01/2 Stepwise | 16 |  |  |  | (D)  AMY **Valproate** [^206^](#_ENREF_206)  (D)  **Lithium** [^262^](#_ENREF_262) |  | (D)  straiatum **Haloperidol** [^263^](#_ENREF_263)  (D) VT **Clozapine** [^104^](#_ENREF_104)  (D) Prefrontal cortex  **Digoxin** [^264^](#_ENREF_264) |
| **CLIC6** Chloride Intracellular Channel 6 | 242913_at | (I) AP/6 84.2% | 2 | 5.06E-01/0 Not Stepwise | 16 |  | (D) Lymphocytes (females)  **Omega-3 fatty acids** [^255^](#_ENREF_255) |  |  |  | (D)  AMY **Clozapine** [^104^](#_ENREF_104)  (D)  Hippocampus (CA1)  **Gamma frequency** [^261^](#_ENREF_261) |
| **EFNA5** Ephrin A5 | 1559360_at | (I)  DE/6 80.5% (I) AP/4 51.3% | 2 | 2.02E-04/4 Nominal | 16 |  | (D) Brain **Omega-3 fatty acids** [^265^](#_ENREF_265) |  |  |  | (D) Hippocampus (CA1) **Gamma frequency** [^261^](#_ENREF_261) |
| **GPX7** Glutathione Peroxidase 7 | 213170_at | (D) DE/4 64.3% | 2 | 4.64E-01/2 Stepwise | 16 |  |  | (I)  C.elegans **Mianserin** [^266^](#_ENREF_266) |  |  | (I)  PFC  **SAM** [^146^](#_ENREF_146) |
| **NTRK3**  Neurotrophic Receptor Tyrosine Kinase 3 | 215311_at | (I)  DE/4 53.2% | 4 | 3.26E-01/2  Stepwise | 16 |  |  |  |  |  | (D)  VT **Clozapine** [^104^](#_ENREF_104) |
| **SLC6A2** Solute Carrier Family 6 Member 2 | 217214_s_at | (I)  DE/2 40.3% | 4 | 3.40E-01/2 Stepwise | 16 |  |  | (D)  Cortex **Fluoxetine** [^267^](#_ENREF_267)  (D)  larvae **Norfluoxetine (SSRI)** [^258^](#_ENREF_258) |  |  |  |
| **SLC6A4**  Solute Carrier Family 6 Member 4 | 242009_at | (I)  AP/2  35.1% | 10 | 8.51E-01/0NS | 16 |  | (D) Lymphocytes **Omega-3 fatty acids** [^255^](#_ENREF_255) | (D)  Neural progenitor cells **imipramine, citalopram** [^268^](#_ENREF_268)  (D)  Blood **Vortioxetine** [^269^](#_ENREF_269)  (D)  Blood **Agomelatine** [^181^](#_ENREF_181)  (D)  DR **Sertraline**  [^270^](#_ENREF_270) |  |  | (D) Hippocampus  **Oxycodone** [^271^](#_ENREF_271) |
| **TMEM138** Transmembrane Protein 138 | 223113_at | (D)  DE/4 63% | 4 | 8.63E-01/0 Not Stepwise | 16 |  |  |  |  |  |  |
| **ANKRD28** Ankyrin Repeat Domain 28 | 229307_at | (I)  DE/6 80.5% | 0 | 6.20E-01/2 Stepwise | 15 |  |  |  |  |  |  |
| **CCKBR** Cholecystokinin B Receptor | 210381_s_at | (I)  DE/2 48% | 4 | 4.37E-01/2 Stepwise | 15 |  |  |  |  |  | (D)  VT **Clozapine** [^104^](#_ENREF_104) |
| **DYNLL2** Dynein Light Chain LC8-Type 2 | 229106_at | (D)  DE/2 39.3% | 4 | 1.96E-01/2 Stepwise | 15 |  |  |  | (I) Embryonic stem cell **Valproate** [^257^](#_ENREF_257) | (I)  AMY **Benzodiazepines** [^38^](#_ENREF_38) |  |
| **GRK4** G Protein-Coupled Receptor Kinase 4 | 210600_s_at | (I)  DE/6 81.8% | 0.00 | 3.40E-01/2 Stepwise | 15 |  |  |  |  |  | (D)  Blood **Clozapine** [^272^](#_ENREF_272)  (D) Hippocampus (CA1)  **Gamma frequency** [^261^](#_ENREF_261) |
| **Hs.550187** | 240253_at | (I)  DE/6 88.3% | 0 | 2.74E-01/2 Stepwise | 15 |  |  |  |  |  |  |
| **NRG1** Neuregulin 1 | 208232_x_at | (I)  DE/4 65.8% | 4 | 6.66E-01/2 Stepwise | 15 |  |  | (D) Hippocampus, PFC **Ketamine** [^273^](#_ENREF_273) | (D) neuroblastoma cells **Lithium Resistance** [^274^](#_ENREF_274)  (D) neuroblastoma cells **Valproate**[^256^](#_ENREF_256) |  | (D) blood **Antipsychotic response in men** [^275^](#_ENREF_275) |
| **TFRC** Transferrin Receptor | 207332_s_at | (D) DE/4 56% | 2 | 7.11E-01/2 Stepwise | 15 |  | (I)  PFC (males) **Omega-3 fatty acids** [^255^](#_ENREF_255)  (I) Lymphocytes  **Omega-3 fatty** **acids** [^255^](#_ENREF_255) | (I)  Dentate gyrus **Fluoxetine** [^276^](#_ENREF_276)  (I) basal forebrain **TCA**  [^43^](#_ENREF_43) | (I)  NT2.D1 cells **Valproate** [^277^](#_ENREF_277) |  | (I)  **Clozapine** [^278^](#_ENREF_278)  (I)  Mucosal Biopsy **Rofecoxib** [^279^](#_ENREF_279)  (I)  HCAEC **Berberine** [^280^](#_ENREF_280)  (I)  Whole Brains **Methylphenidate**  [^281^](#_ENREF_281) |

**Table S5 Biology of Anxiety Biomarkers. Top CFE3 =>8** (n= 95 probesets, 82 genes**).** A. Pathway Analyses B. Diseases C. Psychiatric co-morbidities

| **A.** | **KEGG Pathways** | | | | **Ingenuity Pathways** | | |
| --- | --- | --- | --- | --- | --- | --- | --- |
|  | **Term** | **Count** | **%** | **P-Value** | **Top Canonical Pathways** | **P-Value** | **Overlap** |
| **Top candidate biomarkers**  (n=  95 probesets, 82 genes) | Hippo Signaling Pathway | 6 | 7.4 | 1.60E-03 | **CREB Signaling in Neurons** | 4.56E-06 | 1.8 % 11/606 |
|  | Neuroactive Ligand-receptor Interaction | 8 | 9.9 | 3.40E-03 | Cardiac Hypertrophy Signaling (Enhanced) | 1.12E-05 | 1.8 % 10/542 |
|  | Proteoglycans in Cancer | 6 | 7.4 | 5.20E-03 | Relaxin Signaling | 1.23E-05 | 3.9 % 6/155 |
|  | Rap1 Signaling Pathway | 6 | 7.4 | 5.70E-03 | Molecular Mechanisms of Cancer | 1.57E-05 | 2.0 % 9/446 |
|  | **cAMP Signaling Pathway** | 6 | 7.4 | 7.10E-03 | G-Protein Coupled Receptor Signaling | 1.81E-05 | 1.6 % 11/702 |

| **B.** | **David** | | | | | **Ingenuity Pathways Disease** | | |
| --- | --- | --- | --- | --- | --- | --- | --- | --- |
| **Top candidate biomarkers**  (n=  95 probesets, 82 genes) | # | **Term** | **Count** | **%** | **P-Value** | **Diseases and Disorders** | **P-Value** | **# Molecules** |
|  | 1 | Depression | 11 | 13.6 | 5.90E-09 | Neurological Disease | 2.43E-04 - 7.96E-12 | 65 |
|  | 2 | Several Psychiatric disorders | 14 | 17.3 | 6.10E-09 | Organismal Injury and Abnormalities | 2.46E-04 - 7.96E-12 | 77 |
|  | 3 | Alcohol consumption | 10 | 12.3 | 3.10E-08 | Cancer | 2.45E-04 - 2.78E-10 | 77 |
|  | 4 | Attention deficit disorder conduct disorder oppositional defiant disorder | 7 | 8.6 | 6.60E-07 | Hematological Disease | 2.24E-04 - 2.78E-10 | 45 |
|  | 5 | Tourette Syndrome | 5 | 6.2 | 2.30E-06 | Immunological Disease | 1.64E-04 - 2.78E-10 | 49 |
|  | 6 | Panic Disorder | 6 | 7.4 | 5.20E-06 |  |  |  |
|  | 7 | Bulimia | 9 | 11.1 | 6.20E-06 |  |  |  |
|  | 8 | Schizophrenia | 14 | 17.3 | 8.40E-06 |  |  |  |
|  | 9 | Tobacco Use Disorder | 34 | 42 | 1.20E-05 |  |  |  |
|  | 10 | Bipolar Disorder | 8 | 9.9 | 1.30E-05 |  |  |  |

**C. Genomic co-morbidity for Anxiety** For Top Biomarkers from Table 2 (n= 19). See also table S3.

| **Co-morbidity** | **Percentile Match** |
| --- | --- |
| Depression | 83.33 |
| Alcoholism | 72.22 |
| Stress | 55.56 |
| Schizophrenia | 50.00 |
| Bipolar | 44.44 |
| Aging | 38.89 |
| Dementia | 38.89 |
| PTSD | 38.89 |
| Suicide | 38.89 |
| Pain | 27.78 |
| Phencyclidine | 27.78 |
| Cocaine | 16.67 |
| Cannabis | 11.11 |
| Mood | 11.11 |
| ASD | 5.56 |
| Fear | 5.56 |
| Memory | 5.56 |
| Methamphetamine | 5.56 |
| Morphine | 5.56 |
| Neurological | 5.56 |
| Schizoaffective | 5.56 |

**Literature Cited:**

1. Hettema JM, An SS, Neale MC, Bukszar J, van den Oord EJ, Kendler KS *et al.* Association between glutamic acid decarboxylase genes and anxiety disorders, major depression, and neuroticism. *Mol Psychiatry* 2006; **11**(8)**:** 752-762.

2. Piantadosi SC, Chamberlain BL, Glausier JR, Lewis DA, Ahmari SE. Lower excitatory synaptic gene expression in orbitofrontal cortex and striatum in an initial study of subjects with obsessive compulsive disorder. *Mol Psychiatry* 2019.

3. Domschke K, Tidow N, Schrempf M, Schwarte K, Klauke B, Reif A *et al.* Epigenetic signature of panic disorder: a role of glutamate decarboxylase 1 (GAD1) DNA hypomethylation? *Prog Neuropsychopharmacol Biol Psychiatry* 2013; **46:** 189-196.

4. Smith KM. Hyperactivity in mice lacking one allele of the glutamic acid decarboxylase 67 gene. *Atten Defic Hyperact Disord* 2018; **10**(4)**:** 267-271.

5. Zhu C, Liang M, Li Y, Feng X, Hong J, Zhou R. Involvement of Epigenetic Modifications of GABAergic Interneurons in Basolateral Amygdala in Anxiety-like Phenotype of Prenatally Stressed Mice. *Int J Neuropsychopharmacol* 2018; **21**(6)**:** 570-581.

6. Alonso P, Gratacos M, Menchon JM, Segalas C, Gonzalez JR, Labad J *et al.* Genetic susceptibility to obsessive-compulsive hoarding: the contribution of neurotrophic tyrosine kinase receptor type 3 gene. *Genes Brain Behav* 2008; **7**(7)**:** 778-785.

7. Muinos-Gimeno M, Guidi M, Kagerbauer B, Martin-Santos R, Navines R, Alonso P *et al.* Allele variants in functional MicroRNA target sites of the neurotrophin-3 receptor gene (NTRK3) as susceptibility factors for anxiety disorders. *Hum Mutat* 2009; **30**(7)**:** 1062-1071.

8. Santos M, D'Amico D, Spadoni O, Amador-Arjona A, Stork O, Dierssen M. Hippocampal hyperexcitability underlies enhanced fear memories in TgNTRK3, a panic disorder mouse model. *J Neurosci* 2013; **33**(38)**:** 15259-15271.

9. Dierssen M, Gratacos M, Sahun I, Martin M, Gallego X, Amador-Arjona A *et al.* Transgenic mice overexpressing the full-length neurotrophin receptor TrkC exhibit increased catecholaminergic neuron density in specific brain areas and increased anxiety-like behavior and panic reaction. *Neurobiol Dis* 2006; **24**(2)**:** 403-418.

10. Surget A, Wang Y, Leman S, Ibarguen-Vargas Y, Edgar N, Griebel G *et al.* Corticolimbic transcriptome changes are state-dependent and region-specific in a rodent model of depression and of antidepressant reversal. *Neuropsychopharmacology* 2009; **34**(6)**:** 1363-1380.

11. Rubin DH, Althoff RR, Ehli EA, Davies GE, Rettew DC, Crehan ET *et al.* Candidate gene associations with withdrawn behavior. *J Child Psychol Psychiatry* 2013; **54**(12)**:** 1337-1345.

12. Liu M, Fitzgibbon M, Wang Y, Reilly J, Qian X, O'Brien T *et al.* Ulk4 regulates GABAergic signaling and anxiety-related behavior. *Transl Psychiatry* 2018; **8**(1)**:** 43.

13. Caronia G, Wilcoxon J, Feldman P, Grove EA. Bone morphogenetic protein signaling in the developing telencephalon controls formation of the hippocampal dentate gyrus and modifies fear-related behavior. *J Neurosci* 2010; **30**(18)**:** 6291-6301.

14. Philibert RA, Crowe R, Ryu GY, Yoon JG, Secrest D, Sandhu H *et al.* Transcriptional profiling of lymphoblast lines from subjects with panic disorder. *Am J Med Genet B Neuropsychiatr Genet* 2007; **144B**(5)**:** 674-682.

15. Mehta NS, Wang L, Redei EE. Sex differences in depressive, anxious behaviors and hippocampal transcript levels in a genetic rat model. *Genes Brain Behav* 2013; **12**(7)**:** 695-704.

16. Virok DP, Kis Z, Szegedi V, Juhasz G, Zvara A, Jr., Muller G *et al.* Functional changes in transcriptomes of the prefrontal cortex and hippocampus in a mouse model of anxiety. *Pharmacol Rep* 2011; **63**(2)**:** 348-361.

17. Cittaro D, Lampis V, Luchetti A, Coccurello R, Guffanti A, Felsani A *et al.* Histone Modifications in a Mouse Model of Early Adversities and Panic Disorder: Role for Asic1 and Neurodevelopmental Genes. *Sci Rep* 2016; **6:** 25131.

18. Buttenschon HN, Kristensen AS, Buch HN, Andersen JH, Bonde JP, Grynderup M *et al.* The norepinephrine transporter gene is a candidate gene for panic disorder. *Journal of neural transmission* 2011; **118**(6)**:** 969-976.

19. Esler M, Alvarenga M, Pier C, Richards J, El-Osta A, Barton D *et al.* The neuronal noradrenaline transporter, anxiety and cardiovascular disease. *J Psychopharmacol* 2006; **20**(4 Suppl)**:** 60-66.

20. Forstner AJ, Rambau S, Friedrich N, Ludwig KU, Bohmer AC, Mangold E *et al.* Further evidence for genetic variation at the serotonin transporter gene SLC6A4 contributing toward anxiety. *Psychiatr Genet* 2017; **27**(3)**:** 96-102.

21. Wendland JR, Moya PR, Kruse MR, Ren-Patterson RF, Jensen CL, Timpano KR *et al.* A novel, putative gain-of-function haplotype at SLC6A4 associates with obsessive-compulsive disorder. *Hum Mol Genet* 2008; **17**(5)**:** 717-723.

22. Gyawali S, Subaran R, Weissman MM, Hershkowitz D, McKenna MC, Talati A *et al.* Association of a polyadenylation polymorphism in the serotonin transporter and panic disorder. *Biol Psychiatry* 2010; **67**(4)**:** 331-338.

23. Azadmarzabadi E, Haghighatfard A, Mohammadi A. Low resilience to stress is associated with candidate gene expression alterations in the dopaminergic signalling pathway. *Psychogeriatrics* 2018; **18**(3)**:** 190-201.

24. Roberts S, Lester KJ, Hudson JL, Rapee RM, Creswell C, Cooper PJ *et al.* Serotonin transporter [corrected] methylation and response to cognitive behaviour therapy in children with anxiety disorders. *Transl Psychiatry* 2014; **4:** e444.

25. Delorme R, Betancur C, Callebert J, Chabane N, Laplanche JL, Mouren-Simeoni MC *et al.* Platelet serotonergic markers as endophenotypes for obsessive-compulsive disorder. *Neuropsychopharmacology* 2005; **30**(8)**:** 1539-1547.

26. Hu XZ, Lipsky RH, Zhu G, Akhtar LA, Taubman J, Greenberg BD *et al.* Serotonin transporter promoter gain-of-function genotypes are linked to obsessive-compulsive disorder. *Am J Hum Genet* 2006; **78**(5)**:** 815-826.

27. Zhang S, Amstein T, Shen J, Brush FR, Gershenfeld HK. Molecular correlates of emotional learning using genetically selected rat lines. *Genes Brain Behav* 2005; **4**(2)**:** 99-109.

28. Line SJ, Barkus C, Coyle C, Jennings KA, Deacon RM, Lesch KP *et al.* Opposing alterations in anxiety and species-typical behaviours in serotonin transporter overexpressor and knockout mice. *Eur Neuropsychopharmacol* 2011; **21**(1)**:** 108-116.

29. de Almeida Magalhaes T, Correia D, de Carvalho LM, Damasceno S, Brunialti Godard AL. Maternal separation affects expression of stress response genes and increases vulnerability to ethanol consumption. *Brain Behav* 2018; **8**(1)**:** e00841.

30. Gratacos M, Costas J, de Cid R, Bayes M, Gonzalez JR, Baca-Garcia E *et al.* Identification of new putative susceptibility genes for several psychiatric disorders by association analysis of regulatory and non-synonymous SNPs of 306 genes involved in neurotransmission and neurodevelopment. *Am J Med Genet B Neuropsychiatr Genet* 2009; **150B**(6)**:** 808-816.

31. Wilson J, Markie D, Fitches A. Cholecystokinin system genes: associations with panic and other psychiatric disorders. *J Affect Disord* 2012; **136**(3)**:** 902-908.

32. Maron E, Nikopensius T, Koks S, Altmae S, Heinaste E, Vabrit K *et al.* Association study of 90 candidate gene polymorphisms in panic disorder. *Psychiatr Genet* 2005; **15**(1)**:** 17-24.

33. Hosing VG, Schirmacher A, Kuhlenbaumer G, Freitag C, Sand P, Schlesiger C *et al.* Cholecystokinin- and cholecystokinin-B-receptor gene polymorphisms in panic disorder. *J Neural Transm Suppl* 2004; (68)**:** 147-156.

34. Kennedy JL, Bradwejn J, Koszycki D, King N, Crowe R, Vincent J *et al.* Investigation of cholecystokinin system genes in panic disorder. *Mol Psychiatry* 1999; **4**(3)**:** 284-285.

35. Gelernter J, Bonvicini K, Page G, Woods SW, Goddard AW, Kruger S *et al.* Linkage genome scan for loci predisposing to panic disorder or agoraphobia. *Am J Med Genet* 2001; **105**(6)**:** 548-557.

36. Chen Q, Nakajima A, Meacham C, Tang YP. Elevated cholecystokininergic tone constitutes an important molecular/neuronal mechanism for the expression of anxiety in the mouse. *Proc Natl Acad Sci U S A* 2006; **103**(10)**:** 3881-3886.

37. Wang H, Spiess J, Wong PT, Zhu YZ. Blockade of CRF1 and CCK2 receptors attenuated the elevated anxiety-like behavior induced by immobilization stress. *Pharmacol Biochem Behav* 2011; **98**(3)**:** 362-368.

38. Le-Niculescu H, Balaraman Y, Patel SD, Ayalew M, Gupta J, Kuczenski R *et al.* Convergent functional genomics of anxiety disorders: translational identification of genes, biomarkers, pathways and mechanisms. *Transl Psychiatry* 2011; **1:** e9.

39. Donner J, Pirkola S, Silander K, Kananen L, Terwilliger JD, Lonnqvist J *et al.* An association analysis of murine anxiety genes in humans implicates novel candidate genes for anxiety disorders. *Biol Psychiatry* 2008; **64**(8)**:** 672-680.

40. Hovatta I, Tennant RS, Helton R, Marr RA, Singer O, Redwine JM *et al.* Glyoxalase 1 and glutathione reductase 1 regulate anxiety in mice. *Nature* 2005; **438**(7068)**:** 662-666.

41. Dina C, Nemanov L, Gritsenko I, Rosolio N, Osher Y, Heresco-Levy U *et al.* Fine mapping of a region on chromosome 8p gives evidence for a QTL contributing to individual differences in an anxiety-related personality trait: TPQ harm avoidance. *Am J Med Genet B Neuropsychiatr Genet* 2005; **132B**(1)**:** 104-108.

42. Chen YH, Lan YJ, Zhang SR, Li WP, Luo ZY, Lin S *et al.* ErbB4 signaling in the prelimbic cortex regulates fear expression. *Transl Psychiatry* 2017; **7**(7)**:** e1168.

43. Lagus M, Gass N, Saharinen J, Saarela J, Porkka-Heiskanen T, Paunio T. Gene expression patterns in a rodent model for depression. *Eur J Neurosci* 2010; **31**(8)**:** 1465-1473.

44. Kuo PH, Kalsi G, Prescott CA, Hodgkinson CA, Goldman D, Alexander J *et al.* Associations of glutamate decarboxylase genes with initial sensitivity and age-at-onset of alcohol dependence in the Irish Affected Sib Pair Study of Alcohol Dependence. *Drug Alcohol Depend* 2009; **101**(1-2)**:** 80-87.

45. Straub RE, Lipska BK, Egan MF, Goldberg TE, Callicott JH, Mayhew MB *et al.* Allelic variation in GAD1 (GAD67) is associated with schizophrenia and influences cortical function and gene expression. *Mol Psychiatry* 2007; **12**(9)**:** 854-869.

46. Lundorf MD, Buttenschon HN, Foldager L, Blackwood DH, Muir WJ, Murray V *et al.* Mutational screening and association study of glutamate decarboxylase 1 as a candidate susceptibility gene for bipolar affective disorder and schizophrenia. *Am J Med Genet B Neuropsychiatr Genet* 2005; **135B**(1)**:** 94-101.

47. Utge S, Soronen P, Partonen T, Loukola A, Kronholm E, Pirkola S *et al.* A population-based association study of candidate genes for depression and sleep disturbance. *Am J Med Genet B Neuropsychiatr Genet* 2010; **153B**(2)**:** 468-476.

48. Gandal MJ, Haney JR, Parikshak NN, Leppa V, Ramaswami G, Hartl C *et al.* Shared molecular neuropathology across major psychiatric disorders parallels polygenic overlap. *Science* 2018; **359**(6376)**:** 693-697.

49. Kochinke K, Zweier C, Nijhof B, Fenckova M, Cizek P, Honti F *et al.* Systematic Phenomics Analysis Deconvolutes Genes Mutated in Intellectual Disability into Biologically Coherent Modules. *Am J Hum Genet* 2016; **98**(1)**:** 149-164.

50. Haxhibeqiri S, Haxhibeqiri V, Agani F, Goci Uka A, Hoxha B, Dzubur Kulenovic A *et al.* Association of Neuropeptide S Receptor 1 and Glutamate Decarboxylase 1 Gene Polymorphisms with Posttraumatic Stress Disorder. *Psychiatr Danub* 2019; **31**(2)**:** 249-255.

51. Zwir I, Del-Val C, Arnedo J, Pulkki-Raback L, Konte B, Yang SS *et al.* Three genetic-environmental networks for human personality. *Mol Psychiatry* 2019.

52. Chan RF, Copeland WE, Zhao M, Xie LY, Costello J, Aberg KA *et al.* A methylation study implicates the rewiring of brain neural circuits during puberty in the emergence of sex differences in depression symptoms. *J Child Psychol Psychiatry* 2021.

53. Guillozet-Bongaarts AL, Hyde TM, Dalley RA, Hawrylycz MJ, Henry A, Hof PR *et al.* Altered gene expression in the dorsolateral prefrontal cortex of individuals with schizophrenia. *Mol Psychiatry* 2014; **19**(4)**:** 478-485.

54. Fillman SG, Sinclair D, Fung SJ, Webster MJ, Shannon Weickert C. Markers of inflammation and stress distinguish subsets of individuals with schizophrenia and bipolar disorder. *Transl Psychiatry* 2014; **4:** e365.

55. Bielau H, Steiner J, Mawrin C, Trubner K, Brisch R, Meyer-Lotz G *et al.* Dysregulation of GABAergic neurotransmission in mood disorders: a postmortem study. *Ann N Y Acad Sci* 2007; **1096:** 157-169.

56. Zhao J, Verwer RWH, Gao SF, Qi XR, Lucassen PJ, Kessels HW *et al.* Prefrontal alterations in GABAergic and glutamatergic gene expression in relation to depression and suicide. *J Psychiatr Res* 2018; **102:** 261-274.

57. Hakak Y, Walker JR, Li C, Wong WH, Davis KL, Buxbaum JD *et al.* Genome-wide expression analysis reveals dysregulation of myelination-related genes in chronic schizophrenia. *Proc Natl Acad Sci U S A* 2001; **98**(8)**:** 4746-4751.

58. Gos T, Gunther K, Bielau H, Dobrowolny H, Mawrin C, Trubner K *et al.* Suicide and depression in the quantitative analysis of glutamic acid decarboxylase-Immunoreactive neuropil. *J Affect Disord* 2009; **113**(1-2)**:** 45-55.

59. Cameron D, Blake DJ, Bray NJ, Hill MJ. Transcriptional Changes following Cellular Knockdown of the Schizophrenia Risk Gene SETD1A Are Enriched for Common Variant Association with the Disorder. *Mol Neuropsychiatry* 2019; **5**(2)**:** 109-114.

60. Yee JY, Nurjono M, Teo SR, Lee TS, Lee J. GAD1 Gene Expression in Blood of Patients with First-Episode Psychosis. *PLoS ONE* 2017; **12**(1)**:** e0170805.

61. Kim KH, Liu J, Sells Galvin RJ, Dage JL, Egeland JA, Smith RC *et al.* Transcriptomic Analysis of Induced Pluripotent Stem Cells Derived from Patients with Bipolar Disorder from an Old Order Amish Pedigree. *PLoS ONE* 2015; **10**(11)**:** e0142693.

62. Padmos RC, Bekris L, Knijff EM, Tiemeier H, Kupka RW, Cohen D *et al.* A high prevalence of organ-specific autoimmunity in patients with bipolar disorder. *Biol Psychiatry* 2004; **56**(7)**:** 476-482.

63. Sershen H, Guidotti A, Auta J, Drnevich J, Grayson DR, Veldic M *et al.* Gene Expression Of Methylation Cycle And Related Genes In Lymphocytes And Brain Of Patients With Schizophrenia And Non-Psychotic Controls. *Biomark Neuropsychiatry* 2021; **5**.

64. Conti B, Maier R, Barr AM, Morale MC, Lu X, Sanna PP *et al.* Region-specific transcriptional changes following the three antidepressant treatments electro convulsive therapy, sleep deprivation and fluoxetine. *Mol Psychiatry* 2007; **12**(2)**:** 167-189.

65. Rodd ZA, Bertsch BA, Strother WN, Le-Niculescu H, Balaraman Y, Hayden E *et al.* Candidate genes, pathways and mechanisms for alcoholism: an expanded convergent functional genomics approach. *Pharmacogenomics J* 2007; **7**(4)**:** 222-256.

66. Smith ML, Lopez MF, Wolen AR, Becker HC, Miles MF. Brain regional gene expression network analysis identifies unique interactions between chronic ethanol exposure and consumption. *PLoS ONE* 2020; **15**(5)**:** e0233319.

67. Nunez YO, Truitt JM, Gorini G, Ponomareva ON, Blednov YA, Harris RA *et al.* Positively correlated miRNA-mRNA regulatory networks in mouse frontal cortex during early stages of alcohol dependence. *BMC genomics* 2013; **14:** 725.

68. Freeman K, Staehle MM, Vadigepalli R, Gonye GE, Ogunnaike BA, Hoek JB *et al.* Coordinated dynamic gene expression changes in the central nucleus of the amygdala during alcohol withdrawal. *Alcohol Clin Exp Res* 2013; **37 Suppl 1:** E88-100.

69. Geng YM, Xue JT, Su JP, Li HY. Molecular mechanism of action of valproate acid alone or in combination with chlorpromazine in the epigenetic regulation of schizophrenia. *J Biol Regul Homeost Agents* 2018; **32**(6)**:** 1443-1450.

70. Buki A, Horvath G, Benedek G, Ducza E, Kekesi G. Impaired GAD1 expression in schizophrenia-related WISKET rat model with sex-dependent aggressive behavior and motivational deficit. *Genes Brain Behav* 2019; **18**(4)**:** e12507.

71. Shi J, Levinson DF, Duan J, Sanders AR, Zheng Y, Pe'er I *et al.* Common variants on chromosome 6p22.1 are associated with schizophrenia. *Nature* 2009; **460**(7256)**:** 753-757.

72. Kao CF, Chen HW, Chen HC, Yang JH, Huang MC, Chiu YH *et al.* Identification of Susceptible Loci and Enriched Pathways for Bipolar II Disorder Using Genome-Wide Association Studies. *Int J Neuropsychopharmacol* 2016; **19**(12).

73. Nurnberger JI, Jr., Koller DL, Jung J, Edenberg HJ, Foroud T, Guella I *et al.* Identification of pathways for bipolar disorder: a meta-analysis. *JAMA Psychiatry* 2014; **71**(6)**:** 657-664.

74. Verma R, Holmans P, Knowles JA, Grover D, Evgrafov OV, Crowe RR *et al.* Linkage disequilibrium mapping of a chromosome 15q25-26 major depression linkage region and sequencing of NTRK3. *Biol Psychiatry* 2008; **63**(12)**:** 1185-1189.

75. Feng Y, Vetro A, Kiss E, Kapornai K, Daroczi G, Mayer L *et al.* Association of the neurotrophic tyrosine kinase receptor 3 (NTRK3) gene and childhood-onset mood disorders. *Am J Psychiatry* 2008; **165**(5)**:** 610-616.

76. Sokolowski M, Wasserman J, Wasserman D. Polygenic associations of neurodevelopmental genes in suicide attempt. *Mol Psychiatry* 2016; **21**(10)**:** 1381-1390.

77. Goudriaan A, de Leeuw C, Ripke S, Hultman CM, Sklar P, Sullivan PF *et al.* Specific glial functions contribute to schizophrenia susceptibility. *Schizophr Bull* 2014; **40**(4)**:** 925-935.

78. Karlsson Linner R, Biroli P, Kong E, Meddens SFW, Wedow R, Fontana MA *et al.* Genome-wide association analyses of risk tolerance and risky behaviors in over 1 million individuals identify hundreds of loci and shared genetic influences. *Nat Genet* 2019; **51**(2)**:** 245-257.

79. Akula N, Marenco S, Johnson K, Feng N, Zhu K, Schulmann A *et al.* Deep transcriptome sequencing of subgenual anterior cingulate cortex reveals cross-diagnostic and diagnosis-specific RNA expression changes in major psychiatric disorders. *Neuropsychopharmacology* 2021; **46**(7)**:** 1364-1372.

80. Ryu S, Han J, Norden-Krichmar TM, Zhang Q, Lee S, Zhang Z *et al.* Genetic signature of human longevity in PKC and NF-kappaB signaling. *Aging cell* 2021; **20**(7)**:** e13362.

81. Lydall GJ, Bass NJ, McQuillin A, Lawrence J, Anjorin A, Kandaswamy R *et al.* Confirmation of prior evidence of genetic susceptibility to alcoholism in a genome-wide association study of comorbid alcoholism and bipolar disorder. *Psychiatr Genet* 2011; **21**(6)**:** 294-306.

82. Benes FM, Lim B, Subburaju S. Site-specific regulation of cell cycle and DNA repair in post-mitotic GABA cells in schizophrenic versus bipolars. *Proc Natl Acad Sci U S A* 2009; **106**(28)**:** 11731-11736.

83. Blalock EM, Geddes JW, Chen KC, Porter NM, Markesbery WR, Landfield PW. Incipient Alzheimer's disease: microarray correlation analyses reveal major transcriptional and tumor suppressor responses. *Proc Natl Acad Sci U S A* 2004; **101**(7)**:** 2173-2178.

84. Canli T, Wen R, Wang X, Mikhailik A, Yu L, Fleischman D *et al.* Differential transcriptome expression in human nucleus accumbens as a function of loneliness. *Mol Psychiatry* 2017; **22**(7)**:** 1069-1078.

85. Lehallier B, Gate D, Schaum N, Nanasi T, Lee SE, Yousef H *et al.* Undulating changes in human plasma proteome profiles across the lifespan. *Nat Med* 2019; **25**(12)**:** 1843-1850.

86. Bainomugisa CK, Sutherland HG, Parker R, McRae AF, Haupt LM, Griffiths LR *et al.* Using Monozygotic Twins to Dissect Common Genes in Posttraumatic Stress Disorder and Migraine. *Frontiers in neuroscience* 2021; **15:** 678350.

87. Hu G, Yu S, Yuan C, Hong W, Wang Z, Zhang R *et al.* Gene expression signatures differentiating major depressive disorder from subsyndromal symptomatic depression. *Aging (Albany NY)* 2021; **13**(9)**:** 13124-13137.

88. Forstner AJ, Basmanav FB, Mattheisen M, Bohmer AC, Hollegaard MV, Janson E *et al.* Investigation of the involvement of MIR185 and its target genes in the development of schizophrenia. *J Psychiatry Neurosci* 2014; **39**(6)**:** 386-396.

89. McBride WJ, Kimpel MW, Schultz JA, McClintick JN, Edenberg HJ, Bell RL. Changes in gene expression in regions of the extended amygdala of alcohol-preferring rats after binge-like alcohol drinking. *Alcohol* 2010; **44**(2)**:** 171-183.

90. Malki K, Keers R, Tosto MG, Lourdusamy A, Carboni L, Domenici E *et al.* The endogenous and reactive depression subtypes revisited: integrative animal and human studies implicate multiple distinct molecular mechanisms underlying major depressive disorder. *BMC Med* 2014; **12:** 73.

91. Labonte B, Engmann O, Purushothaman I, Menard C, Wang J, Tan C *et al.* Sex-specific transcriptional signatures in human depression. *Nat Med* 2017; **23**(9)**:** 1102-1111.

92. Nakayama T, Okimura K, Shen J, Guh YJ, Tamai TK, Shimada A *et al.* Seasonal changes in NRF2 antioxidant pathway regulates winter depression-like behavior. *Proc Natl Acad Sci U S A* 2020; **117**(17)**:** 9594-9603.

93. Warden AS, Wolfe SA, Khom S, Varodayan FP, Patel RR, Steinman MQ *et al.* Microglia Control Escalation of Drinking in Alcohol-Dependent Mice: Genomic and Synaptic Drivers. *Biol Psychiatry* 2020; **88**(12)**:** 910-921.

94. Sandercock DA, Barnett MW, Coe JE, Downing AC, Nirmal AJ, Di Giminiani P *et al.* Transcriptomics Analysis of Porcine Caudal Dorsal Root Ganglia in Tail Amputated Pigs Shows Long-Term Effects on Many Pain-Associated Genes. *Front Vet Sci* 2019; **6:** 314.

95. Clarke TK, Dempster E, Docherty SJ, Desrivieres S, Lourdsamy A, Wodarz N *et al.* Multiple polymorphisms in genes of the adrenergic stress system confer vulnerability to alcohol abuse. *Addict Biol* 2012; **17**(1)**:** 202-208.

96. Haefner S, Baghai TC, Schule C, Eser D, Spraul M, Zill P *et al.* Impact of gene-gender effects of adrenergic polymorphisms on hypothalamic-pituitary-adrenal axis activity in depressed patients. *Neuropsychobiology* 2008; **58**(3-4)**:** 154-162.

97. Linnstaedt SD, Walker MG, Riker KD, Nyland JE, Hu J, Rossi C *et al.* Genetic variant rs3750625 in the 3'UTR of ADRA2A affects stress-dependent acute pain severity after trauma and alters a microRNA-34a regulatory site. *Pain* 2017; **158**(2)**:** 230-239.

98. Sequeira A, Mamdani F, Lalovic A, Anguelova M, Lesage A, Seguin M *et al.* Alpha 2A adrenergic receptor gene and suicide. *Psychiatry Res* 2004; **125**(2)**:** 87-93.

99. Gonzalez-Maeso J, Rodriguez-Puertas R, Meana JJ, Garcia-Sevilla JA, Guimon J. Neurotransmitter receptor-mediated activation of G-proteins in brains of suicide victims with mood disorders: selective supersensitivity of alpha(2A)-adrenoceptors. *Mol Psychiatry* 2002; **7**(7)**:** 755-767.

100. Garcia-Sevilla JA, Escriba PV, Ozaita A, La Harpe R, Walzer C, Eytan A *et al.* Up-regulation of immunolabeled alpha2A-adrenoceptors, Gi coupling proteins, and regulatory receptor kinases in the prefrontal cortex of depressed suicides. *J Neurochem* 1999; **72**(1)**:** 282-291.

101. Escriba PV, Ozaita A, Garcia-Sevilla JA. Increased mRNA expression of alpha2A-adrenoceptors, serotonin receptors and mu-opioid receptors in the brains of suicide victims. *Neuropsychopharmacology* 2004; **29**(8)**:** 1512-1521.

102. Breen MS, Tylee DS, Maihofer AX, Neylan TC, Mehta D, Binder EB *et al.* PTSD Blood Transcriptome Mega-Analysis: Shared Inflammatory Pathways across Biological Sex and Modes of Trauma. *Neuropsychopharmacology* 2018; **43**(3)**:** 469-481.

103. Quincozes-Santos A, Rosa RL, Tureta EF, Bobermin LD, Berger M, Guimaraes JA *et al.* COVID-19 impacts the expression of molecular markers associated with neuropsychiatric disorders. *Brain Behav Immun Health* 2021; **11:** 100196.

104. Le-Niculescu H, Balaraman Y, Patel S, Tan J, Sidhu K, Jerome RE *et al.* Towards understanding the schizophrenia code: an expanded convergent functional genomics approach. *Am J Med Genet B Neuropsychiatr Genet* 2007; **144B**(2)**:** 129-158.

105. Xu W, Liyanage VRB, MacAulay A, Levy RD, Curtis K, Olson CO *et al.* Genome-Wide Transcriptome Landscape of Embryonic Brain-Derived Neural Stem Cells Exposed to Alcohol with Strain-Specific Cross-Examination in BL6 and CD1 Mice. *Sci Rep* 2019; **9**(1)**:** 206.

106. Zhang EE, Liu AC, Hirota T, Miraglia LJ, Welch G, Pongsawakul PY *et al.* A genome-wide RNAi screen for modifiers of the circadian clock in human cells. *Cell* 2009; **139**(1)**:** 199-210.

107. McCarthy MJ, Welsh DK. Cellular circadian clocks in mood disorders. *J Biol Rhythms* 2012; **27**(5)**:** 339-352.

108. Bagot RC, Cates HM, Purushothaman I, Lorsch ZS, Walker DM, Wang J *et al.* Circuit-wide Transcriptional Profiling Reveals Brain Region-Specific Gene Networks Regulating Depression Susceptibility. *Neuron* 2016; **90**(5)**:** 969-983.

109. Hill WD, Davies NM, Ritchie SJ, Skene NG, Bryois J, Bell S *et al.* Genome-wide analysis identifies molecular systems and 149 genetic loci associated with income. *Nature communications* 2019; **10**(1)**:** 5741.

110. Gaine ME, Seifuddin F, Sabunciyan S, Lee RS, Benke KS, Monson ET *et al.* Differentially methylated regions in bipolar disorder and suicide. *Am J Med Genet B Neuropsychiatr Genet* 2019; **180**(7)**:** 496-507.

111. Martin C, Cho YE, Kim H, Yun S, Kanefsky R, Lee H *et al.* Altered DNA Methylation Patterns Associated With Clinically Relevant Increases in PTSD Symptoms and PTSD Symptom Profiles in Military Personnel. *Biol Res Nurs* 2018; **20**(3)**:** 352-358.

112. Clark SL, Chan R, Zhao M, Xie LY, Copeland WE, Aberg KA *et al.* Methylomic Investigation of Problematic Adolescent Cannabis Use and Its Negative Mental Health Consequences. *J Am Acad Child Adolesc Psychiatry* 2021.

113. Forero DA, Guio-Vega GP, Gonzalez-Giraldo Y. A comprehensive regional analysis of genome-wide expression profiles for major depressive disorder. *J Affect Disord* 2017; **218:** 86-92.

114. Sequeira A, Klempan T, Canetti L, ffrench-Mullen J, Benkelfat C, Rouleau GA *et al.* Patterns of gene expression in the limbic system of suicides with and without major depression. *Mol Psychiatry* 2007; **12**(7)**:** 640-655.

115. Patel H, Dobson RJB, Newhouse SJ. A Meta-Analysis of Alzheimer's Disease Brain Transcriptomic Data. *J Alzheimers Dis* 2019; **68**(4)**:** 1635-1656.

116. Lanz TA, Reinhart V, Sheehan MJ, Rizzo SJS, Bove SE, James LC *et al.* Postmortem transcriptional profiling reveals widespread increase in inflammation in schizophrenia: a comparison of prefrontal cortex, striatum, and hippocampus among matched tetrads of controls with subjects diagnosed with schizophrenia, bipolar or major depressive disorder. *Transl Psychiatry* 2019; **9**(1)**:** 151.

117. Cabrera B, Monroy-Jaramillo N, Fries GR, Mendoza-Morales RC, Garcia-Dolores F, Mendoza-Larios A *et al.* Brain Gene Expression Pattern of Subjects with Completed Suicide and Comorbid Substance Use Disorder. *Mol Neuropsychiatry* 2019; **5**(1)**:** 60-73.

118. Murano T, Hagihara H, Tajinda K, Matsumoto M, Miyakawa T. Transcriptomic immaturity inducible by neural hyperexcitation is shared by multiple neuropsychiatric disorders. *Commun Biol* 2019; **2:** 32.

119. Bhasin MK, Dusek JA, Chang BH, Joseph MG, Denninger JW, Fricchione GL *et al.* Relaxation response induces temporal transcriptome changes in energy metabolism, insulin secretion and inflammatory pathways. *PLoS ONE* 2013; **8**(5)**:** e62817.

120. McClintick JN, Thapa K, Liu Y, Xuei X, Edenberg HJ. Effects of chronic intermittent ethanol exposure and withdrawal on neuroblastoma cell transcriptome. *Alcohol* 2020; **85:** 119-126.

121. Daskalakis NP, Cohen H, Cai G, Buxbaum JD, Yehuda R. Expression profiling associates blood and brain glucocorticoid receptor signaling with trauma-related individual differences in both sexes. *Proc Natl Acad Sci U S A* 2014; **111**(37)**:** 13529-13534.

122. Farris SP, Harris RA, Ponomarev I. Epigenetic modulation of brain gene networks for cocaine and alcohol abuse. *Frontiers in neuroscience* 2015; **9:** 176.

123. Glavan D, Gheorman V, Gresita A, Hermann DM, Udristoiu I, Popa-Wagner A. Identification of transcriptome alterations in the prefrontal cortex, hippocampus, amygdala and hippocampus of suicide victims. *Sci Rep* 2021; **11**(1)**:** 18853.

124. Harris SE, Riggio V, Evenden L, Gilchrist T, McCafferty S, Murphy L *et al.* Age-related gene expression changes, and transcriptome wide association study of physical and cognitive aging traits, in the Lothian Birth Cohort 1936. *Aging (Albany NY)* 2017; **9**(12)**:** 2489-2503.

125. McClintick JN, Tischfield JA, Deng L, Kapoor M, Xuei X, Edenberg HJ. Ethanol activates immune response in lymphoblastoid cells. *Alcohol* 2019; **79:** 81-91.

126. Zubenko GS, Hughes HB, 3rd, Jordan RM, Lyons-Weiler J, Cohen BM. Differential hippocampal gene expression and pathway analysis in an etiology-based mouse model of major depressive disorder. *Am J Med Genet B Neuropsychiatr Genet* 2014; **165B**(6)**:** 457-466.

127. Sannino G, Pasqualini L, Ricciardelli E, Montilla P, Soverchia L, Ruggeri B *et al.* Acute stress enhances the expression of neuroprotection- and neurogenesis-associated genes in the hippocampus of a mouse restraint model. *Oncotarget* 2016; **7**(8)**:** 8455-8465.

128. O'Brien MA, Weston RM, Sheth NU, Bradley S, Bigbee J, Pandey A *et al.* Ethanol-Induced Behavioral Sensitization Alters the Synaptic Transcriptome and Exon Utilization in DBA/2J Mice. *Frontiers in genetics* 2018; **9:** 402.

129. Jansen PR, Watanabe K, Stringer S, Skene N, Bryois J, Hammerschlag AR *et al.* Genome-wide analysis of insomnia in 1,331,010 individuals identifies new risk loci and functional pathways. *Nat Genet* 2019; **51**(3)**:** 394-403.

130. Johnson C, Drgon T, Liu QR, Walther D, Edenberg H, Rice J *et al.* Pooled association genome scanning for alcohol dependence using 104,268 SNPs: validation and use to identify alcoholism vulnerability loci in unrelated individuals from the collaborative study on the genetics of alcoholism. *Am J Med Genet B Neuropsychiatr Genet* 2006; **141B**(8)**:** 844-853.

131. Levine ME, Crimmins EM. A Genetic Network Associated With Stress Resistance, Longevity, and Cancer in Humans. *J Gerontol A Biol Sci Med Sci* 2016; **71**(6)**:** 703-712.

132. Schlauch KA, Khaiboullina SF, De Meirleir KL, Rawat S, Petereit J, Rizvanov AA *et al.* Genome-wide association analysis identifies genetic variations in subjects with myalgic encephalomyelitis/chronic fatigue syndrome. *Transl Psychiatry* 2016; **6:** e730.

133. Davies G, Lam M, Harris SE, Trampush JW, Luciano M, Hill WD *et al.* Study of 300,486 individuals identifies 148 independent genetic loci influencing general cognitive function. *Nat Commun* 2018; **9**(1)**:** 2098.

134. Potkin SG, Guffanti G, Lakatos A, Turner JA, Kruggel F, Fallon JH *et al.* Hippocampal atrophy as a quantitative trait in a genome-wide association study identifying novel susceptibility genes for Alzheimer's disease. *PLoS One* 2009; **4**(8)**:** e6501.

135. Baum AE, Akula N, Cabanero M, Cardona I, Corona W, Klemens B *et al.* A genome-wide association study implicates diacylglycerol kinase eta (DGKH) and several other genes in the etiology of bipolar disorder. *Mol Psychiatry* 2008; **13**(2)**:** 197-207.

136. Mills MC, Tropf FC, Brazel DM, van Zuydam N, Vaez A, e QC *et al.* Identification of 371 genetic variants for age at first sex and birth linked to externalising behaviour. *Nat Hum Behav* 2021; **5**(12)**:** 1717-1730.

137. Kim KS, Pae CU, Chae JH, Bahk WM, Jun TY, Kim DJ *et al.* Effects of olanzapine on prolactin levels of female patients with schizophrenia treated with risperidone. *J Clin Psychiatry* 2002; **63**(5)**:** 408-413.

138. Girgenti MJ, Wang J, Ji D, Cruz DA, Traumatic Stress Brain Research G, Stein MB *et al.* Transcriptomic organization of the human brain in post-traumatic stress disorder. *Nat Neurosci* 2021; **24**(1)**:** 24-33.

139. Brucker K, Duggan C, Niezer J, Roseberry K, Le-Niculescu H, Niculescu AB *et al.* Assessing Risk of Future Suicidality in Emergency Department Patients. *Acad Emerg Med* 2019; **26**(4)**:** 376-383.

140. Woods NB, Parker AS, Moraghebi R, Lutz MK, Firth AL, Brennand KJ *et al.* Brief report: efficient generation of hematopoietic precursors and progenitors from human pluripotent stem cell lines. *Stem Cells* 2011; **29**(7)**:** 1158-1164.

141. Bernstein AI, Lin Y, Street RC, Lin L, Dai Q, Yu L *et al.* 5-Hydroxymethylation-associated epigenetic modifiers of Alzheimer's disease modulate Tau-induced neurotoxicity. *Hum Mol Genet* 2016; **25**(12)**:** 2437-2450.

142. Rocha NKR, Themoteo R, Brentani H, Forlenza OV, De Paula VJR. Neuronal-Glial Interaction in a Triple-Transgenic Mouse Model of Alzheimer's Disease: Gene Ontology and Lithium Pathways. *Frontiers in neuroscience* 2020; **14:** 579984.

143. Liu SX, Gades MS, Swain Y, Ramakrishnan A, Harris AC, Tran PV *et al.* Repeated morphine exposure activates synaptogenesis and other neuroplasticity-related gene networks in the dorsomedial prefrontal cortex of male and female rats. *Drug Alcohol Depend* 2021; **221:** 108598.

144. Gottschalk MG, Wesseling H, Guest PC, Bahn S. Proteomic enrichment analysis of psychotic and affective disorders reveals common signatures in presynaptic glutamatergic signaling and energy metabolism. *Int J Neuropsychopharmacol* 2014; **18**(2).

145. Peters MJ, Joehanes R, Pilling LC, Schurmann C, Conneely KN, Powell J *et al.* The transcriptional landscape of age in human peripheral blood. *Nature communications* 2015; **6:** 8570.

146. Topham L, Gregoire S, Kang H, Salmon-Divon M, Lax E, Millecamps M *et al.* The methyl donor S-adenosyl methionine reverses the DNA methylation signature of chronic neuropathic pain in mouse frontal cortex. *Pain Rep* 2021; **6**(2)**:** e944.

147. Lewis CM, Ng MY, Butler AW, Cohen-Woods S, Uher R, Pirlo K *et al.* Genome-wide association study of major recurrent depression in the U.K. population. *Am J Psychiatry* 2010; **167**(8)**:** 949-957.

148. Bosker FJ, Hartman CA, Nolte IM, Prins BP, Terpstra P, Posthuma D *et al.* Poor replication of candidate genes for major depressive disorder using genome-wide association data. *Mol Psychiatry* 2011; **16**(5)**:** 516-532.

149. Kim YK, Hwang JA, Lee HJ, Yoon HK, Ko YH, Lee BH *et al.* Association between norepinephrine transporter gene (SLC6A2) polymorphisms and suicide in patients with major depressive disorder. *J Affect Disord* 2014; **158:** 127-132.

150. Zhang K, Qu S, Chang S, Li G, Cao C, Fang K *et al.* An overview of posttraumatic stress disorder genetic studies by analyzing and integrating genetic data into genetic database PTSDgene. *Neurosci Biobehav Rev* 2017; **83:** 647-656.

151. Gammie SC. Creation of a gene expression portrait of depression and its application for identifying potential treatments. *Sci Rep* 2021; **11**(1)**:** 3829.

152. Thibault C, Lai C, Wilke N, Duong B, Olive MF, Rahman S *et al.* Expression profiling of neural cells reveals specific patterns of ethanol-responsive gene expression. *Mol Pharmacol* 2000; **58**(6)**:** 1593-1600.

153. Finn DA, Hashimoto JG, Cozzoli DK, Helms ML, Nipper MA, Kaufman MN *et al.* Binge Ethanol Drinking Produces Sexually Divergent and Distinct Changes in Nucleus Accumbens Signaling Cascades and Pathways in Adult C57BL/6J Mice. *Frontiers in genetics* 2018; **9:** 325.

154. Yun H, Park ES, Choi S, Shin B, Yu J, Yu J *et al.* TDAG51 is a crucial regulator of maternal care and depressive-like behavior after parturition. *PLoS Genet* 2019; **15**(6)**:** e1008214.

155. Feinn R, Nellissery M, Kranzler HR. Meta-analysis of the association of a functional serotonin transporter promoter polymorphism with alcohol dependence. *Am J Med Genet B Neuropsychiatr Genet* 2005; **133B**(1)**:** 79-84.

156. Druley TE, Wang L, Lin SJ, Lee JH, Zhang Q, Daw EW *et al.* Candidate gene resequencing to identify rare, pedigree-specific variants influencing healthy aging phenotypes in the long life family study. *BMC Geriatr* 2016; **16:** 80.

157. Li P, Liu T, Liu J, Zhang Q, Lou F, Kong F *et al.* Promoter polymorphism in the serotonin transporter (5-HTT) gene is significantly associated with leukocyte telomere length in Han Chinese. *PLoS One* 2014; **9**(4)**:** e94442.

158. Kistner-Griffin E, Brune CW, Davis LK, Sutcliffe JS, Cox NJ, Cook EH, Jr. Parent-of-origin effects of the serotonin transporter gene associated with autism. *Am J Med Genet B Neuropsychiatr Genet* 2011; **156**(2)**:** 139-144.

159. Zaboli G, Jonsson EG, Gizatullin R, De Franciscis A, Asberg M, Leopardi R. Haplotype analysis confirms association of the serotonin transporter (5-HTT) gene with schizophrenia but not with major depression. *Am J Med Genet B Neuropsychiatr Genet* 2008; **147**(3)**:** 301-307.

160. Neves FS, Silveira G, Romano-Silva MA, Malloy-Diniz L, Ferreira AA, De Marco L *et al.* Is the 5-HTTLPR polymorphism associated with bipolar disorder or with suicidal behavior of bipolar disorder patients? *Am J Med Genet B Neuropsychiatr Genet* 2008; **147B**(1)**:** 114-116.

161. Luykx JJ, Bakker SC, van Geloven N, Eijkemans MJ, Horvath S, Lentjes E *et al.* Seasonal variation of serotonin turnover in human cerebrospinal fluid, depressive symptoms and the role of the 5-HTTLPR. *Transl Psychiatry* 2013; **3:** e311.

162. Lopez-Leon S, Janssens AC, Gonzalez-Zuloeta Ladd AM, Del-Favero J, Claes SJ, Oostra BA *et al.* Meta-analyses of genetic studies on major depressive disorder. *Mol Psychiatry* 2008; **13**(8)**:** 772-785.

163. Collier DA, Stober G, Li T, Heils A, Catalano M, Di Bella D *et al.* A novel functional polymorphism within the promoter of the serotonin transporter gene: possible role in susceptibility to affective disorders. *Mol Psychiatry* 1996; **1**(6)**:** 453-460.

164. Brezo J, Bureau A, Merette C, Jomphe V, Barker ED, Vitaro F *et al.* Differences and similarities in the serotonergic diathesis for suicide attempts and mood disorders: a 22-year longitudinal gene-environment study. *Mol Psychiatry* 2010; **15**(8)**:** 831-843.

165. Vassos E, Collier DA, Fazel S. Systematic meta-analyses and field synopsis of genetic association studies of violence and aggression. *Mol Psychiatry* 2014; **19**(4)**:** 471-477.

166. Perroud N, Salzmann A, Saiz PA, Baca-Garcia E, Sarchiapone M, Garcia-Portilla MP *et al.* Rare genotype combination of the serotonin transporter gene associated with treatment response in severe personality disorder. *Am J Med Genet B Neuropsychiatr Genet* 2010; **153B**(8)**:** 1494-1497.

167. Sen S, Burmeister M, Ghosh D. Meta-analysis of the association between a serotonin transporter promoter polymorphism (5-HTTLPR) and anxiety-related personality traits. *Am J Med Genet B Neuropsychiatr Genet* 2004; **127B**(1)**:** 85-89.

168. Offenbaecher M, Bondy B, de Jonge S, Glatzeder K, Kruger M, Schoeps P *et al.* Possible association of fibromyalgia with a polymorphism in the serotonin transporter gene regulatory region. *Arthritis Rheum* 1999; **42**(11)**:** 2482-2488.

169. Cui W, Yu X, Zhang H. The serotonin transporter gene polymorphism is associated with the susceptibility and the pain severity in idiopathic trigeminal neuralgia patients. *J Headache Pain* 2014; **15:** 42.

170. Tour J, Lofgren M, Mannerkorpi K, Gerdle B, Larsson A, Palstam A *et al.* Gene-to-gene interactions regulate endogenous pain modulation in fibromyalgia patients and healthy controls-antagonistic effects between opioid and serotonin-related genes. *Pain* 2017; **158**(7)**:** 1194-1203.

171. James S. Human pain and genetics: some basics. *Br J Pain* 2013; **7**(4)**:** 171-178.

172. Kilpatrick DG, Koenen KC, Ruggiero KJ, Acierno R, Galea S, Resnick HS *et al.* The serotonin transporter genotype and social support and moderation of posttraumatic stress disorder and depression in hurricane-exposed adults. *Am J Psychiatry* 2007; **164**(11)**:** 1693-1699.

173. Caspi A, Sugden K, Moffitt TE, Taylor A, Craig IW, Harrington H *et al.* Influence of life stress on depression: moderation by a polymorphism in the 5-HTT gene. *Science* 2003; **301**(5631)**:** 386-389.

174. Spijker S, Van Zanten JS, De Jong S, Penninx BW, van Dyck R, Zitman FG *et al.* Stimulated gene expression profiles as a blood marker of major depressive disorder. *Biol Psychiatry* 2010; **68**(2)**:** 179-186.

175. Anmella G, Vilches S, Espadaler-Mazo J, Murru A, Pacchiarotti I, Tuson M *et al.* Genetic Variations Associated with Long-Term Treatment Response in Bipolar Depression. *Genes (Basel)* 2021; **12**(8).

176. Mullins N, Kang J, Campos AI, Coleman JRI, Edwards AC, Galfalvy H *et al.* Dissecting the Shared Genetic Architecture of Suicide Attempt, Psychiatric Disorders, and Known Risk Factors. *Biol Psychiatry* 2022; **91**(3)**:** 313-327.

177. Gross-Isseroff R, Israeli M, Biegon A. Autoradiographic analysis of tritiated imipramine binding in the human brain post mortem: effects of suicide. *Arch Gen Psychiatry* 1989; **46**(3)**:** 237-241.

178. Peng H, Zhu Y, Strachan E, Fowler E, Bacus T, Roy-Byrne P *et al.* Childhood Trauma, DNA Methylation of Stress-Related Genes, and Depression: Findings From Two Monozygotic Twin Studies. *Psychosom Med* 2018; **80**(7)**:** 599-608.

179. Ponder KL, Salisbury A, McGonnigal B, Laliberte A, Lester B, Padbury JF. Maternal depression and anxiety are associated with altered gene expression in the human placenta without modification by antidepressant use: implications for fetal programming. *Dev Psychobiol* 2011; **53**(7)**:** 711-723.

180. Belzeaux R, Formisano-Treziny C, Loundou A, Boyer L, Gabert J, Samuelian JC *et al.* Clinical variations modulate patterns of gene expression and define blood biomarkers in major depression. *J Psychiatr Res* 2010; **44**(16)**:** 1205-1213.

181. Belzeaux R, Azorin JM, Ibrahim EC. Monitoring candidate gene expression variations before, during and after a first major depressive episode in a 51-year-old man. *BMC Psychiatry* 2014; **14:** 73.

182. Seneviratne C, Johnson BA. Serotonin transporter genomic biomarker for quantitative assessment of ondansetron treatment response in alcoholics. *Front Psychiatry* 2012; **3:** 23.

183. Jones KL, Smith RM, Edwards KS, Givens B, Tilley MR, Beversdorf DQ. Combined effect of maternal serotonin transporter genotype and prenatal stress in modulating offspring social interaction in mice. *Int J Dev Neurosci* 2010; **28**(6)**:** 529-536.

184. Lo CL, Lossie AC, Liang T, Liu Y, Xuei X, Lumeng L *et al.* High Resolution Genomic Scans Reveal Genetic Architecture Controlling Alcohol Preference in Bidirectionally Selected Rat Model. *PLoS Genet* 2016; **12**(8)**:** e1006178.

185. Park H, Yoo D, Kwon S, Yoo TW, Park HJ, Hahm DH *et al.* Acupuncture stimulation at HT7 alleviates depression-induced behavioral changes via regulation of the serotonin system in the prefrontal cortex of maternally-separated rat pups. *The journal of physiological sciences : JPS* 2012; **62**(4)**:** 351-357.

186. Hoyo-Becerra C, Huebener A, Trippler M, Lutterbeck M, Liu ZJ, Truebner K *et al.* Concomitant interferon alpha stimulation and TLR3 activation induces neuronal expression of depression-related genes that are elevated in the brain of suicidal persons. *PLoS ONE* 2013; **8**(12)**:** e83149.

187. Gasparyan A, Navarrete F, Manzanares J. Cannabidiol and Sertraline Regulate Behavioral and Brain Gene Expression Alterations in an Animal Model of PTSD. *Front Pharmacol* 2021; **12:** 694510.

188. Jawinski P, Kirsten H, Sander C, Spada J, Ulke C, Huang J *et al.* Human brain arousal in the resting state: a genome-wide association study. *Mol Psychiatry* 2019; **24**(11)**:** 1599-1609.

189. Ward J, Tunbridge EM, Sandor C, Lyall LM, Ferguson A, Strawbridge RJ *et al.* The genomic basis of mood instability: identification of 46 loci in 363,705 UK Biobank participants, genetic correlation with psychiatric disorders, and association with gene expression and function. *Mol Psychiatry* 2020; **25**(11)**:** 3091-3099.

190. Jia X, Goes FS, Locke AE, Palmer D, Wang W, Cohen-Woods S *et al.* Investigating rare pathogenic/likely pathogenic exonic variation in bipolar disorder. *Mol Psychiatry* 2021.

191. Cabrera-Mendoza B, Fresno C, Monroy-Jaramillo N, Fries GR, Walss-Bass C, Glahn DC *et al.* Sex differences in brain gene expression among suicide completers. *J Affect Disord* 2020; **267:** 67-77.

192. Mehta D, Klengel T, Conneely KN, Smith AK, Altmann A, Pace TW *et al.* Childhood maltreatment is associated with distinct genomic and epigenetic profiles in posttraumatic stress disorder. *Proc Natl Acad Sci U S A* 2013; **110**(20)**:** 8302-8307.

193. Cattaneo A, Cattane N, Malpighi C, Czamara D, Suarez A, Mariani N *et al.* FoxO1, A2M, and TGF-beta1: three novel genes predicting depression in gene X environment interactions are identified using cross-species and cross-tissues transcriptomic and miRNomic analyses. *Mol Psychiatry* 2018; **23**(11)**:** 2192-2208.

194. Kupfer DM, White VL, Strayer DL, Crouch DJ, Burian D. Microarray characterization of gene expression changes in blood during acute ethanol exposure. *BMC Med Genomics* 2013; **6:** 26.

195. Gazestani VH, Pramparo T, Nalabolu S, Kellman BP, Murray S, Lopez L *et al.* A perturbed gene network containing PI3K-AKT, RAS-ERK and WNT-beta-catenin pathways in leukocytes is linked to ASD genetics and symptom severity. *Nat Neurosci* 2019; **22**(10)**:** 1624-1634.

196. Erickson EK, Blednov YA, Harris RA, Mayfield RD. Glial gene networks associated with alcohol dependence. *Sci Rep* 2019; **9**(1)**:** 10949.

197. Kataoka M, Matoba N, Sawada T, Kazuno AA, Ishiwata M, Fujii K *et al.* Exome sequencing for bipolar disorder points to roles of de novo loss-of-function and protein-altering mutations. *Mol Psychiatry* 2016; **21**(7)**:** 885-893.

198. Sears C, Wilson J, Fitches A. Investigating the role of BDNF and CCK system genes in suicidality in a familial bipolar cohort. *J Affect Disord* 2013; **151**(2)**:** 611-617.

199. Sherrin T, Heng KY, Zhu YZ, Tang YM, Lau G, Tan CH. Cholecystokinin-B receptor gene expression in cerebellum, pre-frontal cortex and cingulate gyrus and its association with suicide. *Neurosci Lett* 2004; **357**(2)**:** 107-110.

200. Aston C, Jiang L, Sokolov BP. Transcriptional profiling reveals evidence for signaling and oligodendroglial abnormalities in the temporal cortex from patients with major depressive disorder. *Mol Psychiatry* 2005; **10**(3)**:** 309-322.

201. Bowen EFW, Burgess JL, Granger R, Kleinman JE, Rhodes CH. DLPFC transcriptome defines two molecular subtypes of schizophrenia. *Transl Psychiatry* 2019; **9**(1)**:** 147.

202. Le-Niculescu H, McFarland MJ, Ogden CA, Balaraman Y, Patel S, Tan J *et al.* Phenomic, convergent functional genomic, and biomarker studies in a stress-reactive genetic animal model of bipolar disorder and co-morbid alcoholism. *Am J Med Genet B Neuropsychiatr Genet* 2008; **147B**(2)**:** 134-166.

203. Musaelyan K, Yildizoglu S, Bozeman J, Du Preez A, Egeland M, Zunszain PA *et al.* Chronic stress induces significant gene expression changes in the prefrontal cortex alongside alterations in adult hippocampal neurogenesis. *Brain Commun* 2020; **2**(2)**:** fcaa153.

204. Roussos P, Guennewig B, Kaczorowski DC, Barry G, Brennand KJ. Activity-Dependent Changes in Gene Expression in Schizophrenia Human-Induced Pluripotent Stem Cell Neurons. *JAMA psychiatry* 2016; **73**(11)**:** 1180-1188.

205. Patel H, Hodges AK, Curtis C, Lee SH, Troakes C, Dobson RJB *et al.* Transcriptomic analysis of probable asymptomatic and symptomatic alzheimer brains. *Brain Behav Immun* 2019; **80:** 644-656.

206. Ogden CA, Rich ME, Schork NJ, Paulus MP, Geyer MA, Lohr JB *et al.* Candidate genes, pathways and mechanisms for bipolar (manic-depressive) and related disorders: an expanded convergent functional genomics approach. *Mol Psychiatry* 2004; **9**(11)**:** 1007-1029.

207. Erickson EK, Farris SP, Blednov YA, Mayfield RD, Harris RA. Astrocyte-specific transcriptome responses to chronic ethanol consumption. *Pharmacogenomics J* 2018; **18**(4)**:** 578-589.

208. Kang YK, Min B, Eom J, Park JS. Different phases of aging in mouse old skeletal muscle. *Aging (Albany NY)* 2022; **14**(1)**:** 143-160.

209. Georgieva L, Dimitrova A, Ivanov D, Nikolov I, Williams NM, Grozeva D *et al.* Support for neuregulin 1 as a susceptibility gene for bipolar disorder and schizophrenia. *Biol Psychiatry* 2008; **64**(5)**:** 419-427.

210. Uhl GR, Drgon T, Liu QR, Johnson C, Walther D, Komiyama T *et al.* Genome-wide association for methamphetamine dependence: convergent results from 2 samples. *Arch Gen Psychiatry* 2008; **65**(3)**:** 345-355.

211. Bousman CA, Yung AR, Pantelis C, Ellis JA, Chavez RA, Nelson B *et al.* Effects of NRG1 and DAOA genetic variation on transition to psychosis in individuals at ultra-high risk for psychosis. *Transl Psychiatry* 2013; **3:** e251.

212. Mostaid MS, Lee TT, Chana G, Sundram S, Shannon Weickert C, Pantelis C *et al.* Elevated peripheral expression of neuregulin-1 (NRG1) mRNA isoforms in clozapine-treated schizophrenia patients. *Transl Psychiatry* 2017; **7**(12)**:** 1280.

213. Wellcome Trust Case Control C. Genome-wide association study of 14,000 cases of seven common diseases and 3,000 shared controls. *Nature* 2007; **447**(7145)**:** 661-678.

214. Hall LS, Adams MJ, Arnau-Soler A, Clarke TK, Howard DM, Zeng Y *et al.* Genome-wide meta-analyses of stratified depression in Generation Scotland and UK Biobank. *Transl Psychiatry* 2018; **8**(1)**:** 9.

215. Howard DM, Adams MJ, Clarke TK, Hafferty JD, Gibson J, Shirali M *et al.* Genome-wide meta-analysis of depression identifies 102 independent variants and highlights the importance of the prefrontal brain regions. *Nat Neurosci* 2019; **22**(3)**:** 343-352.

216. Levchenko A, Vyalova NM, Nurgaliev T, Pozhidaev IV, Simutkin GG, Bokhan NA *et al.* NRG1, PIP4K2A, and HTR2C as Potential Candidate Biomarker Genes for Several Clinical Subphenotypes of Depression and Bipolar Disorder. *Frontiers in genetics* 2020; **11:** 936.

217. Peyrot WJ, Price AL. Identifying loci with different allele frequencies among cases of eight psychiatric disorders using CC-GWAS. *Nat Genet* 2021; **53**(4)**:** 445-454.

218. Marballi K, Cruz D, Thompson P, Walss-Bass C. Differential neuregulin 1 cleavage in the prefrontal cortex and hippocampus in schizophrenia and bipolar disorder: preliminary findings. *PLoS ONE* 2012; **7**(5)**:** e36431.

219. Tkachev D, Mimmack ML, Ryan MM, Wayland M, Freeman T, Jones PB *et al.* Oligodendrocyte dysfunction in schizophrenia and bipolar disorder. *Lancet* 2003; **362**(9386)**:** 798-805.

220. Sheng G, Demers M, Subburaju S, Benes FM. Differences in the circuitry-based association of copy numbers and gene expression between the hippocampi of patients with schizophrenia and the hippocampi of patients with bipolar disorder. *Arch Gen Psychiatry* 2012; **69**(6)**:** 550-561.

221. Hahn CG, Wang HY, Cho DS, Talbot K, Gur RE, Berrettini WH *et al.* Altered neuregulin 1-erbB4 signaling contributes to NMDA receptor hypofunction in schizophrenia. *Nat Med* 2006; **12**(7)**:** 824-828.

222. Chong VZ, Thompson M, Beltaifa S, Webster MJ, Law AJ, Weickert CS. Elevated neuregulin-1 and ErbB4 protein in the prefrontal cortex of schizophrenic patients. *Schizophr Res* 2008; **100**(1-3)**:** 270-280.

223. Law AJ, Wang Y, Sei Y, O'Donnell P, Piantadosi P, Papaleo F *et al.* Neuregulin 1-ErbB4-PI3K signaling in schizophrenia and phosphoinositide 3-kinase-p110delta inhibition as a potential therapeutic strategy. *Proc Natl Acad Sci U S A* 2012; **109**(30)**:** 12165-12170.

224. Jaffe AE, Gao Y, Deep-Soboslay A, Tao R, Hyde TM, Weinberger DR *et al.* Mapping DNA methylation across development, genotype and schizophrenia in the human frontal cortex. *Nat Neurosci* 2016; **19**(1)**:** 40-47.

225. Petryshen TL, Middleton FA, Kirby A, Aldinger KA, Purcell S, Tahl AR *et al.* Support for involvement of neuregulin 1 in schizophrenia pathophysiology. *Mol Psychiatry* 2005; **10**(4)**:** 366-374, 328.

226. Vawter MP, Philibert R, Rollins B, Ruppel PL, Osborn TW. Exon Array Biomarkers for the Differential Diagnosis of Schizophrenia and Bipolar Disorder. *Mol Neuropsychiatry* 2018; **3**(4)**:** 197-213.

227. Fernandez-Castillo N, Cabana-Dominguez J, Soriano J, Sanchez-Mora C, Roncero C, Grau-Lopez L *et al.* Transcriptomic and genetic studies identify NFAT5 as a candidate gene for cocaine dependence. *Transl Psychiatry* 2015; **5:** e667.

228. Brennand KJ, Simone A, Jou J, Gelboin-Burkhart C, Tran N, Sangar S *et al.* Modelling schizophrenia using human induced pluripotent stem cells. *Nature* 2011; **473**(7346)**:** 221-225.

229. Middleton FA, Pato CN, Gentile KL, McGann L, Brown AM, Trauzzi M *et al.* Gene expression analysis of peripheral blood leukocytes from discordant sib-pairs with schizophrenia and bipolar disorder reveals points of convergence between genetic and functional genomic approaches. *Am J Med Genet B Neuropsychiatr Genet* 2005; **136B**(1)**:** 12-25.

230. Begemann M, Sargin D, Rossner MJ, Bartels C, Theis F, Wichert SP *et al.* Episode-specific differential gene expression of peripheral blood mononuclear cells in rapid cycling supports novel treatment approaches. *Mol Med* 2008; **14**(9-10)**:** 546-552.

231. Belzeaux R, Bergon A, Jeanjean V, Loriod B, Formisano-Treziny C, Verrier L *et al.* Responder and nonresponder patients exhibit different peripheral transcriptional signatures during major depressive episode. *Transl Psychiatry* 2012; **2:** e185.

232. Miller GE, Chen E, Sze J, Marin T, Arevalo JM, Doll R *et al.* A functional genomic fingerprint of chronic stress in humans: blunted glucocorticoid and increased NF-kappaB signaling. *Biol Psychiatry* 2008; **64**(4)**:** 266-272.

233. de Kluiver H, Jansen R, Milaneschi Y, Penninx B. Involvement of inflammatory gene expression pathways in depressed patients with hyperphagia. *Transl Psychiatry* 2019; **9**(1)**:** 193.

234. Wittenberg GM, Greene J, Vertes PE, Drevets WC, Bullmore ET. Major Depressive Disorder Is Associated With Differential Expression of Innate Immune and Neutrophil-Related Gene Networks in Peripheral Blood: A Quantitative Review of Whole-Genome Transcriptional Data From Case-Control Studies. *Biol Psychiatry* 2020; **88**(8)**:** 625-637.

235. Lin CH, Huang MW, Lin CH, Huang CH, Lane HY. Altered mRNA expressions for N-methyl-D-aspartate receptor-related genes in WBC of patients with major depressive disorder. *J Affect Disord* 2019; **245:** 1119-1125.

236. Chang KA, Shin KY, Nam E, Lee YB, Moon C, Suh YH *et al.* Plasma soluble neuregulin-1 as a diagnostic biomarker for Alzheimer's disease. *Neurochem Int* 2016; **97:** 1-7.

237. Segman RH, Shefi N, Goltser-Dubner T, Friedman N, Kaminski N, Shalev AY. Peripheral blood mononuclear cell gene expression profiles identify emergent post-traumatic stress disorder among trauma survivors. *Mol Psychiatry* 2005; **10**(5)**:** 500-513, 425.

238. He S, Deng Z, Li Z, Gao W, Zeng D, Shi Y *et al.* Signatures of 4 autophagy-related genes as diagnostic markers of MDD and their correlation with immune infiltration. *J Affect Disord* 2021; **295:** 11-20.

239. Long LE, Chesworth R, Huang XF, Wong A, Spiro A, McGregor IS *et al.* Distinct neurobehavioural effects of cannabidiol in transmembrane domain neuregulin 1 mutant mice. *PLoS One* 2012; **7**(4)**:** e34129.

240. Stefansson H, Sigurdsson E, Steinthorsdottir V, Bjornsdottir S, Sigmundsson T, Ghosh S *et al.* Neuregulin 1 and susceptibility to schizophrenia. *Am J Hum Genet* 2002; **71**(4)**:** 877-892.

241. O'Tuathaigh CM, Babovic D, O'Sullivan GJ, Clifford JJ, Tighe O, Croke DT *et al.* Phenotypic characterization of spatial cognition and social behavior in mice with 'knockout' of the schizophrenia risk gene neuregulin 1. *Neuroscience* 2007; **147**(1)**:** 18-27.

242. Chen YJ, Johnson MA, Lieberman MD, Goodchild RE, Schobel S, Lewandowski N *et al.* Type III neuregulin-1 is required for normal sensorimotor gating, memory-related behaviors, and corticostriatal circuit components. *J Neurosci* 2008; **28**(27)**:** 6872-6883.

243. Papaleo F, Yang F, Paterson C, Palumbo S, Carr GV, Wang Y *et al.* Behavioral, Neurophysiological, and Synaptic Impairment in a Transgenic Neuregulin1 (NRG1-IV) Murine Schizophrenia Model. *J Neurosci* 2016; **36**(17)**:** 4859-4875.

244. Muhie S, Gautam A, Meyerhoff J, Chakraborty N, Hammamieh R, Jett M. Brain transcriptome profiles in mouse model simulating features of post-traumatic stress disorder. *Mol Brain* 2015; **8:** 14.

245. Zhang L, Li H, Hu X, Benedek DM, Fullerton CS, Forsten RD *et al.* Mitochondria-focused gene expression profile reveals common pathways and CPT1B dysregulation in both rodent stress model and human subjects with PTSD. *Transl Psychiatry* 2015; **5:** e580.

246. Olaya JC, Heusner CL, Matsumoto M, Shannon Weickert C, Karl T. Schizophrenia-relevant behaviours of female mice overexpressing neuregulin 1 type III. *Behav Brain Res* 2018; **353:** 227-235.

247. Elfving B, Muller HK, Oliveras I, Osterbog TB, Rio-Alamos C, Sanchez-Gonzalez A *et al.* Differential expression of synaptic markers regulated during neurodevelopment in a rat model of schizophrenia-like behavior. *Prog Neuropsychopharmacol Biol Psychiatry* 2019; **95:** 109669.

248. Voutetakis K, Chatziioannou A, Gonos ES, Trougakos IP. Comparative Meta-Analysis of Transcriptomics Data during Cellular Senescence and In Vivo Tissue Ageing. *Oxid Med Cell Longev* 2015; **2015:** 732914.

249. Szatkiewicz JP, O'Dushlaine C, Chen G, Chambert K, Moran JL, Neale BM *et al.* Copy number variation in schizophrenia in Sweden. *Mol Psychiatry* 2014; **19**(7)**:** 762-773.

250. Scarr E, Udawela M, Dean B. Changed frontal pole gene expression suggest altered interplay between neurotransmitter, developmental, and inflammatory pathways in schizophrenia. *NPJ Schizophr* 2018; **4**(1)**:** 4.

251. Castillo E, Leon J, Mazzei G, Abolhassani N, Haruyama N, Saito T *et al.* Comparative profiling of cortical gene expression in Alzheimer's disease patients and mouse models demonstrates a link between amyloidosis and neuroinflammation. *Sci Rep* 2017; **7**(1)**:** 17762.

252. Sood S, Gallagher IJ, Lunnon K, Rullman E, Keohane A, Crossland H *et al.* A novel multi-tissue RNA diagnostic of healthy ageing relates to cognitive health status. *Genome Biol* 2015; **16:** 185.

253. Ciobanu LG, Sachdev PS, Trollor JN, Reppermund S, Thalamuthu A, Mather KA *et al.* Downregulated transferrin receptor in the blood predicts recurrent MDD in the elderly cohort: A fuzzy forests approach. *J Affect Disord* 2020; **267:** 42-48.

254. Haselton FR, Heimark RL. Role of cadherins 5 and 13 in the aortic endothelial barrier. *J Cell Physiol* 1997; **171**(3)**:** 243-251.

255. Le-Niculescu H, Case NJ, Hulvershorn L, Patel SD, Bowker D, Gupta J *et al.* Convergent functional genomic studies of omega-3 fatty acids in stress reactivity, bipolar disorder and alcoholism. *Transl Psychiatry* 2011; **1:** e4.

256. Warburton A, Savage AL, Myers P, Peeney D, Bubb VJ, Quinn JP. Molecular signatures of mood stabilisers highlight the role of the transcription factor REST/NRSF. *J Affect Disord* 2015; **172:** 63-73.

257. Schulpen SH, Pennings JL, Piersma AH. Gene Expression Regulation and Pathway Analysis After Valproic Acid and Carbamazepine Exposure in a Human Embryonic Stem Cell-Based Neurodevelopmental Toxicity Assay. *Toxicol Sci* 2015; **146**(2)**:** 311-320.

258. Rodrigues P, Cunha V, Oliva-Teles L, Ferreira M, Guimaraes L. Effects of norfluoxetine and venlafaxine in zebrafish larvae: Molecular data. *Data Brief* 2020; **33:** 106515.

259. Breitfeld J, Scholl C, Steffens M, Brandenburg K, Probst-Schendzielorz K, Efimkina O *et al.* Proliferation rates and gene expression profiles in human lymphoblastoid cell lines from patients with depression characterized in response to antidepressant drug therapy. *Transl Psychiatry* 2016; **6**(11)**:** e950.

260. Panizzutti B, Bortolasci CC, Spolding B, Kidnapillai S, Connor T, Richardson MF *et al.* Transcriptional Modulation of the Hippo Signaling Pathway by Drugs Used to Treat Bipolar Disorder and Schizophrenia. *International journal of molecular sciences* 2021; **22**(13).

261. Iaccarino HF, Singer AC, Martorell AJ, Rudenko A, Gao F, Gillingham TZ *et al.* Gamma frequency entrainment attenuates amyloid load and modifies microglia. *Nature* 2016; **540**(7632)**:** 230-235.

262. Palmos AB, Duarte RRR, Smeeth DM, Hedges EC, Nixon DF, Thuret S *et al.* Lithium treatment and human hippocampal neurogenesis. *Transl Psychiatry* 2021; **11**(1)**:** 555.

263. MacDonald ML, Eaton ME, Dudman JT, Konradi C. Antipsychotic drugs elevate mRNA levels of presynaptic proteins in the frontal cortex of the rat. *Biol Psychiatry* 2005; **57**(9)**:** 1041-1051.

264. Cabrera-Mendoza B, Martinez-Magana JJ, Monroy-Jaramillo N, Genis-Mendoza AD, Fresno C, Fries GR *et al.* Candidate pharmacological treatments for substance use disorder and suicide identified by gene co-expression network-based drug repositioning. *Am J Med Genet B Neuropsychiatr Genet* 2021; **186**(3)**:** 193-206.

265. Hammamieh R, Chakraborty N, Gautam A, Miller SA, Muhie S, Meyerhoff J *et al.* Transcriptomic analysis of the effects of a fish oil enriched diet on murine brains. *PLoS ONE* 2014; **9**(3)**:** e90425.

266. Rangaraju S, Levey DF, Nho K, Jain N, Andrews KD, Le-Niculescu H *et al.* Mood, stress and longevity: convergence on ANK3. *Mol Psychiatry* 2016; **21**(8)**:** 1037-1049.

267. Benton CS, Miller BH, Skwerer S, Suzuki O, Schultz LE, Cameron MD *et al.* Evaluating genetic markers and neurobiochemical analytes for fluoxetine response using a panel of mouse inbred strains. *Psychopharmacology (Berl)* 2012; **221**(2)**:** 297-315.

268. Lopez JP, Lim R, Cruceanu C, Crapper L, Fasano C, Labonte B *et al.* miR-1202 is a primate-specific and brain-enriched microRNA involved in major depression and antidepressant treatment. *Nat Med* 2014; **20**(7)**:** 764-768.

269. Nohr AK, Lindow M, Forsingdal A, Demharter S, Nielsen T, Buller R *et al.* A large-scale genome-wide gene expression analysis in peripheral blood identifies very few differentially expressed genes related to antidepressant treatment and response in patients with major depressive disorder. *Neuropsychopharmacology* 2021; **46**(7)**:** 1324-1332.

270. Gasparyan A, Navarrete F, Manzanares J. The administration of sertraline plus naltrexone reduces ethanol consumption and motivation in a long-lasting animal model of post-traumatic stress disorder. *Neuropharmacology* 2021; **189:** 108552.

271. Martin RE, Green MT, Kinkade JA, Schmidt RR, Willemse TE, Schenk AK *et al.* Maternal Oxycodone Treatment Results in Neurobehavioral Disruptions in Mice Offspring. *eNeuro* 2021; **8**(4).

272. Kikuchi M, Nakazawa T, Kinoshita M, Yamamori H, Yasuda Y, Fujimoto M *et al.* Methylation Analysis in Monozygotic Twins With Treatment-Resistant Schizophrenia and Discordant Responses to Clozapine. *Front Psychiatry* 2021; **12:** 734606.

273. Wang N, Zhang GF, Liu XY, Sun HL, Wang XM, Qiu LL *et al.* Downregulation of neuregulin 1-ErbB4 signaling in parvalbumin interneurons in the rat brain may contribute to the antidepressant properties of ketamine. *J Mol Neurosci* 2014; **54**(2)**:** 211-218.

274. Gow M, Mirembe D, Longwe Z, Pickard BS. A gene trap mutagenesis screen for genes underlying cellular response to the mood stabilizer lithium. *J Cell Mol Med* 2013; **17**(5)**:** 657-663.

275. Sainz J, Prieto C, Crespo-Facorro B. Sex differences in gene expression related to antipsychotic induced weight gain. *PLoS ONE* 2019; **14**(4)**:** e0215477.

276. Hagihara H, Ohira K, Miyakawa T. Transcriptomic evidence for immaturity induced by antidepressant fluoxetine in the hippocampus and prefrontal cortex. *Neuropsychopharmacol Rep* 2019; **39**(2)**:** 78-89.

277. Hill EJ, Nagel DA, O'Neil JD, Torr E, Woehrling EK, Devitt A *et al.* Effects of lithium and valproic acid on gene expression and phenotypic markers in an NT2 neurosphere model of neural development. *PLoS ONE* 2013; **8**(3)**:** e58822.

278. Viana J, Wildman N, Hannon E, Farbos A, Neill PO, Moore K *et al.* Clozapine-induced transcriptional changes in the zebrafish brain. *NPJ Schizophr* 2020; **6**(1)**:** 3.

279. Wang XM, Wu TX, Hamza M, Ramsay ES, Wahl SM, Dionne RA. Rofecoxib modulates multiple gene expression pathways in a clinical model of acute inflammatory pain. *Pain* 2007; **128**(1-2)**:** 136-147.

280. Guo J, Cheng J, Zheng N, Zhang X, Dai X, Zhang L *et al.* Copper Promotes Tumorigenesis by Activating the PDK1-AKT Oncogenic Pathway in a Copper Transporter 1 Dependent Manner. *Adv Sci (Weinh)* 2021; **8**(18)**:** e2004303.

281. Nass J, Kampf CJ, Efferth T. Increased Stress Resistance and Lifespan in Chaenorhabditis elegans Wildtype and Knockout Mutants-Implications for Depression Treatment by Medicinal Herbs. *Molecules* 2021; **26**(7).
