# Supplementary material for: Towards precision medicine for anxiety disorders: objective assessment, risk prediction, pharmacogenomics, and repurposed drugs
Source: Mol Psychiatry. 2023 Mar 7;28(7):2894–912. doi: 10.1038/s41380-023-01998-0 (PMC10615756; doi:10.1038/s41380-023-01998-0)
Supplement: Supplementary file 2 — Supplementary Information- Materials and Methods [file 41380_2023_1998_MOESM2_ESM.docx]

**Materials and Methods**

**Cohorts**

We used three independent cohorts: discovery (major psychiatric disorders with changes in *state* anxiety), validation (major psychiatric disorders with clinically severe anxiety), and testing (an independent major psychiatric disorders cohort for predicting *state* anxiety, and for predicting *trait* anxiety (future hospitalization with anxiety as the primary reason) (Figure 1A).

Similar to our previous studies[^2^](#_ENREF_2)^,^[^3^](#_ENREF_3)^,^[^4^](#_ENREF_4), the psychiatric subjects are part of a larger longitudinal cohort of adults that we are continuously collecting. Subjects were recruited from the patient population at the Indianapolis VA Medical Center. All subjects understood and signed informed consent forms detailing the research goals, procedure, caveats, and safeguards, per IRB approved protocol. Subjects completed diagnostic assessments and extensive structured neuropsychological testing at each testing visit, 3–6 months apart or whenever a new psychiatric hospitalization occurred. At each testing visit, they received a series of rating scales, including a self-report visual analog scale (1-100) for quantitatively assessing *state* anxiety at that particular moment in time (Simplified Anxiety Scale- SAS-4). This 4- item scale looks at anxiety overall, as well as fear, anger, and uncertainty. Each of the items are a VAS of 0 to 100, related to that moment in time. As such, it generates temporal, quantitative, and targeted data.

At each testing visit we collected whole blood (10 ml) in two RNA-stabilizing PAXgene tubes, labeled with an anonymized ID number, and stored at -80 degrees C in a locked freezer until the time of future processing. Whole-blood RNA was extracted for microarray gene expression studies from the PAXgene tubes, as detailed below.

For this study, our within-subject discovery cohort, from which the biomarker data were derived, consisted of 58 subjects (41 males, 17 females) with multiple testing visits, who each had at least one diametric change in anxiety *state* from low anxiety *state* (SAS-4 score of ≤ 40/100) to a high anxiety *state* (SAS-4 score of ≥ 60/100), or vice versa, from one testing visit to another (Figures 1a,b and Figure S1). There were 2 subjects with 5 visits each, 3 subjects with 4 visits each, 21 subjects with 3 visits each, and 32 subjects with 2 visits each resulting in a total of 149 blood samples for subsequent gene expression microarray studies (Figure 1a, b, Table 1 and S1).

Our independent validation cohort, in which the top biomarker findings were validated for being even more changed in expression, consisted of 40 subjects (32 male and 8 female) with clinically severe anxiety (SAS-4 scores ≥60, and concordant high anxiety STAI State scores ≥ 55 (Table 1 and S1).

For testing the biomarkers, we used an independent test cohort.

For state predictions, we predicted high anxiety state (SAS-4 ≥ 60) (161 male and 36 female subjects) and clinically severe anxiety (STAI ≥ 55) (159 male and 36 female subjects) (Figure 1 and Table 1).

For *trait* predictions of future hospitalizations with anxiety as a contributory reason (Figure 1 and Table 1), we used a subset of the independent test cohort for which we had longitudinal follow-up with electronic medical records. The subjects’ subsequent number of hospitalizations with anxiety was tabulated from electronic medical records.

*Medications.* The subjects in the discovery cohort were all diagnosed with various psychiatric disorders (Table 1) and had various medical co-morbidities. Their medications were listed in their electronic medical records, and documented by us at the time of each testing visit. Medications can have a strong influence on gene expression. However, there was no consistent pattern of any particular type of medication, as our subjects were on a wide variety of different medications, psychiatric and non-psychiatric. Furthermore, the independent validation and testing cohort’s gene expression data was Z-scored by gender and by diagnosis before being combined, to normalize for any such effects. Some subjects may be non-compliant with their treatment and may thus have changes in medications or drugs of abuse not reflected in their medical records. That being said, our goal is to find biomarkers that track anxiety, regardless if the reason for it is endogenous biology or it is driven by medications or drugs. In fact, one would expect some of these biomarkers to be targets of medications, as we show in this paper. Moreover, the prioritization step that occurs after discovery is based on a field-wide convergence with literature that includes genetic data and animal model data, that are unrelated to medication effects. Overall, the discovery, validation, and replication by testing in independent cohorts of the biomarkers, with our design, occurs despite the subjects having different genders, diagnoses, being on various different medications, and other lifestyle variables*.*

**Blood gene expression experiments**

*RNA extraction*. Whole blood (2.5 ml) was collected into each PaxGene tube by routine venipuncture. PaxGene tubes contain proprietary reagents for the stabilization of RNA. Total RNA was extracted and processed as previously described [^2^](#_ENREF_2)^,^[^3^](#_ENREF_3)^,^[^4^](#_ENREF_4).

*Microarrays.* Microarray work was carried out using previously described methodology[^2^](#_ENREF_2)^,^[^3^](#_ENREF_3)^,^[^4^](#_ENREF_4)^,^ [^5^](#_ENREF_5).

Of note, all genomic data was normalized (RMA for technical variability, then z-scoring for biological variability), by gender and psychiatric diagnosis, before being combined and analyzed.

See Supplementary Information for rest of Materials and Methods.

**Biomarkers**

*Step 1: Discovery*

We have used the subject’s score from a visual-analog scale (SAS-4) scale, assessed at the time of blood collection (Figure 1). We analyzed gene expression differences between visits with low anxiety (defined as a score of 0-40) and visits with high anxiety (defined as a score of 60 -100), using a powerful within-subject design, then an across-subjects summation (Figure 1).

We analyzed the data in two ways: an Absent-Present (AP) approach, and a differential expression (DE) approach, as in previous work by us on suicide biomarkers[^2-4^](#_ENREF_2). The AP approach may capture turning on and off of genes, and the DE approach may capture gradual changes in expression. Analyses were performed as previously described[^3-5^](#_ENREF_3). In brief, we imported all Affymetrix microarray data as CEL. files into Partek Genomic Suites 6.6 software package (Partek Incorporated, St Louis, MO, USA). Using only the perfect match values, we ran a robust multi-array analysis (RMA) by gender and diagnosis, background corrected with quantile normalization and a median polish probeset summarization of all chips, to obtain the normalized expression levels of all probesets for each chip. Then, to establish a list of differentially expressed probesets we conducted a within-subject analysis, using a fold change in expression of at least 1.2 between consecutive high- and low anxiety visits within each subject. Probesets that have a 1.2-fold change are then assigned either a 1 (increased in high anxiety) or a -1 (decreased in high anxiety) in each comparison. Fold changes between 1.1 and 1.2 are given 0.5, and fold changes less than 1.1 are given 0. These values were then summed for each probeset across all the comparisons and subjects, yielding a range of raw scores. The probesets above the 33.3% of raw scores were carried forward in analyses (Figure 1), and received an internal score of 2 points; those above 50% 4 points, and those above 80% 6 points[^3^](#_ENREF_3) [^4^](#_ENREF_4) [^5^](#_ENREF_5). We have developed in our labs R scripts to automate and conduct all these large dataset analyses in bulk, checked against human manual scoring[^5^](#_ENREF_5).

Gene Symbol for the probesets were identified using NetAffyx (Affymetrix) for Affymetrix HG-U133 Plus 2.0 GeneChips, followed by GeneCards to confirm the primary gene symbol. In addition, for those probesets that were not assigned a gene symbol by NetAffyx, we used GeneAnnot (<https://genecards.weizmann.ac.il/geneannot/index.shtml>), or if need be UCSC (<https://genome.ucsc.edu>), to obtain gene symbol for these uncharacterized probesets, followed by GeneCard. Genes were then scored using our manually curated CFG databases as described below (Figure 1C).

*Step 2: Prioritization using Convergent Functional Genomics (CFG)*

Databases. We have established in our laboratory (Laboratory of Neurophenomics, [www.neurophenomics.info](http://www.neurophenomics.info)) manually curated databases of the human gene expression/protein expression studies (postmortem brain, peripheral tissue/fluids: CSF, blood and cell cultures), human genetic studies (association, copy number variations and linkage), and animal model gene expression and genetic studies, published to date on psychiatric disorders. Only findings deemed significant in the primary publication, by the study authors, using their particular experimental design and thresholds, are included in our databases. Our databases include only primary literature data and do not include review papers or other secondary data integration analyses to avoid redundancy and circularity. We also favored unbiased discovery studies over candidate genes hypothesis-driven studies. These large and constantly updated databases have been used in our CFG cross validation and prioritization platform (Figure 1E). For this study, data from 354 papers on anxiety were present in the databases at the time of the CFG analyses (July 2019) (human genetic studies-93, human brain studies-10, human peripheral tissue/fluids- 96, non-human genetic studies-17, non-human brain studies-123, non-human peripheral tissue/fluids- 17). Analyses were performed as previously described[^3^](#_ENREF_3)^,^ [^4^](#_ENREF_4). We have developed in our lab a computerized CFG Wizard to automate and score in bulk large lists of genes by integrating evidence from these large databases, checked against manual scoring [^5^](#_ENREF_5). Analyses were performed as previously described[^3^](#_ENREF_3)^,^ [^4^](#_ENREF_4).

*Step 3: Validation analyses*

We examined which of the top candidate genes (score of 6 or above after the first two steps), were changed in expression even more in an independent validation cohort (n=40) of clinically severe anxiety as measured by a STAI State ≥55, as well as a SAS-4 ≥ 60. A total score of 6 or above after the first two steps permits the inclusion of potentially novel genes with maximal internal score of 6 from Discovery but no external evidence CFG score from Prioritization.

Subjects with low anxiety as well as subjects with high anxiety from the discovery cohort who did not have clinically severe anxiety were used, along with the independent validation cohort. We looked for stepwise change from the discovery cohort low anxiety group to the discovery cohort high anxiety group to the validation cohort clinically severe group.

The AP derived and DE derived lists of genes were combined, and the gene expression data corresponding to them was used for the validation analysis. The 3 groups (low anxiety, high anxiety, clinical severe anxiety) were assembled out of Affymetrix .cel data that was RMA normalized by gender and diagnosis. We transferred the log transformed expression data to an Excel sheet, and non-log transformed the data by taking 2 to the power of the transformed expression value. We then Z-scored the values by gender and diagnosis. We then imported the Excel sheets with the Z-scored by gender and diagnosis expression data into Partek, and statistical analyses were performed using a one-way ANOVA for the stepwise changed probesets, and also did a stringent Bonferroni correction for all the probesets tested in ANOVA (Figure 1E).

**Top Candidate Biomarkers (after the first 3 Steps)**

Adding the scores from the first three steps into an overal convergent functional evidence (CFE) score (Figure 1E), we ended up with a list of 95 top candidate biomarkers (95 probesets in n=82 genes), that had a CFE score of 8 and above (out of a maximum possible of 24 ; ≥33%) (see also Supplementary Information). These 95 top candidate biomarkers were carried forward into additional analyses for biological understanding, and for testing for clinical utility (Step 4).

**Biological Understanding**

*Pathway Analyses*

IPA (Ingenuity Pathway Analysis, version 24390178, Qiagen), David Functional Annotation Bioinformatics Microarray Analysis (National Institute of Allergy and Infectious Diseases) version 6.7 (August 2016), and Kyoto Encyclopedia of Genes and Genomes (KEGG) (through DAVID) were used to analyze biological roles, including top canonical pathways and diseases (Table 3).

*Networks*

For network analyses we performed STRING Interaction network (https://string-db.org) by inputting the genes into the search window, and performed Multiple Proteins Homo sapiens analysis (Figure 3).

*CFG beyond Anxiety: evidence for involvement in other psychiatric and related disorders.*

We also used a CFG approach to examine evidence from other psychiatric and related disorders, as exemplified for the list of top biomarkers after Step 4 testing (Table S3). This was not used to prioritize genes, but rather to understand the molecular basis of clinical co-morbidities.

**Testing for Clinical Utility in Independent Cohorts**

We tested in independent cohorts of psychiatric patients the ability of each of the top candidate biomarkers (n=95) to assess current severity of anxiety (*state* -measured by SAS-4 or STAI), and predict future risk of anxiety (*trait* -future hospitalizations with anxiety). We conducted our analyses across all patients, as well as personalized by gender and diagnosis.

The test cohort for predicting high anxiety (*state*), and the test cohort for predicting future hospitalizations with anxiety (*trait*), were assembled out of data that was RMA normalized by gender and diagnosis. The cohort was completely independent from the discovery and validation cohorts, there was no subject overlap with them. Individual markers used for predictions were Z scored by gender and diagnosis, to be able to combine different biomarkers into panels and to avoid potential artefacts due to different ranges of expression in different gender and diagnoses. For panels, biomarkers were combined by simple summation of the increased risk biomarkers minus the decreased risk biomarkers. Predictions were performed using R-studio. For cross-sectional analyses, we used biomarker expression levels, z-scored by gender and diagnosis. For longitudinal analyses, we combined four measures: biomarker expression levels, slope (defined as ratio of levels at current testing visit vs. previous visit, divided by time between visits), maximum levels (at any of the current or past visits), and maximum slope (between any adjacent current or past visits). For decreased biomarkers, we used the minimum rather than the maximum for level calculations. All four measures were Z-scored, then combined in an additive fashion into a single measure.  The longitudinal analysis was carried out in a sub-cohort of the testing cohort consisting of subjects that had at least two visits (timepoints).

*Predicting State- High Anxiety*. Receiver-operating characteristic (ROC) analyses between marker levels and anxiety state were performed by assigning subjects visits with an anxiety SAS-4 score of ≥60 into the high anxiety category, and subjects with STAI scores ≥55 in the high anxiety category. We used the pROC package of R (Xavier Robin et al. BMC Bioinformatics 2011). (Table 3, Figure 2). Additionally, a one-tailed t-test was performed between high anxiety group vs. the rest, and Pearson R (one-tail) was calculated between anxiety scores and biomarker levels.

*Predicting Trait- Future Psychiatric Hospitalization with Anxiety as a Symptom/Reason for Admission.* We conducted analyses for predicting future psychiatric hospitalizations with anxiety as a symptom/reason for the visit or admission in the first year following each testing visit, in subjects that had at least one year of follow-up in the VA system, in which we have access to complete electronic medical records. ROC analyses between biomarkers measures (cross-sectional, longitudinal) at a specific testing visit and future hospitalization admission were performed as described above, based on assigning if subjects had been admitted to the hospital with anxiety or not. Additionally, a one tailed t-test with unequal variance was performed between groups of subject visits with and without future hospitalization with anxiety. Pearson R (one-tail) correlation was performed between Hospitalization frequency (number of Hospitalization with anxiety divided by duration of follow-up) and marker levels. A Cox regression was performed using the time in days from the testing visit date to first hospitalization date in the case of patients who had been hospitalized, or 365 days for those who did not. The odds ratio was calculated such that a value greater than 1 always indicates increased risk for hospitalization, regardless if the biomarker is increased or decreased in expression.

We also conducted Cox regression and Pearson R analyses for all future hospitalizations with anxiety, including those occurring beyond one year of follow-up, in the years following testing (on average 7.35 years per subject, range 0.07 to 14.74 years), as these calculations, unlike the ROC and t-test, account for the actual length of follow-up, which varied from subject to subject. The ROC and t-test might in fact, if used, under-represent the power of the markers to predict, as the more severe psychiatric patients are more likely to move geographically and/or be lost to follow-up. The Cox regression was performed using the time in days from visit date to first hospitalization date in the case of patients who had hospitalizations with anxiety, or from visit date to last note date in the electronic medical records for those who did not.

**Therapeutics**

*Pharmacogenomics.*  We analyzed which of the top biomarkers for anxiety after Steps 1- 4 are known to be changed in expression by existing drugs in a direction opposite to the one in disease, using our CFG databases (Table S4).

*New drug discovery/repurposing*. We also analyzed which drugs and natural compounds are an opposite match for the gene expression signatures of our top biomarkers (n=19), using the Connectivity Map (<https://portals.broadinstitute.org>, Broad Institute, MIT) (Figure 3 and Table 4). Of note, not all the probesets from the HG-U133 Plus 2.0 array we used were present in the HGU-133A array used for the Connectivity Map. We stayed with exact probeset level matches, not gene level imputation.

**Report generation**

We present an example of how a report to doctors might look, using the above insights. We chose as a case study a visit from a female subject ( phchp328v1) with anxiety and depression who had died by suicide, a case previously discussed in a suicide biomarker paper of ours (Levey et al. 2016[^4^](#_ENREF_4)) (Figure 4). We used the panel of the top biomarkers for anxiety from Table 2 (n=19).

The raw expression values of the 19 biomarkers for 794 microarrays gene expression were Z-scored by gender and diagnosis. We calculated as thresholds the average expression value for a biomarker in the high anxiety group SAS-4 ≥60, and in the low anxiety group SAS-4 ≤ 40. The first average should be higher than the second average in increased biomarkers, and the reverse is true for decreased biomarkers. 15 out of 19 biomarkers were thus concordant.

We also calculated as thresholds the average expression value for a biomarker in the first-year hospitalizations group, and in the not hospitalized in the first-year group. We did the same thing for all future hospitalizations, and no future hospitalizations. The first average should be higher than the second average in increased biomarkers, and the reverse is true for decreased biomarkers. 18 out of 19 biomarkers were thus concordant for first year, and for all future.

The Z-scored expression value of each increased in expression biomarker was compared to the average value for the biomarker in the high anxiety group SAS-4 ≥60, and the average value of the low anxiety group SAS-4 ≤ 40, resulting in scores of 1 if above high anxiety, 0 if below low anxiety, and 0.5 if it was in between. The reverse was done for decreased in expression biomarkers. This digitalization of the scores was done to avoid overfitting to our particular cohort, and provide an easily understandable and interpretable readout for clinicians.

The digitized biomarkers were then added into a polygenic risk score, and normalized for the number of biomarkers in the panel, resulting in a percentile score for anxiety. We did the same thing for first year hospitalizations, and all future hospitalizations, generating a combined score for chronic anxiety risk.

The digitized biomarkers were also used for matching with existing psychiatric medications and alternative treatments (nutraceuticals and others). We used our large datasets and literature databases to match biomarkers to medications that had effects on gene expression opposite to their expression in high anxiety. Each medication matched to a biomarker got the biomarker score of 1, 0.5 or 0. The scores for the medications were added, normalized for the number of biomarkers that were 1 or 0.5 in that patient, resulting in a percentile match. Thus, psychiatric medications matched to the patient and ranked in order of impact on the panel.
